# A dual role for Ca_v_1.4 Ca^2+^ channels in the molecular and structural organization of the rod photoreceptor synapse

**DOI:** 10.7554/eLife.62184

**Published:** 2020-09-17

**Authors:** J Wesley Maddox, Kate L Randall, Ravi P Yadav, Brittany Williams, Jussara Hagen, Paul J Derr, Vasily Kerov, Luca Della Santina, Sheila A Baker, Nikolai Artemyev, Mrinalini Hoon, Amy Lee

**Affiliations:** 1 Department of Molecular Physiology and Biophysics, University of IowaIowa CityUnited States; 2 Iowa Neuroscience Institute, University of IowaIowa CityUnited States; 3 Pappajohn Biomedical Institute, University of IowaIowa CityUnited States; 4 Department of Neuroscience, University of Wisconsin-MadisonMadisonUnited States; 5 Department of Ophthalmology, University of California, San FranciscoSan FranciscoUnited States; 6 Department of Biochemistry, University of IowaIowa CityUnited States; 7 Department of OphthalmologyIowa CityUnited States; 8 Department of Ophthalmology and Visual Science, University of Wisconsin-MadisonMadisonUnited States; Universidad de La LagunaSpain; Oregon Health and Science UniversityUnited States

**Keywords:** photoreceptor, retina, calcium, synapse, Mouse

## Abstract

Synapses are fundamental information processing units that rely on voltage-gated Ca^2+^ (Ca_v_) channels to trigger Ca^2+^-dependent neurotransmitter release. Ca_v_ channels also play Ca^2+^-independent roles in other biological contexts, but whether they do so in axon terminals is unknown. Here, we addressed this unknown with respect to the requirement for Ca_v_1.4 L-type channels for the formation of rod photoreceptor synapses in the retina. Using a mouse strain expressing a non-conducting mutant form of Ca_v_1.4, we report that the Ca_v_1.4 protein, but not its Ca^2+^ conductance, is required for the molecular assembly of rod synapses; however, Ca_v_1.4 Ca^2+^ signals are needed for the appropriate recruitment of postsynaptic partners. Our results support a model in which presynaptic Ca_v_ channels serve both as organizers of synaptic building blocks and as sources of Ca^2+^ ions in building the first synapse of the visual pathway and perhaps more broadly in the nervous system.

## Introduction

It is well established that the primary function of voltage-gated Ca_v_ Ca^2+^ channels within axon terminals is to serve as a conduit for Ca^2+^ ions that trigger the fusion of neurotransmitter-laden vesicles with the presynaptic membrane (reviewed in Dolphin and Lee, 2020). Mutations in genes encoding presynaptic Ca_v_ channels cause neurological disorders such as epilepsy, migraine, and ataxia (Pietrobon, 2010). When studied in animal models, these mutations lead to aberrant synaptic transmission and alterations in network excitability (Tottene et al., 2009; Eikermann-Haerter et al., 2011). Due to the steep Ca^2+^-dependence of neurotransmitter release (Dodge and Rahamimoff, 1967), factors that modulate Ca_v_ channel function can dramatically alter synaptic output. Thus, efforts to understand the pathophysiological consequences of disease-causing mutations have largely centered on how they affect biophysical parameters of channel opening, closing, or inactivation (Pietrobon, 2010).

However, Ca_v_ channels are known to engage in pathways that do not involve their Ca^2+^ conductance. For example, voltage-dependent changes in the conformation of Ca_v_1.1 channels, rather than the incoming Ca^2+^ ions, initiate skeletal muscle contraction (Adams et al., 1990). Despite its importance for understanding fundamental cell biological processes in health and disease, the prevalence of non-conducting roles of Ca_v_ channels in neurons is poorly understood. Analyses of Ca_v_ knockout (KO) mice have helped illuminate the physiological functions of Ca_v_ channels (Pietrobon, 2005), however, this approach does not distinguish between the Ca^2+^-dependent and Ca^2+^-independent contributions of these channels. Moreover, complex double and triple-KO approaches are needed given that most synapses in the nervous system utilize more than one Ca_v_ subtype (Dolphin and Lee, 2020).

To overcome these hurdles, we focused on the Ca_v_1.4 L-type channel, which is the predominant Ca_v_ subtype in rod and cone photoreceptors in the retina (Mansergh et al., 2005; Chang et al., 2006; Liu et al., 2013). More than 140 mutations in the human *CACNA1F* gene encoding Ca_v_1.4 have been identified, many of which cause heterogeneous forms of vision impairment (Waldner et al., 2018). The absence of photoreceptor synaptic responses in Ca_v_1.4 KO mice has been attributed to a requirement for Ca_v_1.4 in the presynaptic release of glutamate (Mansergh et al., 2005; Chang et al., 2006; Liu et al., 2013). However, an additional contributing factor is that photoreceptor synapses do not form in Ca_v_1.4 KO mice (Liu et al., 2013; Zabouri and Haverkamp, 2013; Regus-Leidig et al., 2014). Does this developmental failure reflect a requirement for Ca_v_1.4-mediated Ca^2+^ signals or an alternative, non-conducting role for the Ca_v_1.4 protein in organizing the photoreceptor synapse? Here, we generated a mouse strain that expresses a non-conducting mutant form of Ca_v_1.4 to answer this question.

## Results and discussion

**Figure 1.**
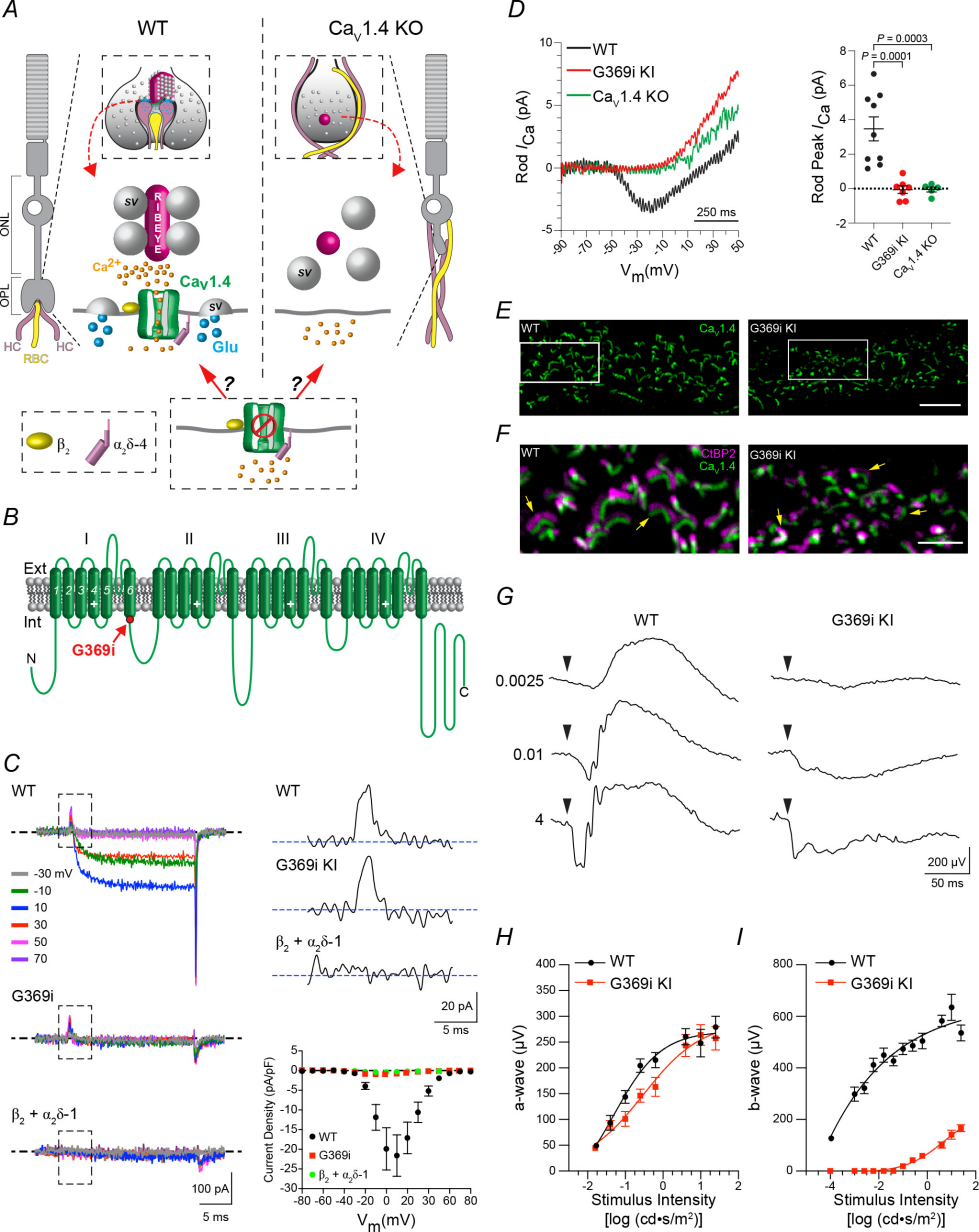
Characterization of G369i KI mouse strain to study the requirement of Ca_v_1.4 Ca^2+^ signals in rod synaptogenesis. (**A**), potential roles of Ca_v_1.4 in the assembly of rod synapses. In the outer plexiform layer (OPL) of WT mice, neurites of two horizontal cells (HCs) and one rod bipolar cell (RBC) invaginate into the rod spherule; Ca_v_1.4 clusters beneath the ribbon that tethers glutamate (Glu)-filled synaptic vesicles (SVs). In Ca_v_1.4 KO retina, ribbons do not form, and HC and RBC neurites sprout into the outer nuclear layer (ONL). The efficacy of a non-conducting Ca_v_1.4 mutant channel to support rod synaptogenesis will depend on whether Ca^2+^ influx through Ca_v_1.4 is required. (**B**), schematic of Ca_v_1.4 pore-forming subunit with four homologous repeats (I–IV) each with six transmembrane domains; location of the glycine insertion in domain I (G369i) is indicated. G369i mutation (red). (**C**), *left*, representative Ba^2+^ currents (*I*_Ba_) evoked by 20 ms voltage steps from −90 mV to the indicated voltages in transfected HEK293T cells. Boxed regions indicate gating currents evident in cells co-transfected with β_2_ + α_2_δ−1 and WT or G369i mutant channels but not in cells transfected with β_2_ and α_2_δ−1 alone. *Upper right*, representative gating currents from boxed regions (*left*) elicited by a voltage step to +70 mV. *Lower right*, graph shows I–V relationship for cells co-transfected with β_2_ + α_2_δ−1 alone (n = 5), or co-transfected with WT (n = 9) or G369i mutant channels (n = 6). Points represent mean ± SEM. (**D**), representative traces (*left*) and average amplitudes of peak rod Ca^2+^ currents (*I*_Ca_, *right*) recorded in acute slices from WT, G369i KI, and Ca_v_1.4 KO mouse retinas. Stimulus protocol was a 1 s voltage ramp from −90 to +50 mV. Points represent individual recorded rods with the mean and SEM indicated with bars. *P* values were determined by one-way ANOVA with Uncorrected Fisher’s Least Significant Difference post-hoc test. (**E**), confocal micrographs of the OPL of wild-type (WT) and G369i KI retina double-labeled with antibodies against Ca_v_1.4 and CtBP2 (CtBP2 labeling removed here for clarity). (**F**), expanded view of the boxed regions in *E* showing both CtBP2 and Ca_v_1.4 labeling. Arrows depict arc-shaped ribbons apposed to Ca_v_1.4 labeling. Scale bars, 5 μm. (**G**) representative voltage responses from flash ERGs recorded in dark- adapted WT (n = 4) and G369i KI (n = 4) mice. Arrowheads indicate time of flash. Numbers indicate flash intensities (cd•s /m^2^). (**H**, **I**), a-wave amplitudes (**H**) and b-wave amplitudes (**I**) measured from recordings obtained as in (**G**). Points represent mean ± SEM. Figure 1—source data 1.Traces corresponding to *I_Ca_* that were used for analyses of rod peak *I_Ca_* in Figure 1D are shown in the ‘.PDF’ file.Individual values obtained for peak *I_Ca_* in different cells are listed in the ‘.xlsx’ file.

Rod and cone photoreceptors communicate visual information via a triadic ‘ribbon’ synapse containing postsynaptic neurites of a depolarizing (ON) bipolar cell and two horizontal cells (HCs) (Masland, 2012; Figure 1A). The ribbon, an organelle that critically regulates the properties of glutamate release, is a morphological hallmark of this and other sensory synapses (Matthews and Fuchs, 2010). During the first postnatal week in mice, ribbons develop from spherical precursors and are concentrated in the outer plexiform layer (OPL) where mature rod and cone synapses are localized (Blanks et al., 1974; Regus-Leidig et al., 2009). In Ca_v_1.4 KO mice, the development of these synapses appears stunted in that spheres, rather than ribbons, are present at all developmental ages and often found in the outer nuclear layer (ONL) that normally contains only photoreceptor somas. The terminals of rods and cones (i.e., spherules and pedicles, respectively) (Figure 1A) of Ca_v_1.4 KO mice are misshapen, and a number of proteins typical of mature synapses are absent or aberrantly localized (Raven et al., 2008; Liu et al., 2013; Zabouri and Haverkamp, 2013; Regus-Leidig et al., 2014). If Ca_v_1.4 Ca^2+^ signals are needed for the assembly of photoreceptor synapses, then ribbons and key synaptic proteins should be lacking from photoreceptors of a mouse strain expressing a non-conducting mutant form of Ca_v_1.4, just as in Ca_v_1.4 KO mice.

**Figure 1—figure supplement 1.**
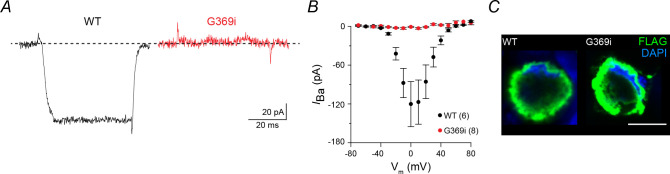
Characterization of G369i mutation in transfected HEK293T cells. (**A**) Representative Ba^2+^ currents (*I*_Ba_) evoked by 50 ms voltage step from −80 to 0 mV in HEK293T cells co-transfected with wild-type (WT) (*black*) or G369i (*red*) Ca_v_1.4 channels and auxiliary subunits β_2_ and α_2_δ−4. (**B**) Graph showing I–V relationship for cells co-transfected with WT or G369i mutant channels. *I_Ba_* was evoked by 50 ms voltage steps from −70 to +70 mV in +10 mV increments. *I_Ba_* is plotted against each test voltage (V_m_). Points represent mean ± SEM. Parentheses indicate number of cells. (**C**) HEK293T cells co-transfected as in *A* and immunofluorescently labeled using FLAG-tag antibodies. Scale bar = 10 μm.

**Figure 1—figure supplement 2.**
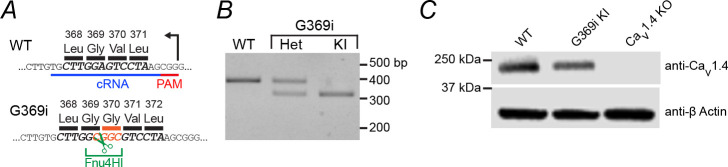
Characterization of the G369i KI mouse line. (**A**) CRISPR-Cas9 genome editing was used to insert a glycine residue between G369 and V370. The restriction site recognized by the endonuclease Fnu4HI was included in the repair template for genotyping. PAM, protospacer adjacent motif. cRNA, CRISPR RNA. (**B**) Gel image of PCR products that were amplified from genomic DNA followed by Fnu4HI digestion. Following digestion, two DNA fragments of ~330 base pairs (bp) and 60 bp (not shown) are present if the G369i mutation and Fnu4HI restriction site are within the *CACNA1F* gene. Note: the *CACNA1F* gene lies on the X chromosome, which precludes male mice from being heterozygous for the G369i mutation. (**C**) Western blot analysis of whole retina lysates collected from WT, G369i KI and Ca_v_1.4 KO mice. Blots were probed with antibodies against Ca_v_1.4 and β-actin. Bands corresponding to Ca_v_1.4 were present in WT and G369i KI retina lysates, but not lysates from Ca_v_1.4 KO mice.

To test this prediction, we deployed a strategy based on a disease-causing mutation in the Ca_v_1.3 L-type channel. The mutation results in the insertion of a glycine residue into transmembrane segment 6 of domain I (G369i, Figure 1B) and prevents Ca^2+^ influx through the channel during membrane depolarizations (Baig et al., 2011). We determined if this mutation has a similar effect in Ca_v_1.4 in electrophysiological recordings of transfected human embryonic kidney cells (HEK293T). The wild-type (WT) and G369i mutant channels were co-expressed with β_2_ and α_2_δ−4 subunits which are the major Ca_v_ auxiliary subunits forming Ca_v_1.4 complexes in photoreceptor synaptic terminals (Lee et al., 2015). G369i mutant channels were associated with the plasma membrane but did not mediate significant inward Ba^2+^ currents (*I_Ba_*) like WT channels (Figure 1—figure supplement 1). To functionally verify that G369i mutant channels reached the cell-surface, we analyzed ON gating currents (*I_gating_*), which result from the movement of charged voltage-sensing domains prior to channel opening. For this purpose, we utilized the α_2_δ−1 subunit which we find to produce larger current densities and better resolution of gating currents than with α_2_δ−4. Compared to robust peak *I_Ba_* densities mediated by WT channels (−20.70 ± 5.31 pA/pF), those in cells transfected with G369i mutant channels were negligible (−1.14 ± 0.11 pA/pF, p=0.03) and not significantly different from that in cells transfected only with β_2_ and α_2_δ−1 as negative controls (−0.56 ± 0.15 pA/pF, p=0.72, by Kruskal-Wallis test and post-hoc Dunn’s test; Figure 1C). *I_gating_* was evident for both WT and G369i mutant channels, but not in the negative control cells. To estimate the number of channels with functional voltage-sensors in the membrane, we analyzed the time integral of *I_gating_* evoked at the reversal potential (*Q_max_*, [Wei et al., 1994]). When normalized to cell size, *Q_max_* did not differ for WT (4.22 ± 1.34 fC/pF) and G369i mutant channels (3.22 ± 0.48 fC/pF, p=0.57 by unpaired t-test; Figure 1C). Thus, G369i prevents Ca^2+^ conductance but not cell-surface trafficking or voltage-sensor movements in Ca_v_1.4.

Armed with this knowledge, we incorporated the G369i mutation into Ca_v_1.4 channels in vivo by genome editing in mice (Figure 1—figure supplement 2). In agreement with our findings in the heterologous expression system, inward currents such as those measured in rods of WT mice, were not evident in those of the knock-in mouse strain (G369i KI). The absence of depolarization-evoked currents was similar in G369i KI mice and a mouse strain completely lacking Ca_v_1.4 expression (Ca_v_1.4 KO, Figure 1D). However, Ca_v_1.4 protein was detected in retinal lysates of G369i KI mice by Western blot (Figure 1—figure supplement 2). Moreover, immunofluorescence corresponding to G369i mutant channels was abundant in the OPL, where photoreceptor synapses normally form, and juxtaposed near the ribbon as in WT mouse retina (Figure 1E). Therefore, while it does not mediate voltage-dependent Ca^2+^ currents, the G369i mutant channel is expressed and targeted appropriately in photoreceptors of G369i KI mice. Electroretinograms (ERGs) of dark-adapted G369i KI mice lacked b-waves at all but the highest light intensities, consistent with a disruption of rod synaptic transmission (Figure 1I). A-waves, which represent light-dependent hyperpolarization of photoreceptors, were not significantly different in WT and G369i KI mice (Figure 1H). Thus, G369i KI mice show defects in photoreceptor function that are largely restricted to synaptic terminals.

**Figure 2.**
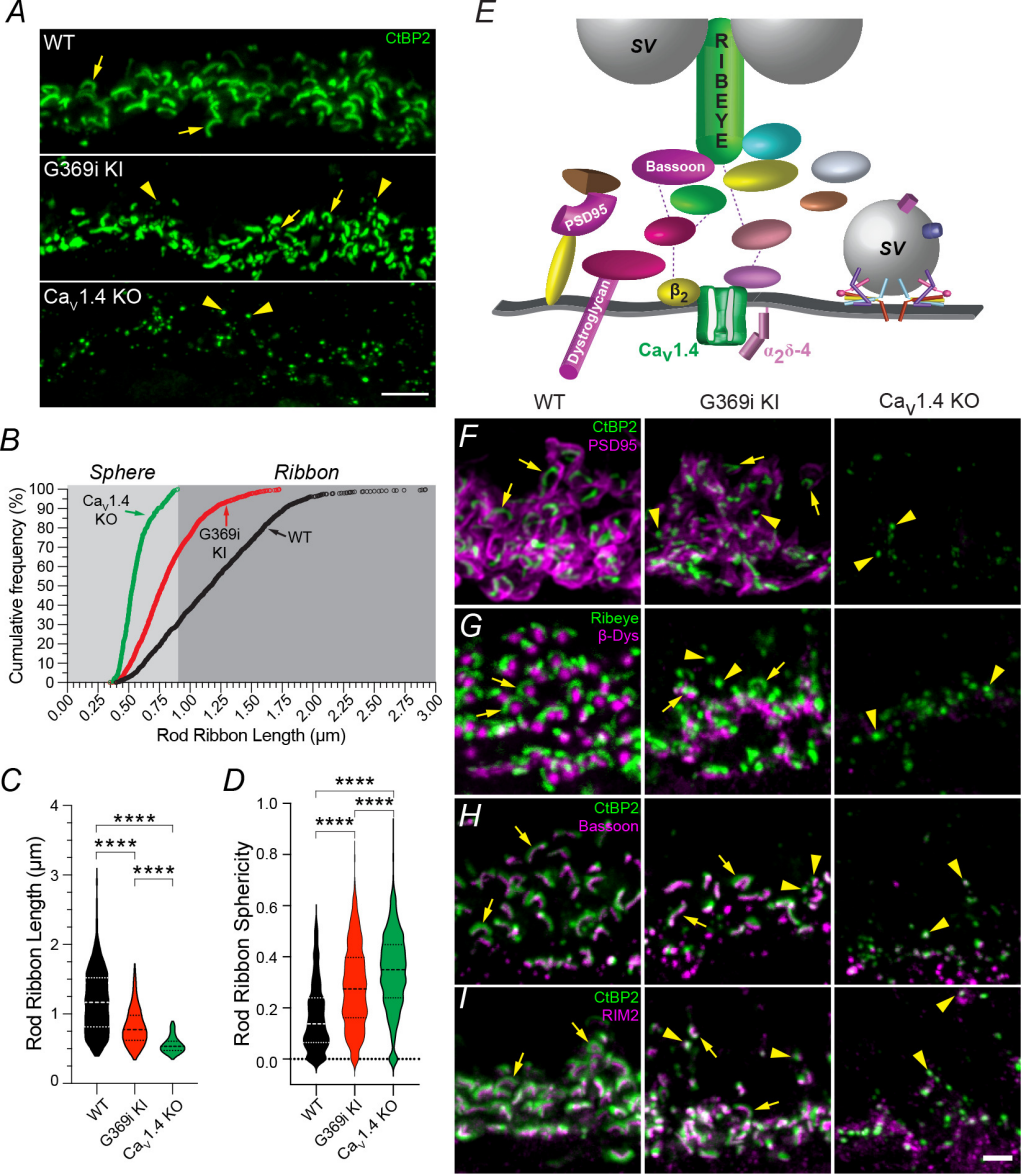
Rod ribbons still form in the absence of Ca_v_1.4 Ca^2+^ signals. (**A**) Confocal micrographs of the OPL of WT, G369i KI and Ca_v_1.4 KO mice labeled with antibodies against CtBP2 to mark ribbons (arrows). Arrowheads depict spheres resembling immature ribbons. (**B**) Cumulative frequency of rod ribbon lengths in WT, G369i KI, and Ca_v_1.4 KO. Ribbon states less than and greater than 0.95 µm were classified as spheres and ribbons, respectively. (**C**,** D**) Violin plots of rod ribbon lengths (**C**) and sphericity (**D**) obtained from dataset in **B**. **** p<0.001 by one-way ANOVA with Tukey’s multiple comparison post-hoc test. WT, n = 1264 ribbons; KO, n = 348 ribbons; G369i KI, n = 1322 ribbons. Data were collected from the retinas of at least six individual mice per genotype. (**E**) Schematic of a rod active zone and associated proteins. SV, synaptic vesicle. (**F–I**) Confocal micrographs (maximum z-projections) of the OPL of WT, G369i KI and Ca_v_1.4 KO mice double-labeled with antibodies against CtBP2 and PSD95 (**F**), Ribeye and β-dystroglycan (β-Dys), (**G**) CtBP2 and Bassoon (**H**) and CtBP2 and RIM2 (**I**). Arrows and arrowheads depict ribbons and spheres, respectively. Scale bars, 5 μm in **A**, 2 μm in **F**-**I**. Figure 2—source data 1.The image archive contains all images used in quantitative analyses in Figure 2B–D.The data were exported as 8-bit ‘tif’ files (1600 × 1600 pixels). Values obtained for individual data points (and outlier analysis) for summary graphs in Figure 2B–D are contained in ‘.xlsx’ files.

To define the contribution of Ca_v_1.4 Ca^2+^ signals to the organization of the photoreceptor synapse, we compared the molecular composition and structure of photoreceptor synapses in the retinas of WT, G369i KI, and Ca_v_1.4 KO mice. We focused on rod terminals due to their simpler synaptic organization (i.e. one large, arc-shaped ribbon and a single type of postsynaptic bipolar cell) as compared to cones (i.e. multiple short ribbons and types of postsynaptic bipolar cells). Furthermore, rod synapses are more severely disrupted than cone synapses when the presynaptic abundance of Ca_v_1.4 is compromised (Ball et al., 2002; Katiyar et al., 2015; Wang et al., 2017; Kerov et al., 2018). We first compared the prevalence of ribbons and spheres using antibody labeling for CtBP2, a component of the major ribbon protein Ribeye (Schmitz et al., 2000; Figure 2A). We restricted analyses to CtBP2-labeled structures in rod spherules by excluding those in cone arrestin-labeled cone pedicles. The minimum ribbon length was arbitrarily set at 0.95 μm based on the absence of values higher than this in Ca_v_1.4 KO retina. While shorter on average than in WT retina, arc-shaped ribbons were plentiful in the OPL of G369i retina (Figure 2B–D) and co-clustered with proteins characteristic of mature rod synapses (Figure 2E) such as PSD-95 and β-dystroglycan (Koulen et al., 1998; Blank et al., 1997), which were undetectable in the OPL of Ca_v_1.4 KO retina (Figure 2F,G). Bassoon, a protein involved in ribbon morphogenesis (tom Dieck et al., 2005), and RIM2, a protein implicated in regulating Ca_v_1.4 at rod synapses (Grabner et al., 2015), were tightly clustered with ribbons in G369i KI as in WT retina (Figure 2H,I). Thus, although leading to shorter ribbons, the presynaptic assembly of rod synapses can proceed without Ca_v_1.4 Ca^2+^ signals.

**Figure 3.**
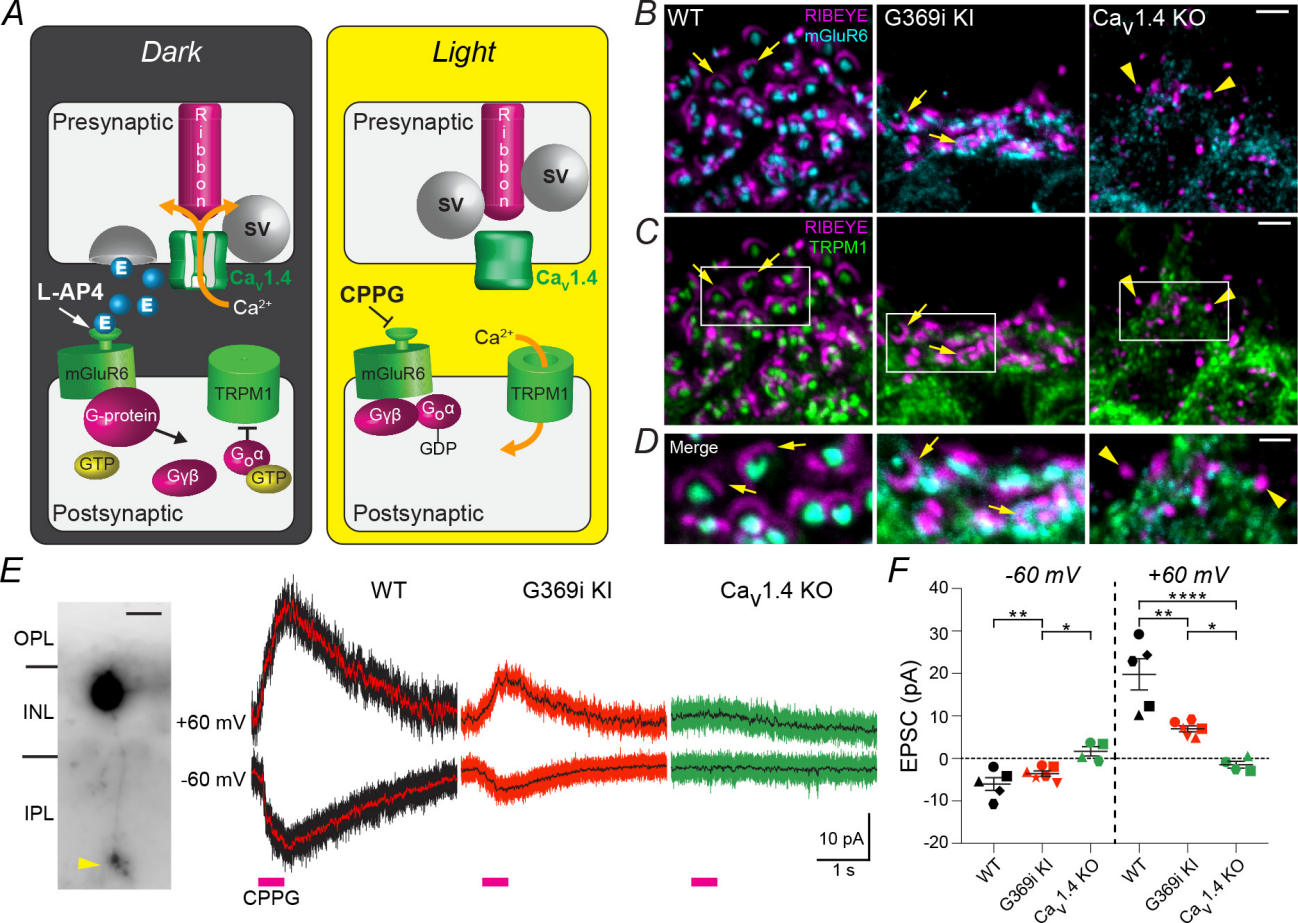
Postsynaptic signaling at rod synapses is partially intact in the absence of Ca_v_1.4 Ca^2+^ signals. (**A**), schematic illustrating synaptic transmission at rod-RBC synapses. In the dark, rods are depolarized, which activates Ca_v_1.4 and Ca^2+^-triggered glutamate (E) release. Activation of the mGluR6/G-protein pathway by glutamate or L-AP4 inhibits TRPM1-dependent channels. Light signals or the mGluR6 antagonist CPPG activate the ON pathway by disinhibiting the TRPM1-dependent conductance. (**B–D**), confocal micrographs of the OPL of WT, G369i KI and Ca_v_1.4 KO mice triple-labeled with antibodies against Ribeye, mGluR6, and TRPM1. In *B*,*C*, only signals for Ribeye and mGluR6 (**B**) or TRPM1 (**C**) are shown for clarity. (**C**) Higher magnification views of boxed regions are shown in the merged image (**D**). Arrows and arrowheads depict ribbons and spheres, respectively. (**E–F**), whole-cell patch clamp electrophysiology of RBCs recorded in WT, G369i KI, and Ca_v_1.4 KO mouse retinal slices. *E*, left, representative image of Lucifer Yellow-filled RBC showing typical lobular-shaped terminal (arrowhead). *Right*, representative current responses to CPPG puffs in RBCs held at −60 mV and +60 mV. Lines within each current trace are low-pass FFT filtered data. *F*, peak amplitudes of CPPG-evoked EPSCs. Each shape represents individual cells recorded at −60 and +60 mV. Bars represent mean ± SEM. ****p<0.0001, **p<0.01, *p<0.05, by one-way ANOVA with Fishers least significant difference post-hoc test. Scale bars, 2 μm in *B*,*C*, 1 μm in *D*, 15 μm in *E*. Figure 3—source data 1.Traces corresponding to EPSCs that were used for analyses of EPSC amplitudes in Figure 3F are included in the ‘.PDF’ file; individual values obtained for EPSC amplitudes in different cells are listed in the ‘.xlsx’ file.

We next determined if postsynaptic elements were also intact at G369i KI rod synapses. At the depolarized voltage of photoreceptors in darkness, Ca_v_1.4-dependent glutamate release activates a metabotropic glutamate receptor (mGluR6), which suppresses the activity of TRPM1-dependent channels (Shiells et al., 1981; Shen et al., 2009). Light-dependent hyperpolarization of photoreceptors reduces Ca_v_1.4 Ca^2+^ signals, which slows the rate of glutamate release, thus disinhibiting the postsynaptic conductance and enabling excitatory postsynaptic currents (EPSCs) that communicate light signals via the ON pathway (Shiells et al., 1981; Figure 3A). In agreement with previous work (Koike et al., 2010), mGluR6 was clustered postsynaptic to rod ribbons, and TRPM1 was situated at the tips of RBC dendrites in WT retina (Figure 3B–D). Although the subcellular distribution of the postsynaptic proteins was partially obscured by the diffuse pattern of TRPM1 labeling, arc-shaped ribbons in apposition to colocalized TRPM1 and mGluR6 could clearly be distinguished in G369i KI retina but not in Ca_v_1.4 KO retina (Figure 3B–D). To test if this signaling pathway was functional in RBCs of G369i KI mice, we used an agonist and antagonist of mGluR6 (L-AP4 and CPPG, respectively) to simulate light responses in RBCs (Shen et al., 2009; Figure 3A). As expected (Morgans et al., 2009; Shen et al., 2009), the CPPG-evoked EPSC was robust and outwardly rectifying in WT RBCs (Figure 3E,F). Although significantly lower in amplitude compared to WT RBCs, this EPSC was measurable in all RBCs that were recorded in G369i KI mice but was never detected in any of the RBC recordings in Ca_v_1.4 KO mice (Figure 3E,F). These results indicate that the molecular components of the postsynaptic signaling complex in RBCs can be assembled in the absence of Ca_v_1.4 Ca^2+^ signals, albeit less efficiently than in their presence.

**Figure 4.**
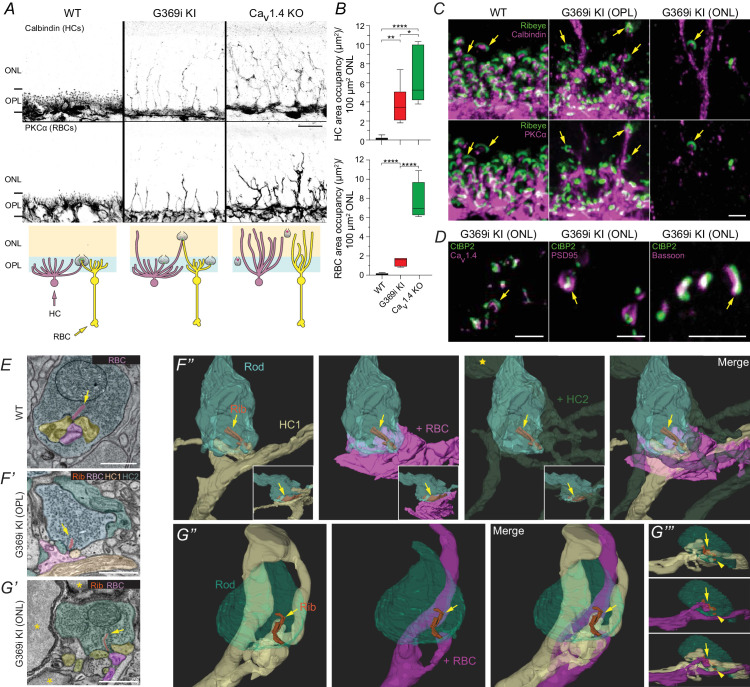
Rod synapses lack invaginating HC and RBC neurites in the absence of Ca_v_1.4 Ca^2+^ signals. (**A**), confocal micrographs of the ONL and OPL of WT, G369i KI and Ca_v_1.4 KO retinas immunolabeled for calbindin and PKCα. The schematic below illustrates neurite sprouting in each genotype. (**B**), quantification of the area in the ONL occupied by HCs (calbindin) or RBCs (PKCα). One-way ANOVA with Fishers least significant difference post-hoc test. ****p<0.0001, **p<0.01, *p<0.05. n = 6 mice for each genotype. (**C**), confocal micrographs of the OPL of WT and the ONL and OPL of G369i KI retinas immunolabeled for Ribeye and Calbindin (*upper panels*) or PKCα (*lower panels*). (**D**), confocal micrographs of the ONL of G369i KI retinas that were immunolabeled for CtBP2 and Ca_v_1.4 (*left*), PSD95 (*middle*) or Bassoon (*right*). (**E**), TEM image of a rod terminal in a WT retina. (**F,G**), serial block-face scanning electron microscopy images (**F’,G’**) and 3D reconstructions (**F’’–G’’’**) of rod terminals in a G369i KI retina. Rod terminals located within the OPL (**F**) and ONL (**G**). The yellow star *F’’* indicates a neurite from HC2 sprouting into the ONL. Insets in *F’’* are a rotated view. *G’’’* is a side view of *G’’*. Arrows depict anchored ribbons. Arrowheads in G’’’ depict additional ribbon. Asterisks in G’ indicate rod somas. Scale bars, 10 μm in *A*, 2 μm in *C-D*, 1 μm in *E*,*F’*,*G’*. Rib, ribbon. RBC, rod bipolar cell. HC, horizontal cell. Figure 4—source data 1.The image archive contains all images used in quantitative analyses in Figure 4B.The data were exported as 8-bit ‘tif’ files (800 × 600 pixels). Values obtained for individual data points (and outlier analysis) for summary graphs in are contained in ‘.xlsx’ files.

Sprouting of HC and RBC neurites out of the OPL and into the ONL, and the appearance of ectopic ribbons and other presynaptic proteins in the ONL, occurs as a result of presynaptic functional impairments in rods and cones (McCall and Gregg, 2008). Thus, the prevailing view has been that synaptic transmission is needed to maintain the laminar organization of photoreceptor synapses in the OPL. Consistent with this notion, HC and RBC sprouting (Figure 4A) are particularly prominent in Ca_v_1.4 KO mice, which lack evidence of photoreceptor transmission (Chang et al., 2006; Raven et al., 2008; Liu et al., 2013; Zabouri and Haverkamp, 2013). If these defects arise solely from the absence of Ca_v_1.4 Ca^2+^ signals that trigger glutamate release, the severity of neurite sprouting should be similar in G369i KI mice as in Ca_v_1.4 KO mice. Contrary to this prediction, HC and RBC neurite sprouting was less prominent in G369i KI mice than in Ca_v_1.4 KO mice (Figure 4A,B) with most of the synapses situated normally in the OPL (Figure 4C). These results indicate a supporting, but non-essential, role for Ca_v_1.4 Ca^2+^ signals and/or glutamatergic transmission for the proper lamination of rod synapses within the OPL.

**Figure 4—figure supplement 1.**
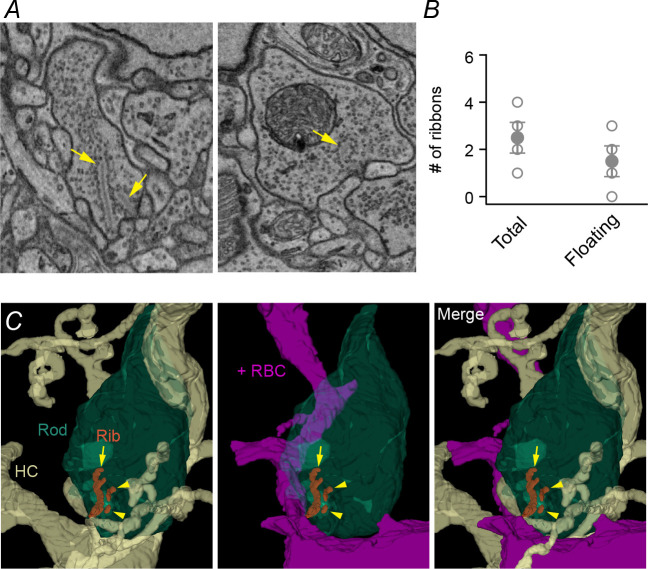
Multiple ribbons are found in rod terminals of G369i KI mice. (**A**) Electron micrographs of rod terminals with multiple ribbons (*left*) and a club-shaped ribbon (*right*). Arrows depict separate ribbons. (**B**) Number of total and floating ribbons in four rod terminals of G369i KI mice. Filled circles represent the means. Bars represent the SEM. (**C**) Additional SBFSEM reconstruction of a G369i KI spherule at the OPL/ONL border. Panels are labeled as in Figure 4F–G.

**Figure 4—figure supplement 2.**
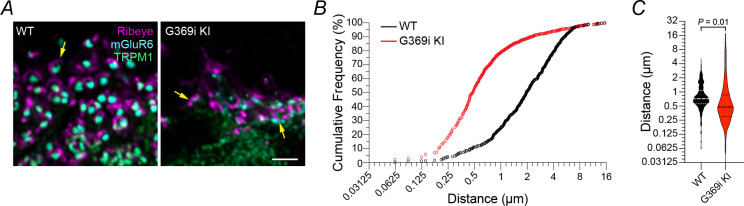
Nearest-neighbor analysis of Ribeye- and mGluR6-labeled structures. (**A**) Deconvolved confocal images from Figure 2B-D. Arrows indicate arc-shaped ribbons in apposition to mGluR6/TRPM1. (**B**) Cumulative frequency of distances between Ribeye and mGluR6. (**C**) Violin plot of Ribeye-mGluR6 distances. Solid lines represent the medians. Dashed lines represent the quartiles. p-value determined by unpaired *t*-test. WT, n = 864. G369i KI, n = 1390. Figure 4—figure supplement 2—source data 1.The image archive contains all images used in quantitative analyses in Figure 4—figure supplement 2B; Figure 4—figure supplement 2C.The data were exported as 8-bit ‘tif’ files (1024 × 1024 pixels). Values for individual data points (and outlier analysis) for summary graphs in Figure 4—figure supplement 2B are Figure 4—figure supplement 2C contained in excel files.

Compared to the spherical appearance of ribeye-labeled structures in the OPL/ONL of Ca_v_1.4 KO and other mouse strains with presynaptic dysfunction (Chang et al., 2006; McCall and Gregg, 2008; Raven et al., 2008; Liu et al., 2013; Zabouri and Haverkamp, 2013), those in the ONL of G369i KI mice exhibited mature ribbon morphology and were associated with synaptic proteins as in the WT OPL (Figure 4C,D). To further analyze the seemingly normal organization of these synapses in the OPL and ONL, we performed 3-dimensional (3D) reconstructions of spherules in G369i KI retina via serial block-face scanning electron microscopy (Figure 4E–G). Each of the four rod terminals sampled from G369i KI retina had a single ribbon that was anchored at the presynaptic membrane and was apposed to a triad of postsynaptic partners (Figure 4F–G). However, several peculiarities were observed. First, three of the four spherules that were analyzed contained more than one ribbon, rather than the single ribbon characteristic of rod terminals in the WT mouse retina (Blanks et al., 1974; Figure 4E–G). Second, some ribbons appeared club-shaped (Figure 4—figure supplement 1). Third, at least one of the extraneous ribbons was ‘floating’ rather than anchored near the presynaptic membrane. These alterations of ribbon architecture in G369i KI rod spherules resemble the types of ribbon plasticity caused by reductions in presynaptic Ca^2+^ in mouse rods due to light or Ca^2+^ chelators (Spiwoks-Becker et al., 2004; Dembla et al., 2020). Thus, the lack of Ca_v_1.4 Ca^2+^ signals in G369i KI rods could have caused fragmentation of the anchored ribbon, leading to the appearance of additional short, floating ribbons.

A particularly prominent anomaly of the reconstructed spherules was an alteration in postsynaptic architecture. Normally, within the invagination of mouse rod spherules are two HC neurites and an intervening RBC neurite (Figure 4E). Compared to HCs, the RBC neurite tip and the resident mGluR6 receptors are maintained relatively distant—hundreds of nanometers—from the release site (Rao-Mirotznik et al., 1995). This complex organization is thought to be critical for controlling glutamate release volume and spillover dynamics, allowing for differential activation of distinct glutamate receptor populations (Petralia et al., 2018). For spherules in the OPL as well as in the ONL of G369i KI retina, branching neurites of HCs and RBCs formed non-invaginating triadic contacts close to the anchored presynaptic ribbon (Figure 4F,G), rather than invading rod spherules as in WT retina (Figure 4E). Nevertheless, immunolabeling for mGluR6 remained clustered near ribbons in G369i KI retina (Figure 3B; Figure 4—figure supplement 2), suggesting that pre and postsynaptic apposition is maintained even in the absence of the normal spacing between RBC tips and the release sites. In agreement with these findings, mGluR6 labeling was significantly closer to that for ribbons in G369i KI than in WT retina as revealed by nearest-neighbor analyses (Figure 4—figure supplement 2). Thus, while dispensable for postsynaptic partner selection at rod synapses, Ca_v_1.4 Ca^2+^ signals are required for the invaginating arrangement of the corresponding neurites within the rod spherule and their proximity to release sites.

Like other presynaptic Ca_v_ channels, Ca_v_1.4 is expected to interact directly or indirectly with a diverse array of proteins (Dolphin and Lee, 2020), some of which are required for ribbon formation in photoreceptors (tom Dieck et al., 2005; Kiyonaka et al., 2012). Thus, the Ca_v_1.4 complex could act as a central scaffold for protein interactions that drive the assembly of the ribbon and associated components of the active zone. Alternatively, but not mutually exclusively, voltage-dependent conformational changes in the Ca_v_1.4 protein, which are intact in G369i mutant channels (Figure 1C), could be involved. For example, Ca_v_1.4 could conformationally couple to intracellular Ca^2+^ release channels, as Ca_v_1.1 does in skeletal muscle (Adams et al., 1990), which could represent a Ca^2+^ source that aids presynaptic development.

A limitation of our study is that the mutant G369i channel was expressed in all retinal cell-types that normally express Ca_v_1.4. In addition to photoreceptors, Ca_v_1.4 appears to be expressed in bipolar cells and at much lower levels in other cell-types in mouse retina (Yan et al., 2020). Because we find specific immunoreactivity for Ca_v_1.4 in the outer retina to be limited to photoreceptor terminals (Liu et al., 2013), we favor the interpretation that Ca_v_1.4 protein is not present to any large extent within bipolar cell soma or dendrites and that the postsynaptic structural defects of G369i KI rod synapses result primarily from the absence of presynaptic Ca_v_1.4 Ca^2+^ signals in rods. In addition to supporting glutamatergic transmission, presynaptic Ca_v_1.4 Ca^2+^ signals could strengthen trans-synaptic signaling via cell-adhesion molecules such as NGL-2 in HCs (Soto et al., 2013) and ELFN1 and pikachurin in RBCs (Sato et al., 2008; Wang et al., 2017), which collectively enable the invagination of the corresponding neurites into the rod terminal. Considering that rod ribbons are shorter in G369i KI than in WT retina (Figure 2B), Ca_v_1.4 Ca^2+^ signals may also regulate the activities of Ca^2+^ sensitive proteins such as GCAP2, which binds to Ribeye and controls ribbon stability in a Ca^2+^-dependent manner (Schmitz, 2014).

There are notable parallels and distinctions in the rod phenotypes of G369i KI mice and those lacking expression of either of the auxiliary subunits of Ca_v_1.4, β_2_ or α_2_δ−4. Ca_v_β and α_2_δ subunits are known to facilitate the forward trafficking and maintenance of Ca_v_ channels in the plasma membrane (Dolphin and Lee, 2020). Thus, it is not surprising that β_2_ KO and α_2_δ−4 KO mice exhibit severely diminished Ca_v_1.4 Ca^2+^ signals, and similar to G369i KI mice (Figures 1G–I and 4A–C), there is evidence for a loss of rod synaptic transmission in ERGs, and significant sprouting of postsynaptic RBCs and HCs into the ONL in these mouse strains (Ball et al., 2002; Katiyar et al., 2015; Wang et al., 2017; Kerov et al., 2018). However, a striking difference is that ribbons and other aspects of the presynaptic complex are preserved in G369i KI but not in β_2_ KO or α_2_δ−4 KO mice (Ball et al., 2002; Katiyar et al., 2015; Wang et al., 2017; Kerov et al., 2018). Because we find significant levels of the mutant Ca_v_1.4 protein within rod terminals of G369i KI mice (Figure 1E,F), which is not the case in β_2_ KO or α_2_δ−4 KO mice (Ball et al., 2002; Katiyar et al., 2015; Wang et al., 2017; Kerov et al., 2018), we propose that a minimal complement of Ca_v_1.4 channels is needed to enable ribbon morphogenesis.

How synapses achieve their diverse forms and functions remains a fundamental question in neuroscience. Current evidence indicates that most synapses are assembled by intrinsic programs involving a variety of scaffolding proteins and cell-adhesion molecules (Kurshan and Shen, 2019), with ensuing patterns of neurotransmitter release needed primarily to instruct further refinements in synapse morphology and connectivity (Sando et al., 2017; Sigler et al., 2017). Our study identifies Ca_v_1.4 as a nexus for both activity-dependent and independent mechanisms that drive the maturation of the rod synapse. Elucidating the signaling pathways that underlie the dual functions of Ca_v_1.4 is an important challenge for future studies.

## Materials and methods

### Mice

All mouse protocols and procedures were approved by the University of Iowa Institutional Animal Care and Use Committee. To generate the G369i KI mouse, a CRISPR RNA (crRNA) was designed to target exon 7 of the mouse *Cacna1*f gene (5’-CTTGGAGTCCTAAGCGG). The repair template was designed to contain a glycine codon insertion corresponding to amino acid residue 369 with a silent point mutation to include the Fnu4HI restriction site (Figure 1—figure supplement 2). The Cas9 protein, synthetic crRNA, trans-activating crRNA (tracrRNA), and repair template (PAGE-purified donor oligo) were co-injected into pronuclei of mouse zygotes (C57BL6/J [RRID:IMSR_JAX:000664] × C57BL/6SJL[F1] [RRID:IMSR_JAX:100012]) by the University of Iowa Genome Editing Facility. Genotyping of mice was performed by PCR (forward primer 5'-TGCCTTGGGTGTACTTTGTG, reverse primer 5'-CCGAGCTCAGATGGAGTTTATG) followed by digestion of the PCR product with the Fnu4HI restriction enzyme. Mice were backcrossed to the C57BL/6J parental strain for 10 generations. Since the *Cacna1f* gene encoding Ca_v_1.4 is on the X chromosome, only males of each genotype were used for experiments. WT littermates were used as controls. Ca_v_1.4 KO (RRID:IMSR_JAX:017761) mice were obtained from The Jackson Laboratory.

### Immunohistochemistry

Mice at postnatal day 42 (P42) were euthanized with a lethal dose of isoflurane followed by cervical dislocation. Eyes were enucleated and hemisected. The remaining eye cups were fixed in ice-cold 4% paraformaldehyde for 30 min. Fixed eye cups were washed three times with 0.1 M PB containing 1% glycine. Eye cups were infused with 30% sucrose at 4°C overnight and frozen in a 1:1 (wt/vol) mixture of Optimal Cutting Temperature compound (OCT, Sakura Finetek, Torrence, CA) and 30% sucrose in a dry ice/isopentane bath. Eye cups were cryosectioned at 20 µm on a Leica CM1850 cryostat (Leica Microsystems, Wetzlar, Germany), mounted on Superfrost plus Micro Slides (VWR, Radnor, PA), dried for 5 to 10 min at 42°C, and stored at −20°C. Slides with mounted cryosections were warmed to room temperature, washed with 0.1 M PB for 30 min to remove the OCT/sucrose mixture, and blocked with dilution solution (DS, 0.1 M PB/10% goat serum/0.5% Triton-X100) for 15 min at room temperature. All remaining steps were carried out at room temperature. All primary antibodies and appropriate secondary antibodies were diluted in DS at concentrations specified in Table 1. Sections were incubated with primary antibodies for 1 hr or overnight and then washed five times with 0.1 M PB. Sections were then incubated with secondary antibodies for 30 min and then washed five times with 0.1 M PB. Labeled sections were mounted with #1.5 coverslips (Leica) using Fluoromount-G (Electron Microscopy Sciences, Hatfield, PA).

**Table 1.** Antibodies used in this study.

Antibody	Host/clonality	Manufacturer	Cat no.	RRID
Bassoon	ms-IgG2A	Thermo Fisher Scientific	MA1-20689	AB_2066981
Cone Arrestin	rabbit	Millipore	AB15282	AB_1163387
Calbindin (D-28K)	mouse-IgG1	Sigma	C9848	AB_476894
PKCα	mouse-IgG2A	Invitrogen	MA1-157	AB_2536865
	rabbit	UC Santa Cruz	SC-208	AB_2168668
Ctbp2	mouse-IgG1	BD Biosciences	612044	AB_399431
Ribeye	rabbit	Synaptic Systems	192 103	AB_2086775
Psd95	mouse-IgG2A	UC Davis/NIH NeuroMab	75–028	AB_2292909
RIM2	rabbit	Synaptic Systems	140–103	AB_887776
mGluR6-366	mouse	Dr. Theodore Wensel	N/A	N/A
TRPM1	mouse-IgG1	Dr. Theodore Wensel	N/A	N/A
Ca_v_1.4	rabbit	Lee Lab Ab167	N/A	AB_2650487

### Image acquisition

Retinas that had been immunofluorescently labeled were visualized using an upright Olympus FV1000 confocal microscope (Tokyo, Japan) equipped with an UPLFLN 100x oil immersion objective (1.3 NA). Images were captured using the Olympus FluoView software package. Acquisition settings were optimized using a saturation mask to prevent signal saturation prior to collecting 16-bit, confocal z-stacks with voxel size of 0.09μm×0.09μm×0.2μm(X×Y×Z). All confocal images presented are maximum z-projections.

### Molecular biology and transfection

The cDNAs used were Ca_v_1.4 (GenBank: A930034B14), β_2a_ (GenBank: NM_053851), β_2X13_ (GenBank: KJ789960), and α_2_δ−4 (GenBank: NM_172364) in pcDNA3.1 (Lee et al., 2015). To create the Ca_v_1.4 cDNA with the G369i mutation, a codon insertion corresponding to the glycine insertion at residue G369 was generated with the HiFi DNA Assembly Cloning System (New England Biolabs) using Ca_v_1.4 cDNA as the PCR template and the primers a1FbegNotF: 5'-TATATGCGGCCGCCCACCATGGATTAC-3’, Fr1G369insR: 5'-CTTAGGACACCTCCAAGCACAAGGTTGAGG-3’, Fr2G369insF: 5'-TTGGAGGTGTCCTAAGCGGGGAGTTC-3’, a1FBsrGIr: 5'-TTTAGGCAGCGTGTACAGCTAGCCATGGTCC-3’. All constructs were verified by DNA sequencing before use. Human embryonic kidney 293 T (HEK293T) cells (American Type Culture Collection, Manassas, Virginia) were cultured in Dulbecco’s Modified Eagle’s Medium (Thermo Fisher Scientific, Waltham, MA) with 10% FBS (VWR) at 37°C in 5% CO_2_. At 70–80% confluence, the cells were co-transfected with cDNAs encoding mouse Ca_v_1.4 (1.8 μg) β_2X13_ (0.6 μg), α_2_δ−4 (0.6 μg), and enhanced GFP in pEGFP-C1 (0.1 μg) using FuGENE six transfection reagent (Promega, Madison, WI) according to the manufacturer’s protocol. Cells treated with the transfection mixture were incubated at 37°C for 24 hr. Cells were then incubated at 30°C for an additional 24 hr before beginning experiments.

### Solutions

All reagents were purchased from Sigma-Aldrich unless otherwise stated. HEK293T external recording solution contained the following (in mM): 140 Tris, 20 BaCl_2_, 1 MgCl_2_, pH 7.3. HEK293T internal recording solution contained the following: 140 NMDG, 10 HEPES, 2 MgCl_2_, 2 Mg-ATP, 5 EGTA, pH 7.3. The retina slicing solution was continuously bubbled with 100% O_2_ and contained the following: Ames’ Medium with L-glutamine supplemented with (in mM): 15 NaCl, 10 HEPES, 80 U/mL penicillin, 80 µg/mL streptomycin, pH 7.4. The extracellular slice recording solution was continuously bubbled with 95% O_2_/5% CO_2_ and contained the following: Ames’ Medium with L-glutamine, 5 NaCl, 23 NaHCO_3_, 80 U/mL penicillin, 80 µg/mL streptomycin. The intracellular pipette solution for recording rod photoreceptor Ca^2+^ currents (*I*_Ca_) contained the following: 120 CsMeSO_4_, 10 tetraethylammonium (TEA) Cl, 1 CaCl_2_, 1 MgCl_2_, 5 Mg-ATP, 0.5 Na-ATP, 5 phosphocreatine, 10 HEPES, 2 ethylene glycol-bis(β-aminoethyl ether)-N,N,N′,N′-tetraacetic acid (EGTA), 2 N-(2,6-Dimethylphenylcarbamoylmethyl)triethylammonium bromide (QX 314 bromide), 0.1 mg/mL Lucifer Yellow, pH 7.35. The intracellular pipette solution for recording rod bipolar cells contained the following: 105 CsMeSO_4_, 20 TEA-Cl, 4 Mg-ATP, 0.4 Na-ATP, 10 phosphocreatine, 10 HEPES, 2 1,2-bis(o-aminophenoxy)ethane-N,N,N′,N′-tetraacetic acid (BAPTA), 10 glutamic acid, 0.1 mg/mL Lucifer Yellow, pH 7.35.

The mGluR6 agonist L-(+)−2-Amino-4-phosphonobutyric acid (L-AP4, Tocris) and antagonist (*RS*)-α-Cyclopropyl-4-phosphonophenylglycine (CPPG, Tocris) were dissolved in 4 mM NaOH and 100 mM NaOH, respectively. L-AP4 and CPPG were diluted into extracellular slice recording solution and retina slicing solution, respectively. The pH of each solution was checked after the addition of L-AP4 or CPPG and adjusted if necessary.

### Patch clamp electrophysiology

Transfected HEK293T whole-cell voltage clamp recordings were performed 48 to 72 hr after transfection using an EPC-9 amplifier and Patchmaster software (HEKA Elektronik, Lambrecht, Germany). Patch pipette electrodes with a tip resistance between 2 and 4 MΩ were pulled from thin-walled borosilicate micropipettes (VWR) using a P-97 Flaming/Brown Puller (Sutter Instruments, Novato, CA). A reference Ag/AgCl wire was placed into the culture dish mounted on an inverted Olympus IX70 microscope. Recordings were performed at room temperature (22–24°C). The series resistance was compensated up to 70%, and passive membrane leak subtraction was conducted using a p/−4 protocol. Whole-cell *I*_Ba_ of transfected HEK293T cells were evoked for 50 ms with incremental 10 mV steps from −80 mV to +80 mV from a holding voltage of −90 mV. Data were analyzed using IgorPro 6.0 (WaveMetrics, Portland OR, USA).

Whole-cell voltage clamp recordings of rods and RBCs were performed using and EPC-10 amplifier and Patchmaster software (HEKA). Patch pipette electrodes with a tip resistance between 12 and 15 MΩ for rods and between 7 and 9 MΩ for RBCs were pulled from thick-walled borosilicate glass (1.5 mm outer diameter; 0.84 mm inner diameter; World Precision Instruments, Sarasota, FL, USA) using a P-97 Flaming/Brown Puller (Sutter Instruments, Novato, CA). To prepare retinal slices, adult mice (6–8 weeks old) were euthanized using a lethal dose of isoflurane followed by cervical dislocation. Eyes were enucleated, placed into cold Ames’ media slicing solution, and hemisected. Following removal of the vitreous, the retina was separated from the back of the eye. Central retina was isolated, molded into low-melt agarose (Research Products International, Mount Prospect, IL, USA) and mounted in a Leica VT1200s vibratome (Leica Biosystems). Vertical retinal slices (~250 µM) were anchored in a recording chamber, placed onto a fixed stage (Sutter Instruments), and positioned under an upright Olympus BX51WI microscope with a water-immersion objective 40X (0.8 NA). A custom gravity perfusion system was used to deliver extracellular slice recording solution at a flow rate of 2 ml/min. Cell bodies of rods and RBCs identified based on their morphology and location using IR-DIC optics and a CCD Camera (Hamamatsu Photonics, Shizuoka, Japan) controlled with µManager software (doi:10.14440/jbm.2014.36). Cells were filled with Lucifer Yellow during each recording, and the morphology of each cell recorded was confirmed post hoc using wide-field fluorescence. A reference Ag/AgCl pellet electrode was placed in 3 M KCl and connected to the recording chamber with a 3 M KCl agarose bridge. Recordings were performed at room temperature (22–24°C). Data from whole-cell recordings with a series resistance >20 MΩ were discarded. Passive membrane leak currents present in voltage-ramp recordings were linearly subtracted post-hoc from rod *I*_Ca_ using OriginLab (Northampton, Massachusetts, USA).

### Electroretinography

ERG recordings were obtained using the Espion E^3^ system (Diagnosys LLC, Lowell, MA) as described previously (Liu et al., 2013). Following overnight dark adaptation, males (5–8 week-old) were anesthetized with a ketamine/xylazine mixture (100 mg/kg, i.p.). The pupils were dilated by applying a drop of 1% tropicamide, followed by a drop of 2.5% phenylephrine hydrochloride. ERGs were recorded simultaneously from the corneal surface of each eye using gold ring electrodes (Diagnosys), with an electrode placed on the back of the head as reference and another electrode placed near the tail as ground. A drop of Hypromellose 2.5% Ophthalmic Demulcent Solution (# 17478-0064-12, Akorn Gonak) was placed on the corneal surface to ensure electrical contact and to prevent eyes from drying and cataract formation. Body temperature of mice was maintained at 37°C using the system's heating pad. Mice were placed in a Ganzfeld stimulator chamber (ColorDome; Diagnosys) for delivery of stimuli, and the mouse head and electrode positioning were monitored on the camera attached to the system. ERG responses were evoked by a series of flashes ranging from 0.0001 to 100 cd∙s/m^2^. Responses to six sweeps were averaged for dim flashes up to 0.6 cd∙s/m^2^, two sweeps were averaged for 4 cd∙s/m^2^, and responses to brighter flashes were recorded without averaging. Intersweep intervals for flashes with increasing strength were increased from 10 s to 60 s to allow full recovery from preceding flashes. To record photopic ERGs, mice were exposed to a background light (30 cd•s /m^2^) for 3 min before flash stimulation (3, 30 or 100 cd•s/m^2^); six sweeps were averaged for further analysis.

### Serial block-face scanning electron microscopy and 3D reconstructions

Eye cups were prepared from P42 WT and G369i KI littermates and fixed using 4% glutaraldehyde in 0.1M cacodylate buffer, pH 7.4, for 4 hr at room temperature followed by additional fixation overnight at 4°C. Glutaraldehyde-fixed eye cups were then washed 3 times in 0.1M cacodylate buffer. Retinas were thereafter isolated and embedded in Durcupan resin after staining, dehydration, and embedding as described previously (Della Santina et al., 2016). A Thermo Scientific VolumeScope serial block-face scanning electron microscope was used to image embedded retinas. Retinal regions comprising a 2 × 2 montage of 40.96 μm tiles were imaged at a resolution of 5 nm/pixel and section thickness of 50 nm. Image stacks were aligned, and rod photoreceptor terminals reconstructed using TrakEM2 (NIH). Postsynaptic partners at the rod triad were followed to the INL to determine their identity (HC vs bipolar cell). Amira (Thermo Fisher Scientific) software was used for 3D visualization of reconstructed profiles.

### Quantification, statistical analysis, and experimental design

Immunofluorescent objects were detected within in z-stacks of 30 optical sections and quantified with ObjectFinder software (https://lucadellasantina.github.io/ObjectFinder/) using unbiased iterative threshold search algorithms. To measure rod ribbon length, sections were double-labeled with antibodies against CtBP2 and cone arrestin as described above. 3D binary masks of cone arrestin labeling were generated in order to exclude CtBP2 labeling within cones from analyses. The length of each rod ribbon was measured along the major axis. The sphericity of each detected ribbon was also calculated. Ribbons detected along the edges of the cone arrestin mask and the outer edges of the 3D images were excluded from analyses. To measure nearest-neighbor distances, ObjectFinder was used to detect and measure the distances from ribeye-labeled ‘home’ objects to the nearest mGluR6-labeled ‘neighbor’ objects.

To measure neurite occupancy of the outer nuclear area (ONL) by HCs, rod bipolar cells (RBCs), and cone bipolar cells (CBCs), sections were labeled with antibodies against calbindin (HCs), PKCα (RBCs), and secretagogin (CBCs). Confocal z-stacks were collapsed and labeling was converted to binary masks. Particle analysis (ImageJ) of labeling within the ONL area 12 µm above the OPL/INL border was performed, and occupancy was calculated as a function of the total area analyzed. Images used for analyses were collected from central retina from at least six animals/genotype.

Sample sizes were estimated based on those used previously to identify statistically significant differences in similar datasets and utilizing similar biological replicates (i.e. animals, transfections). All data presented result from at least three independent experiments. Data presented on graphs displayed as symbols and bars represent mean ± SEM. Statistical analyses were performed using GraphPad Prism (v 8.0) and a *p* value of < 0.05 was considered significant. Outliers were identified using ROUT method (Q = 1%) using GraphPad Prism and were excluded from analyses. The specific *n* for each experiment as well as the post-hoc test and corrected *p* values, can be found in the figure legends.

## Data Availability

All data generated or analysed during this study are included in the manuscript and supporting files.

## Additional files

Transparent reporting form
