# Supplementary figures and images for: A dual role for Cav1.4 Ca2+ channels in the molecular and structural organization of the rod photoreceptor synapse
Source: eLife. 2020 Sep 17;9:e62184. doi: 10.7554/eLife.62184 (PMC7561352; doi:10.7554/eLife.62184)

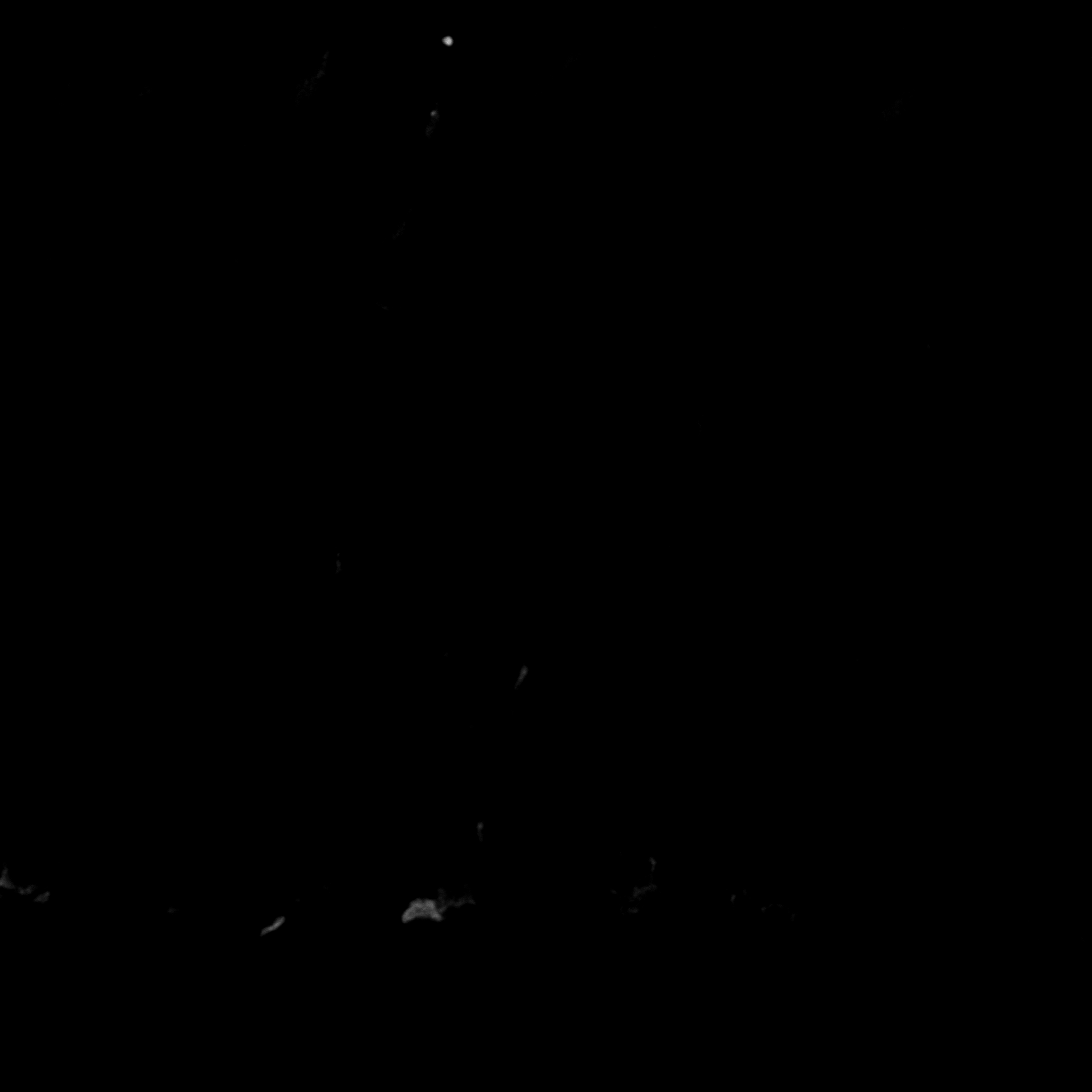

Supplement: Figure 2—source data 1. — The data were exported as 8-bit ‘tif’ files (1600 × 1600 pixels). Values obtained for individual data points (and outlier analysis) for summary graphs in Figure 2B–D are contained in ‘.xlsx’ files. [file elife-62184-fig2-data1.zip › Figure 2- Source Data 1/rod ribbon length images/KI1/P42.KI1.CAR.tif]

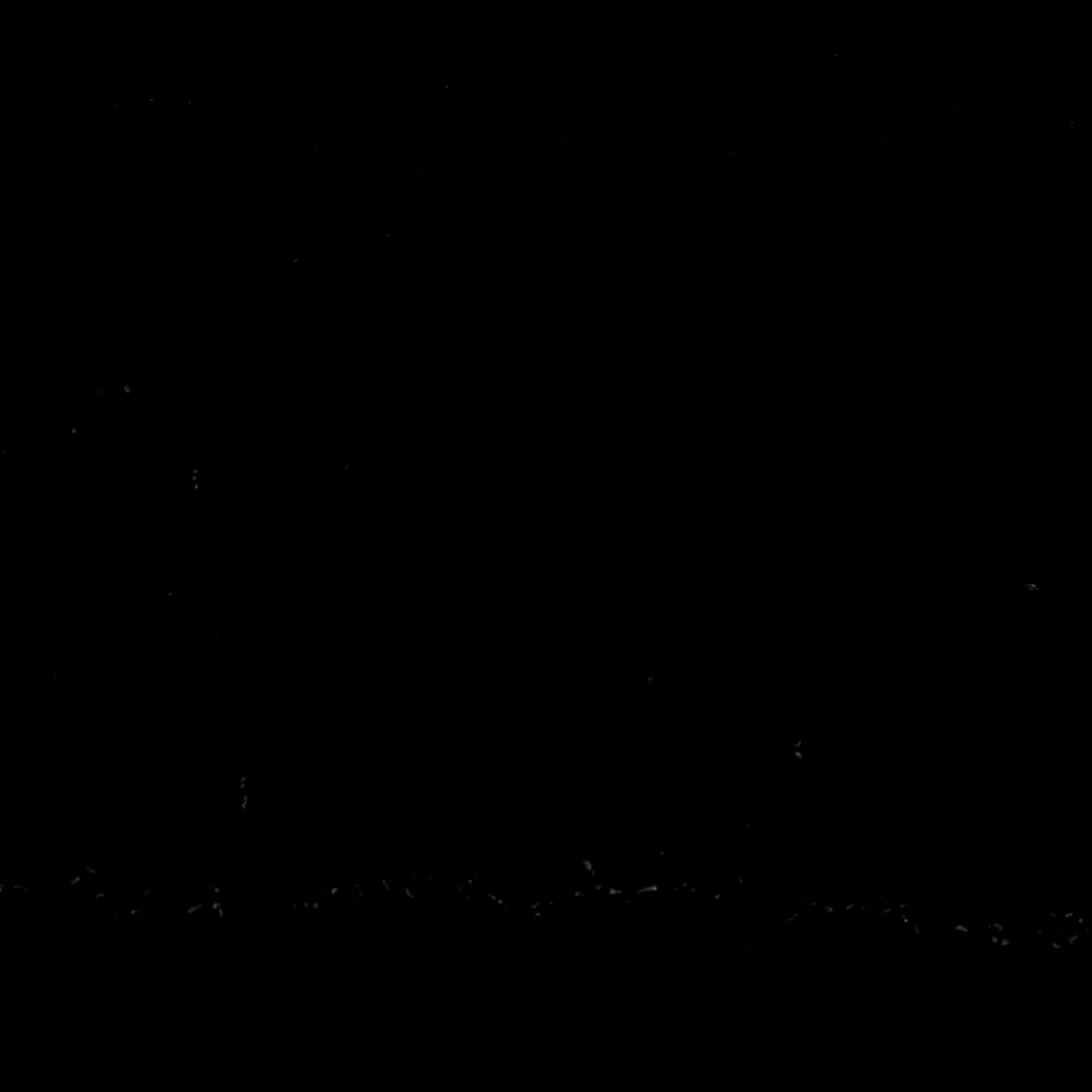

Supplement: Figure 2—source data 1. — The data were exported as 8-bit ‘tif’ files (1600 × 1600 pixels). Values obtained for individual data points (and outlier analysis) for summary graphs in Figure 2B–D are contained in ‘.xlsx’ files. [file elife-62184-fig2-data1.zip › Figure 2- Source Data 1/rod ribbon length images/KI1/P42.KI1.ctbp2.tif]

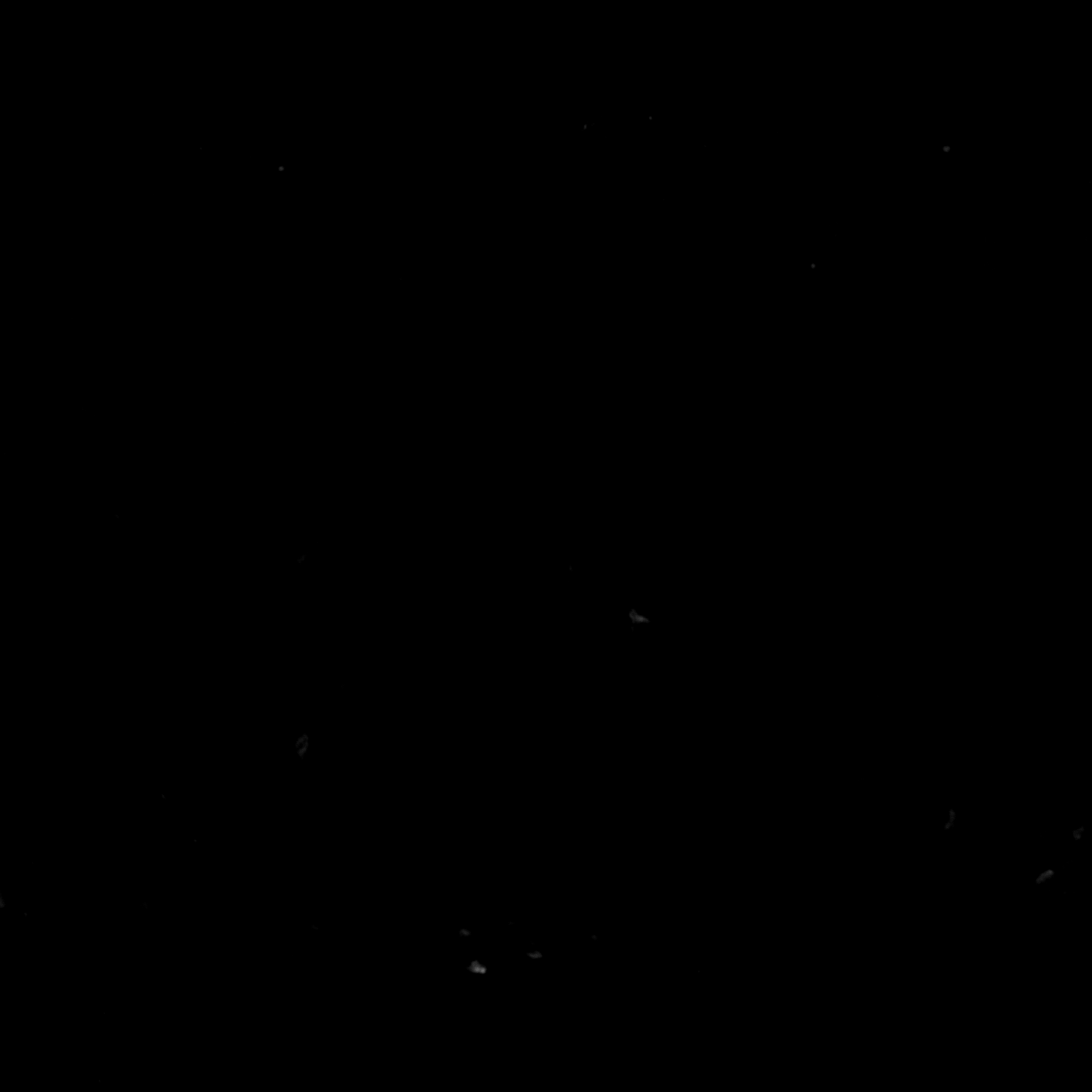

Supplement: Figure 2—source data 1. — The data were exported as 8-bit ‘tif’ files (1600 × 1600 pixels). Values obtained for individual data points (and outlier analysis) for summary graphs in Figure 2B–D are contained in ‘.xlsx’ files. [file elife-62184-fig2-data1.zip › Figure 2- Source Data 1/rod ribbon length images/KO1/P42.KO1.CAR.tif]

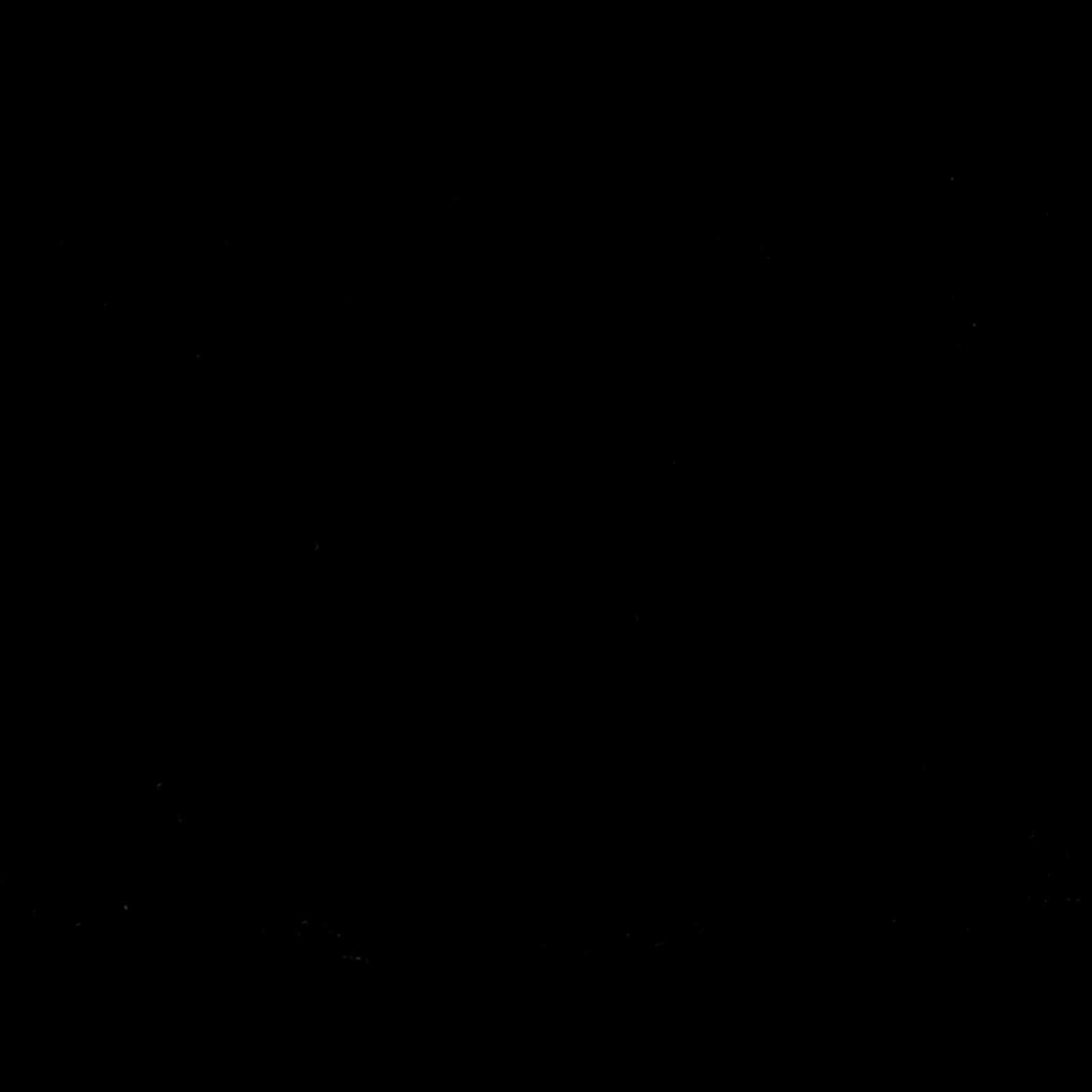

Supplement: Figure 2—source data 1. — The data were exported as 8-bit ‘tif’ files (1600 × 1600 pixels). Values obtained for individual data points (and outlier analysis) for summary graphs in Figure 2B–D are contained in ‘.xlsx’ files. [file elife-62184-fig2-data1.zip › Figure 2- Source Data 1/rod ribbon length images/KO1/P42.KO1.ctbp2.tif]

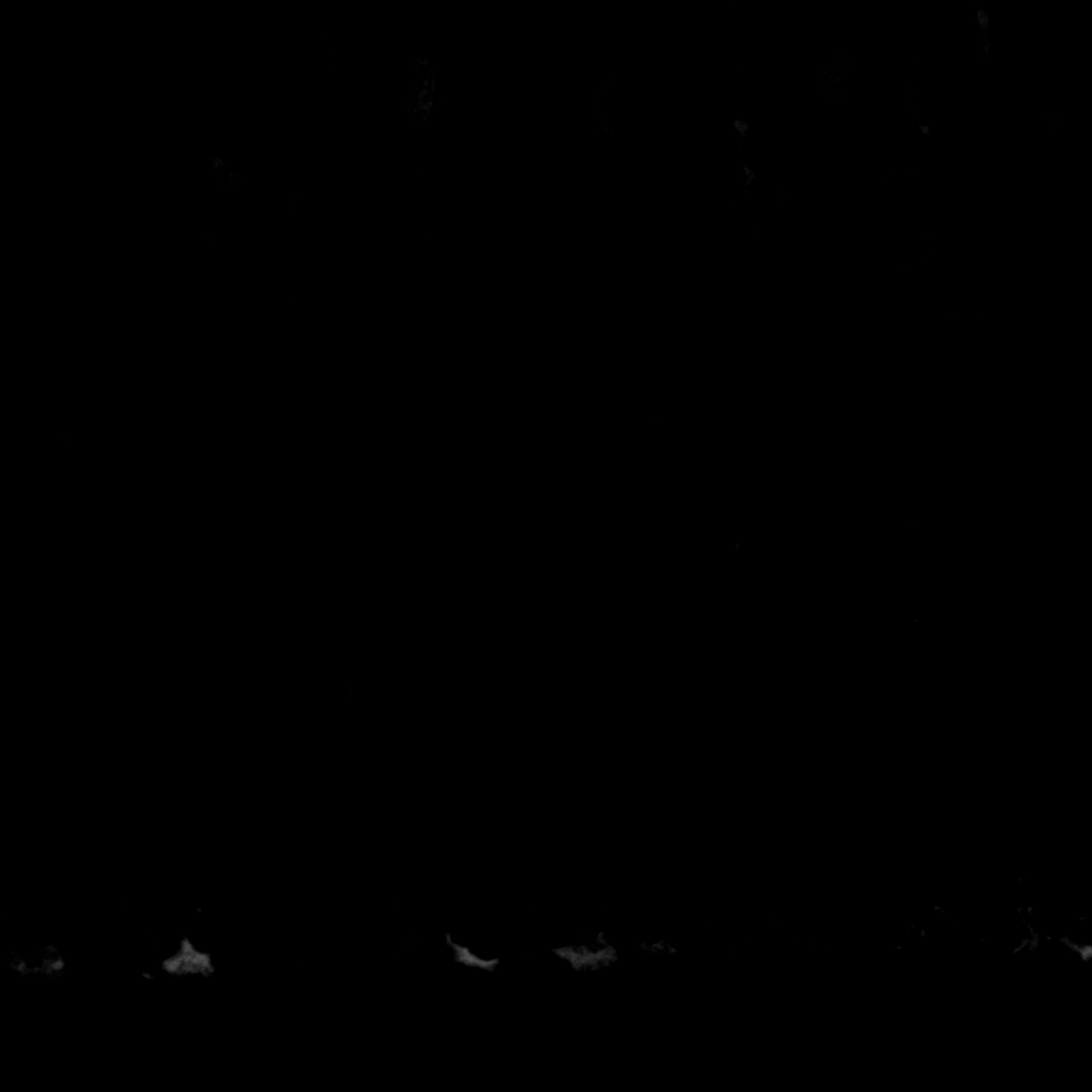

Supplement: Figure 2—source data 1. — The data were exported as 8-bit ‘tif’ files (1600 × 1600 pixels). Values obtained for individual data points (and outlier analysis) for summary graphs in Figure 2B–D are contained in ‘.xlsx’ files. [file elife-62184-fig2-data1.zip › Figure 2- Source Data 1/rod ribbon length images/WT2/P42.WT2.CAR.tif]

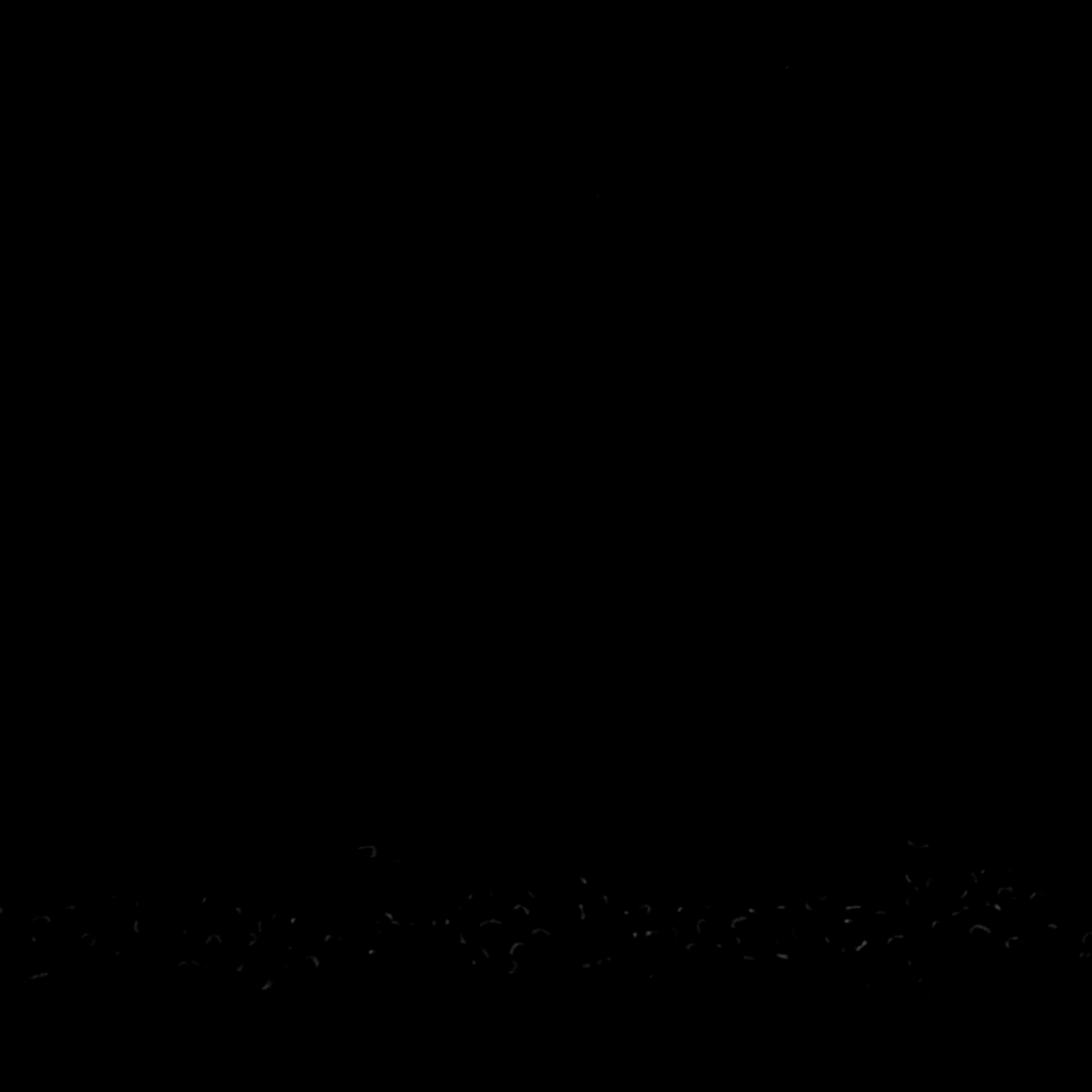

Supplement: Figure 2—source data 1. — The data were exported as 8-bit ‘tif’ files (1600 × 1600 pixels). Values obtained for individual data points (and outlier analysis) for summary graphs in Figure 2B–D are contained in ‘.xlsx’ files. [file elife-62184-fig2-data1.zip › Figure 2- Source Data 1/rod ribbon length images/WT2/P42.WT2.ctbp2.tif]

# CPPG Puffs (WT)

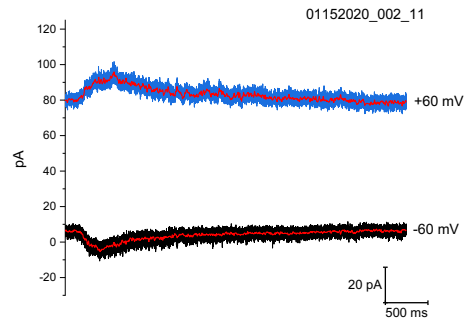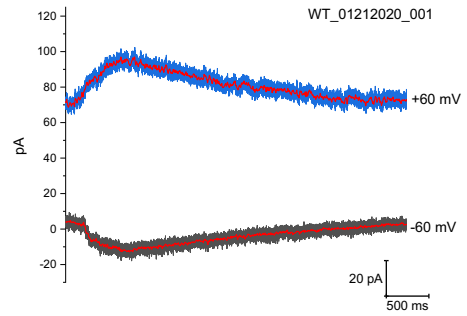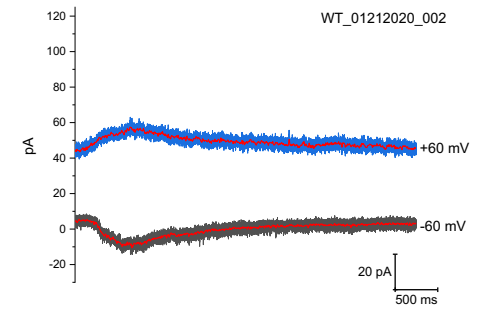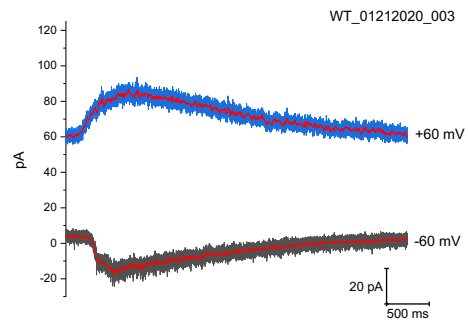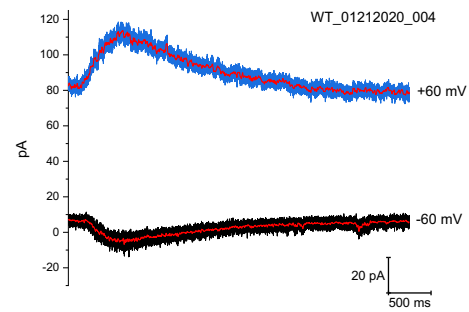

# CPPG Puffs (G369i KI)

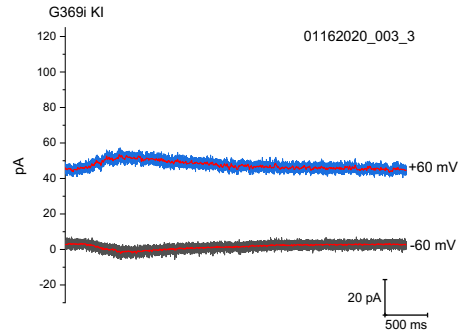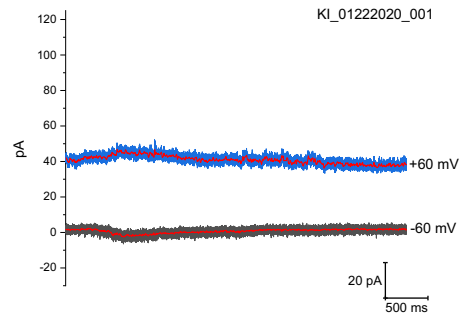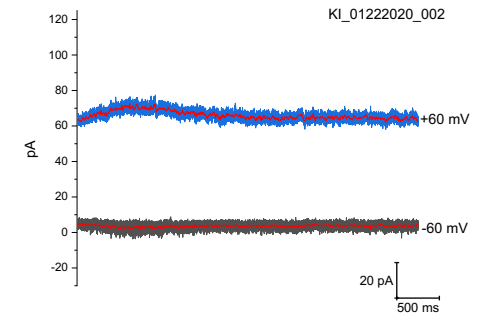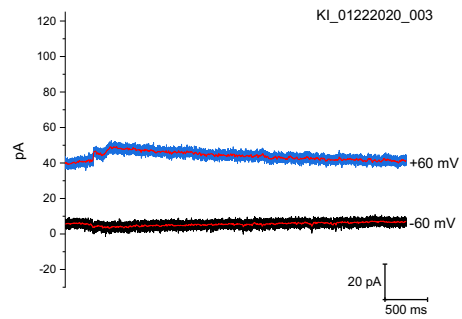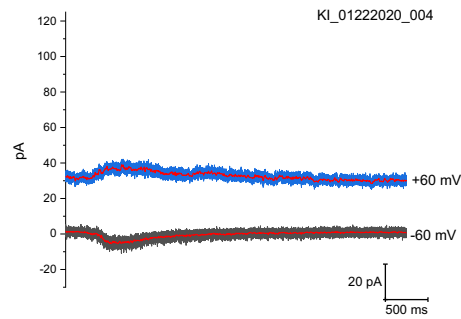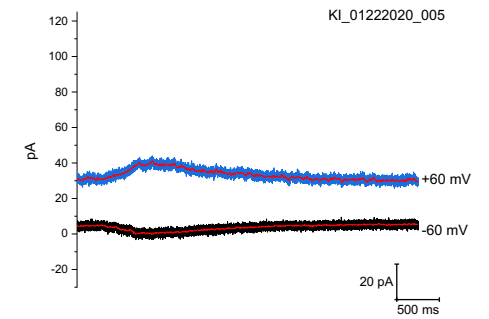

# CPPG Puffs (Ca<sub>v</sub>1.4 KO)

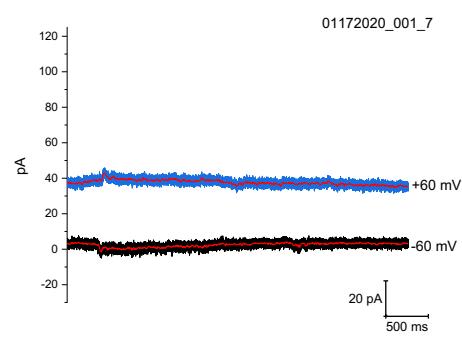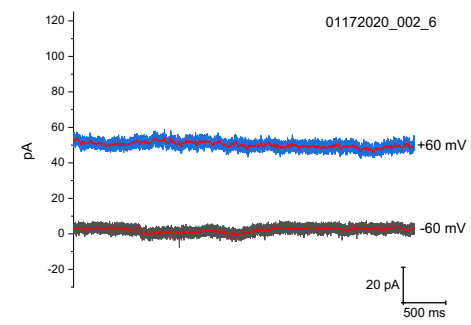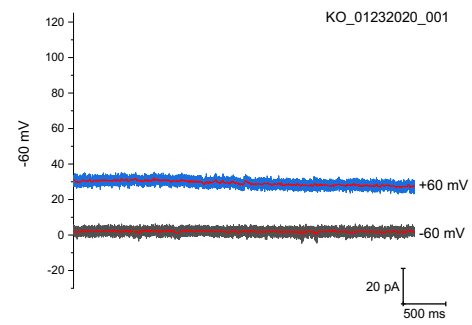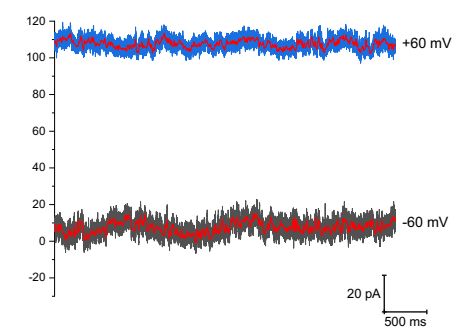

Supplement: Figure 3—source data 1. [file elife-62184-fig3-data1.zip › Figure 3-Source Data1/Figure 3F.pdf]

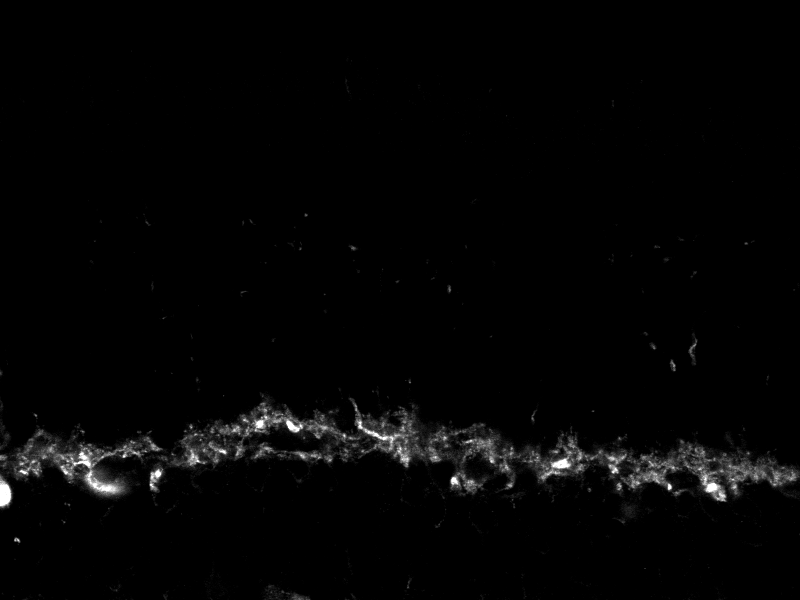

Supplement: Figure 4—source data 1. — The data were exported as 8-bit ‘tif’ files (800 × 600 pixels). Values obtained for individual data points (and outlier analysis) for summary graphs in are contained in ‘.xlsx’ files. [file elife-62184-fig4-data1.zip › Figure 4- Source Data 1/KI1/P42.KI1.calbindin.tif]

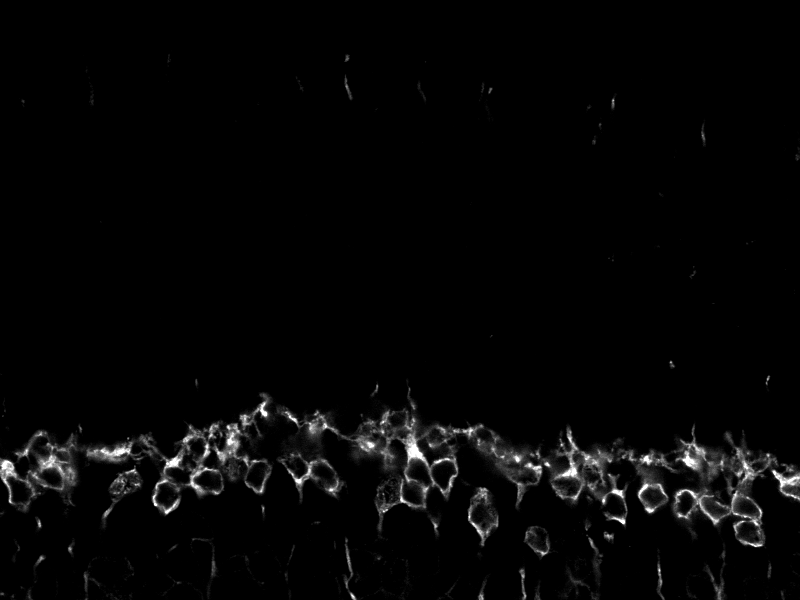

Supplement: Figure 4—source data 1. — The data were exported as 8-bit ‘tif’ files (800 × 600 pixels). Values obtained for individual data points (and outlier analysis) for summary graphs in are contained in ‘.xlsx’ files. [file elife-62184-fig4-data1.zip › Figure 4- Source Data 1/KI1/P42.KI1.PKCa.tif]

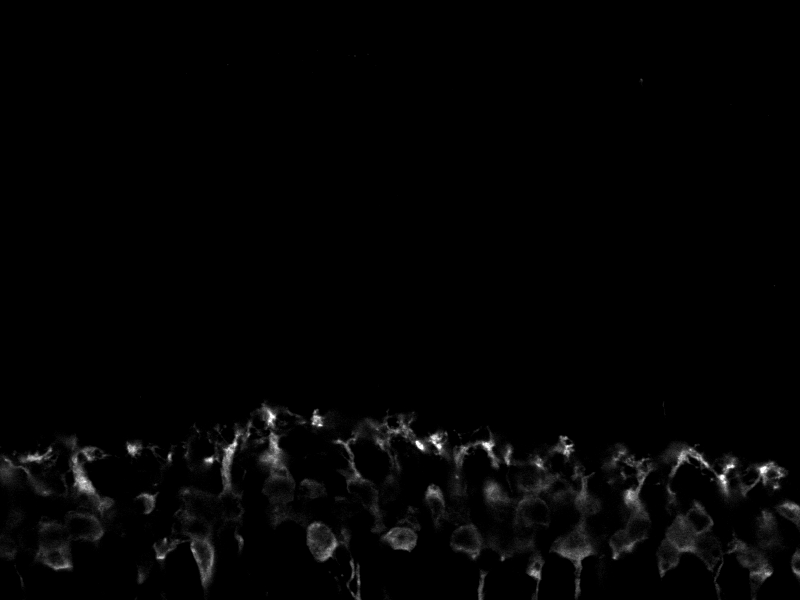

Supplement: Figure 4—source data 1. — The data were exported as 8-bit ‘tif’ files (800 × 600 pixels). Values obtained for individual data points (and outlier analysis) for summary graphs in are contained in ‘.xlsx’ files. [file elife-62184-fig4-data1.zip › Figure 4- Source Data 1/KI1/P42.KI1.SCGN.tif]

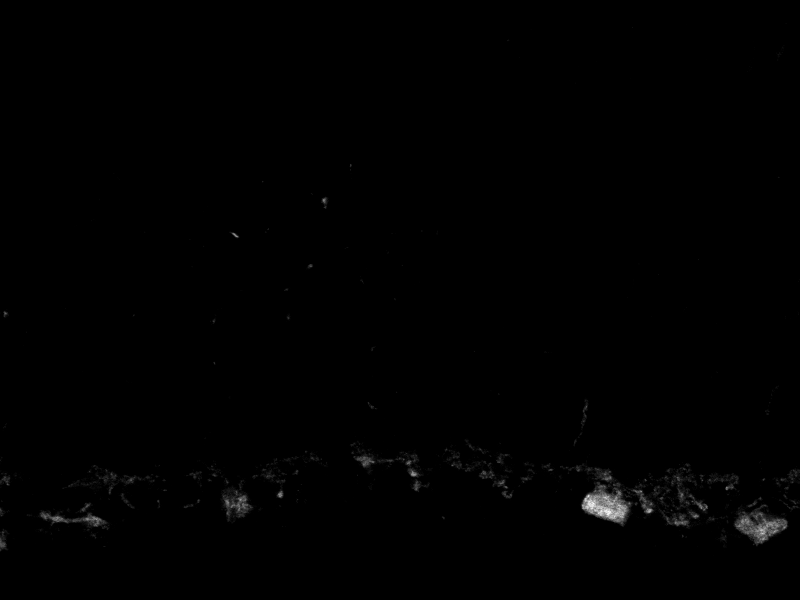

Supplement: Figure 4—source data 1. — The data were exported as 8-bit ‘tif’ files (800 × 600 pixels). Values obtained for individual data points (and outlier analysis) for summary graphs in are contained in ‘.xlsx’ files. [file elife-62184-fig4-data1.zip › Figure 4- Source Data 1/KI2/P42KI2.calbindin.tif]

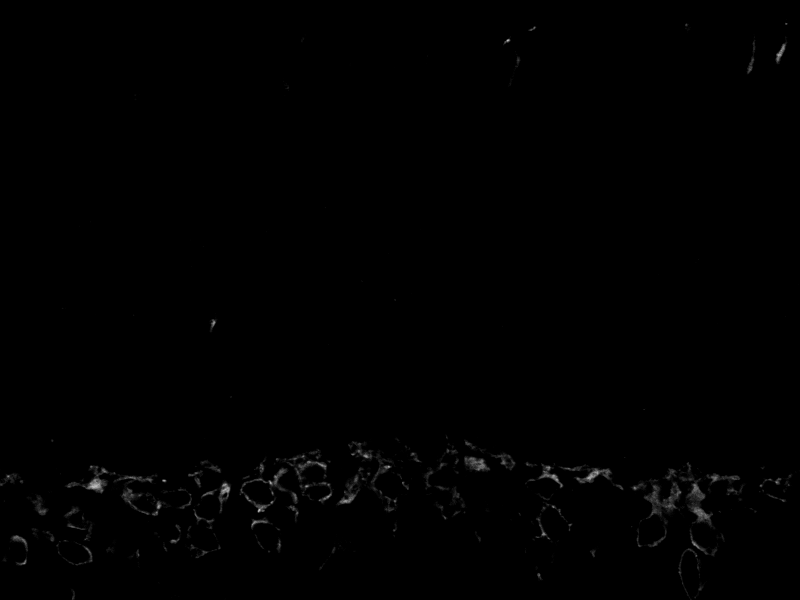

Supplement: Figure 4—source data 1. — The data were exported as 8-bit ‘tif’ files (800 × 600 pixels). Values obtained for individual data points (and outlier analysis) for summary graphs in are contained in ‘.xlsx’ files. [file elife-62184-fig4-data1.zip › Figure 4- Source Data 1/KI2/P42KI2.PKCa.tif]

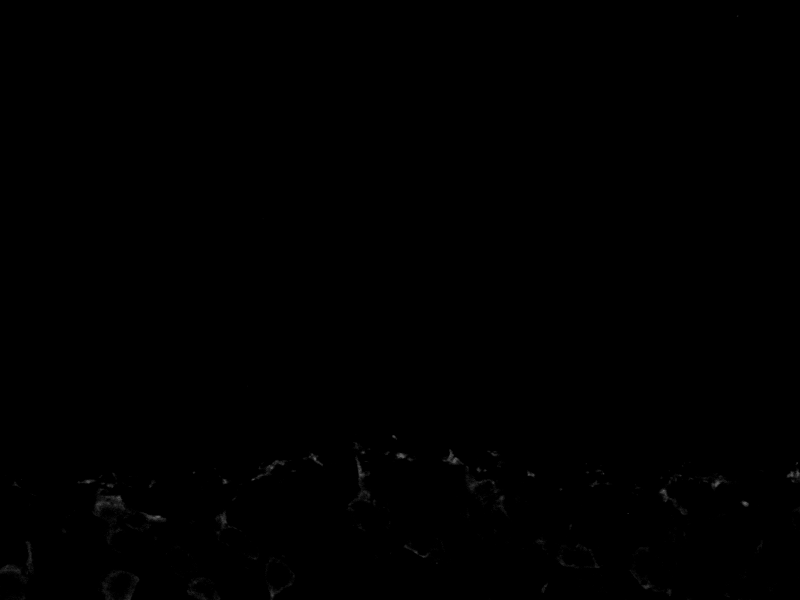

Supplement: Figure 4—source data 1. — The data were exported as 8-bit ‘tif’ files (800 × 600 pixels). Values obtained for individual data points (and outlier analysis) for summary graphs in are contained in ‘.xlsx’ files. [file elife-62184-fig4-data1.zip › Figure 4- Source Data 1/KI2/P42KI2.SCGN.tif]

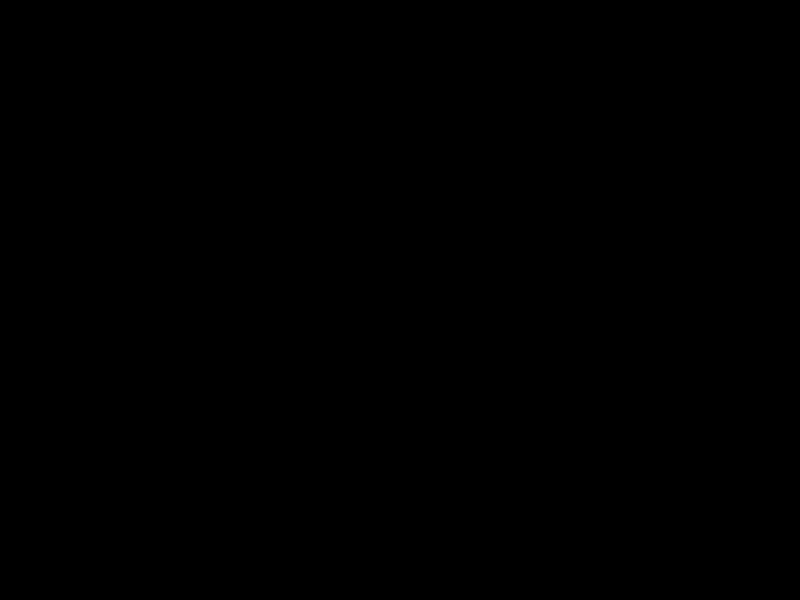

Supplement: Figure 4—source data 1. — The data were exported as 8-bit ‘tif’ files (800 × 600 pixels). Values obtained for individual data points (and outlier analysis) for summary graphs in are contained in ‘.xlsx’ files. [file elife-62184-fig4-data1.zip › Figure 4- Source Data 1/KI3/P42KI3.calbindin.tif]

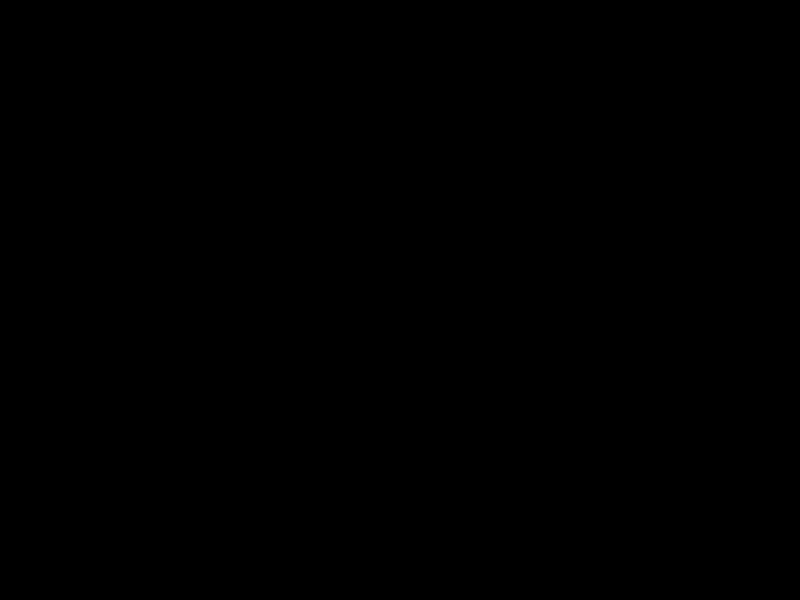

Supplement: Figure 4—source data 1. — The data were exported as 8-bit ‘tif’ files (800 × 600 pixels). Values obtained for individual data points (and outlier analysis) for summary graphs in are contained in ‘.xlsx’ files. [file elife-62184-fig4-data1.zip › Figure 4- Source Data 1/KI3/P42KI3.PKCa.tif]

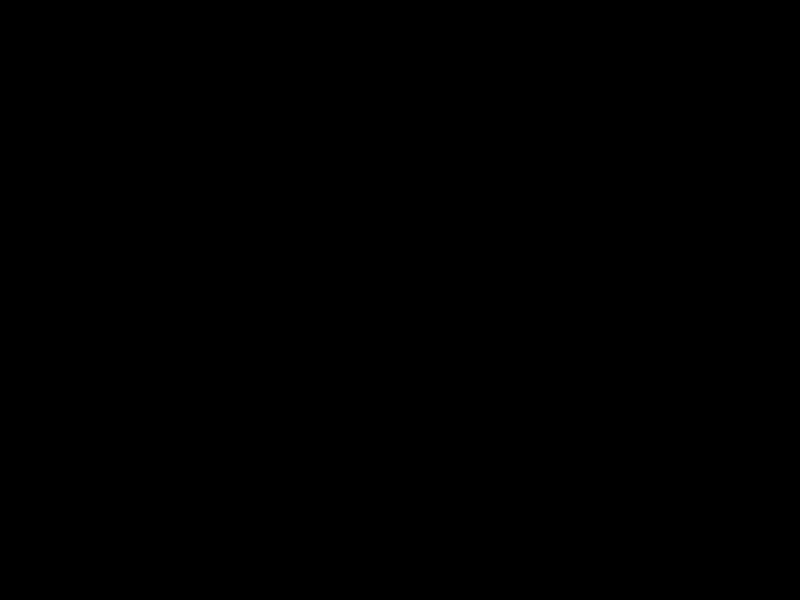

Supplement: Figure 4—source data 1. — The data were exported as 8-bit ‘tif’ files (800 × 600 pixels). Values obtained for individual data points (and outlier analysis) for summary graphs in are contained in ‘.xlsx’ files. [file elife-62184-fig4-data1.zip › Figure 4- Source Data 1/KI3/P42KI3.SCGN.tif]

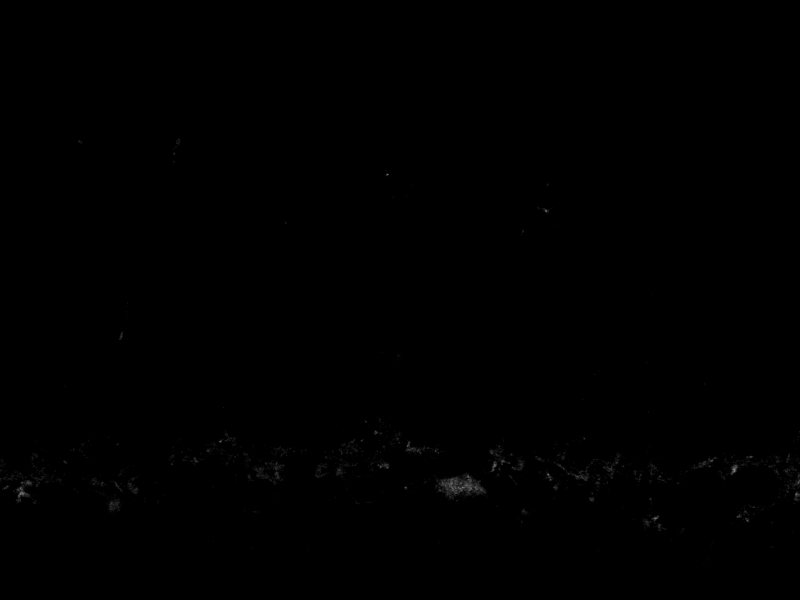

Supplement: Figure 4—source data 1. — The data were exported as 8-bit ‘tif’ files (800 × 600 pixels). Values obtained for individual data points (and outlier analysis) for summary graphs in are contained in ‘.xlsx’ files. [file elife-62184-fig4-data1.zip › Figure 4- Source Data 1/KI4/P42.KI4.calbindin.tif]

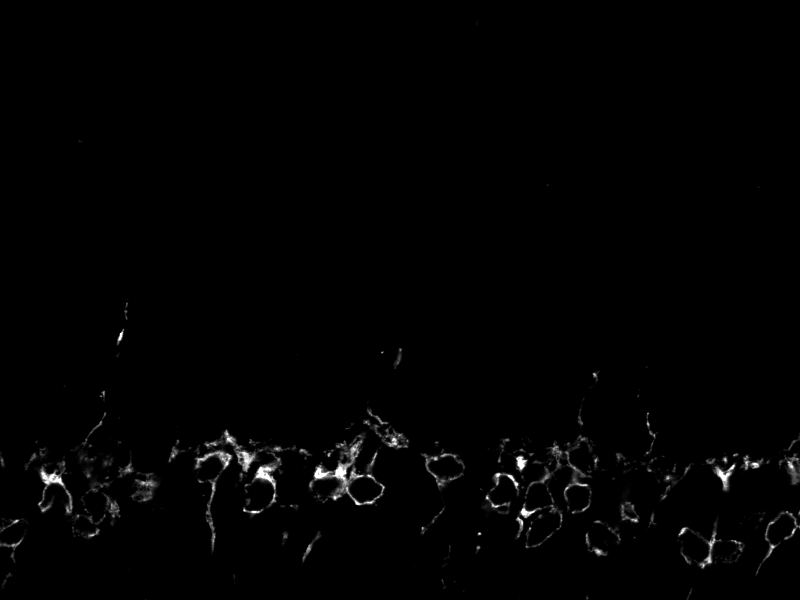

Supplement: Figure 4—source data 1. — The data were exported as 8-bit ‘tif’ files (800 × 600 pixels). Values obtained for individual data points (and outlier analysis) for summary graphs in are contained in ‘.xlsx’ files. [file elife-62184-fig4-data1.zip › Figure 4- Source Data 1/KI4/P42.KI4.PKCa.tif]

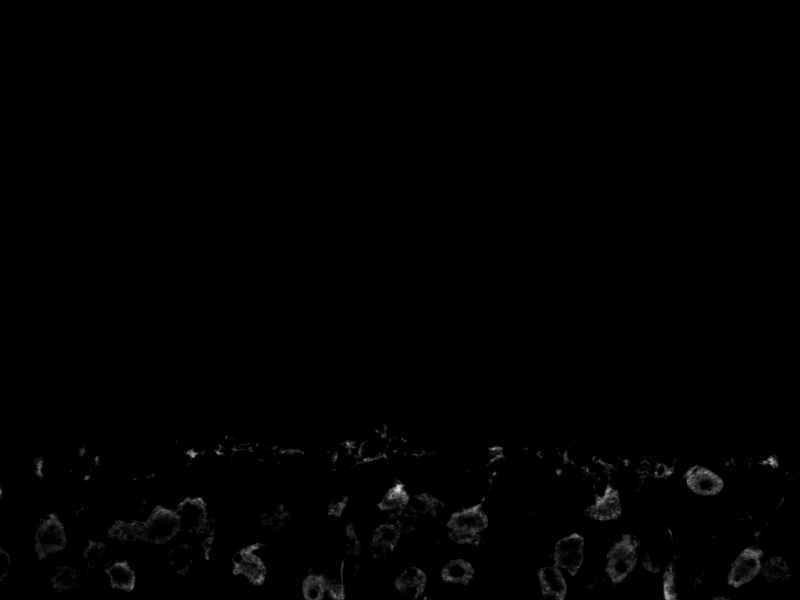

Supplement: Figure 4—source data 1. — The data were exported as 8-bit ‘tif’ files (800 × 600 pixels). Values obtained for individual data points (and outlier analysis) for summary graphs in are contained in ‘.xlsx’ files. [file elife-62184-fig4-data1.zip › Figure 4- Source Data 1/KI4/P42.KI4.SCGN.tif]

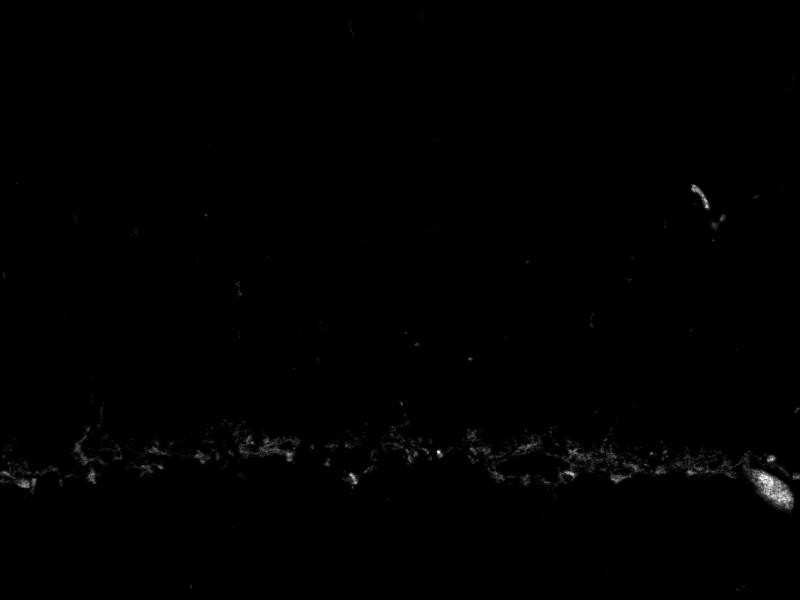

Supplement: Figure 4—source data 1. — The data were exported as 8-bit ‘tif’ files (800 × 600 pixels). Values obtained for individual data points (and outlier analysis) for summary graphs in are contained in ‘.xlsx’ files. [file elife-62184-fig4-data1.zip › Figure 4- Source Data 1/KI5/P42.KI5.calbindin.tif]

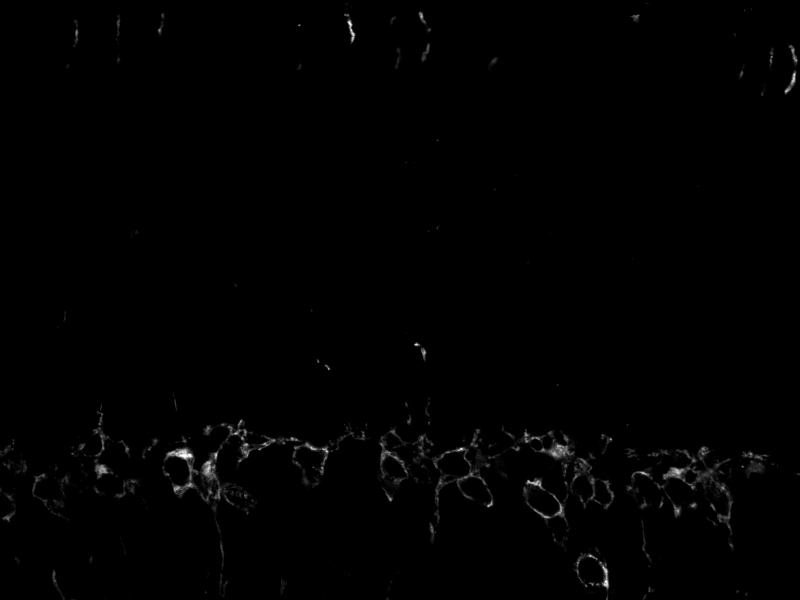

Supplement: Figure 4—source data 1. — The data were exported as 8-bit ‘tif’ files (800 × 600 pixels). Values obtained for individual data points (and outlier analysis) for summary graphs in are contained in ‘.xlsx’ files. [file elife-62184-fig4-data1.zip › Figure 4- Source Data 1/KI5/P42.KI5.PKCa.tif]

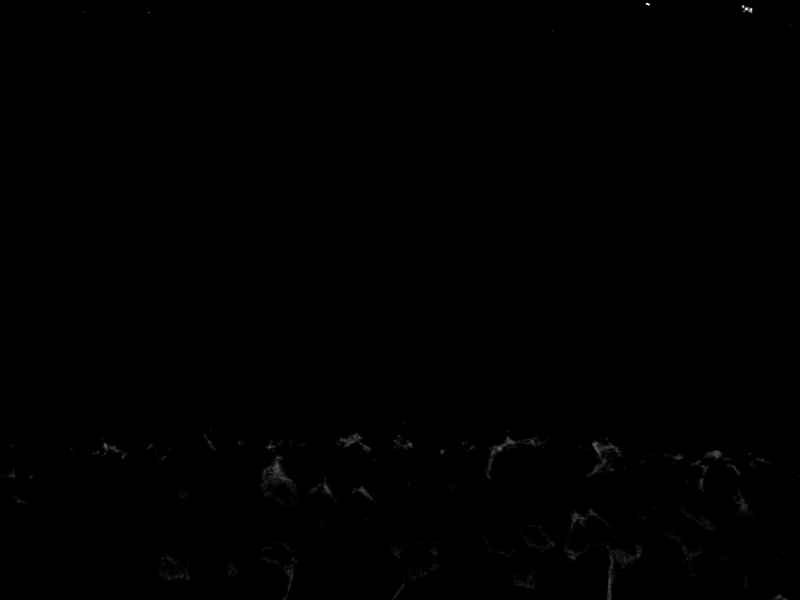

Supplement: Figure 4—source data 1. — The data were exported as 8-bit ‘tif’ files (800 × 600 pixels). Values obtained for individual data points (and outlier analysis) for summary graphs in are contained in ‘.xlsx’ files. [file elife-62184-fig4-data1.zip › Figure 4- Source Data 1/KI5/P42.KI5.SCGN.tif]

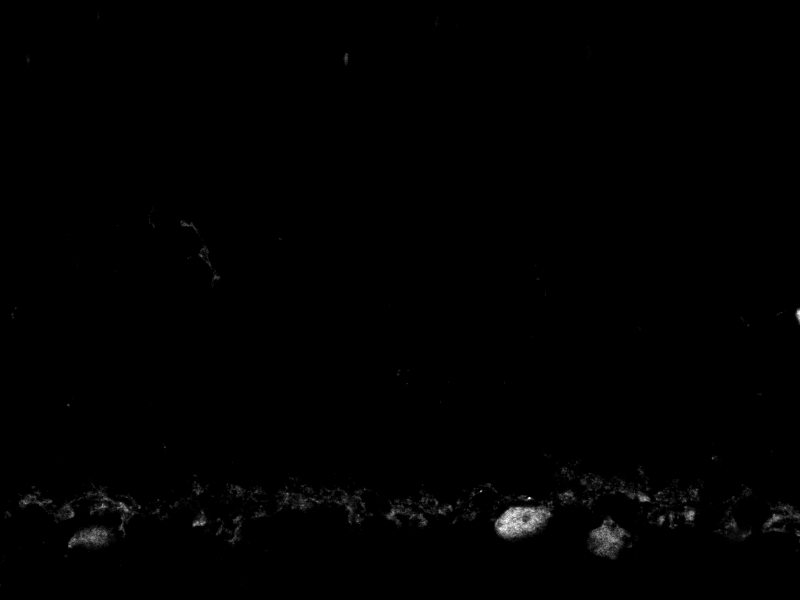

Supplement: Figure 4—source data 1. — The data were exported as 8-bit ‘tif’ files (800 × 600 pixels). Values obtained for individual data points (and outlier analysis) for summary graphs in are contained in ‘.xlsx’ files. [file elife-62184-fig4-data1.zip › Figure 4- Source Data 1/KI6/P42.KI6.calbindin.tif]

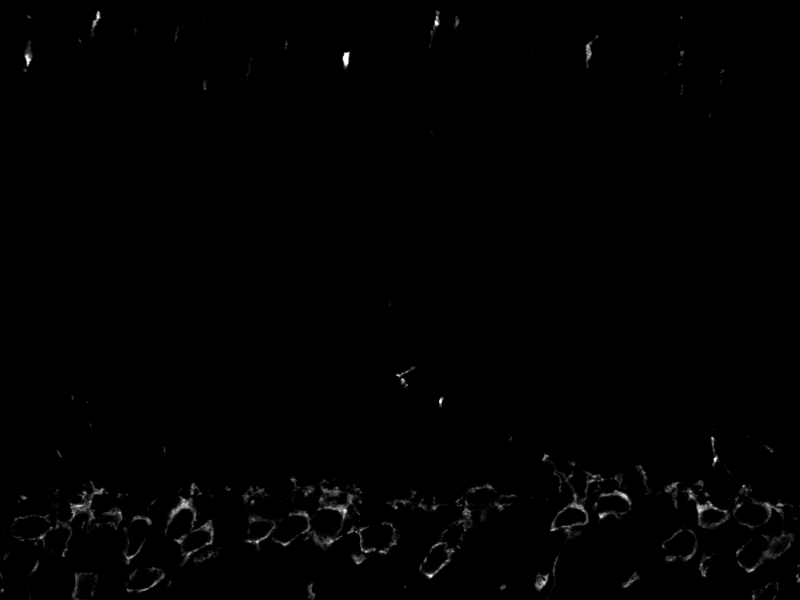

Supplement: Figure 4—source data 1. — The data were exported as 8-bit ‘tif’ files (800 × 600 pixels). Values obtained for individual data points (and outlier analysis) for summary graphs in are contained in ‘.xlsx’ files. [file elife-62184-fig4-data1.zip › Figure 4- Source Data 1/KI6/P42.KI6.PKCa.tif]

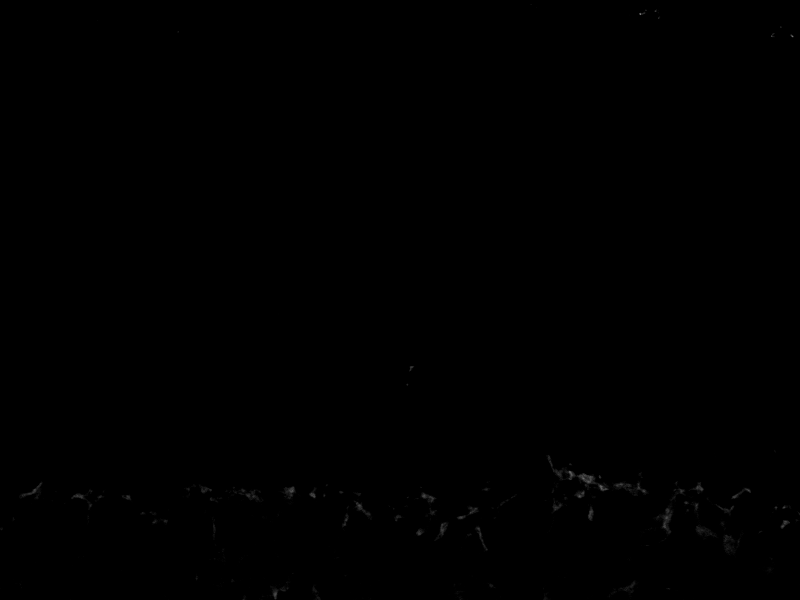

Supplement: Figure 4—source data 1. — The data were exported as 8-bit ‘tif’ files (800 × 600 pixels). Values obtained for individual data points (and outlier analysis) for summary graphs in are contained in ‘.xlsx’ files. [file elife-62184-fig4-data1.zip › Figure 4- Source Data 1/KI6/P42.KI6.SCGN.tif]

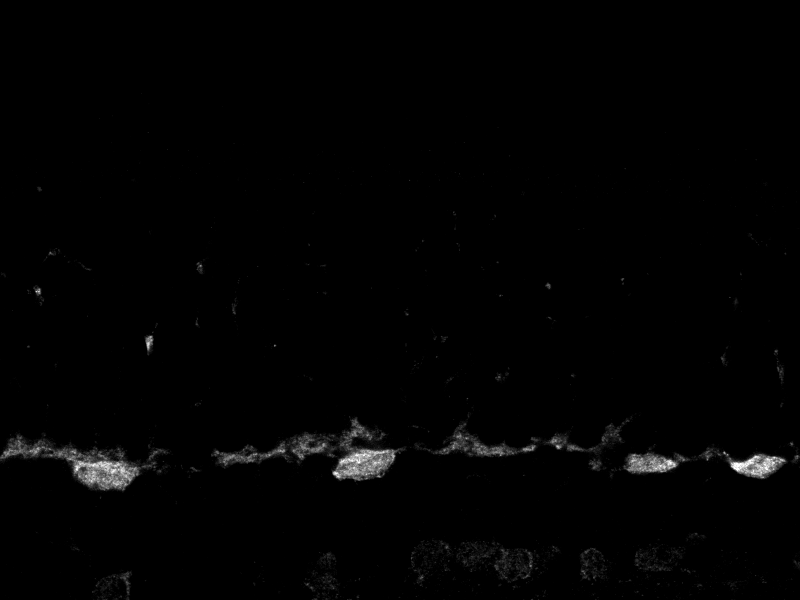

Supplement: Figure 4—source data 1. — The data were exported as 8-bit ‘tif’ files (800 × 600 pixels). Values obtained for individual data points (and outlier analysis) for summary graphs in are contained in ‘.xlsx’ files. [file elife-62184-fig4-data1.zip › Figure 4- Source Data 1/KO1/P42.KO1.calbindin.tif]

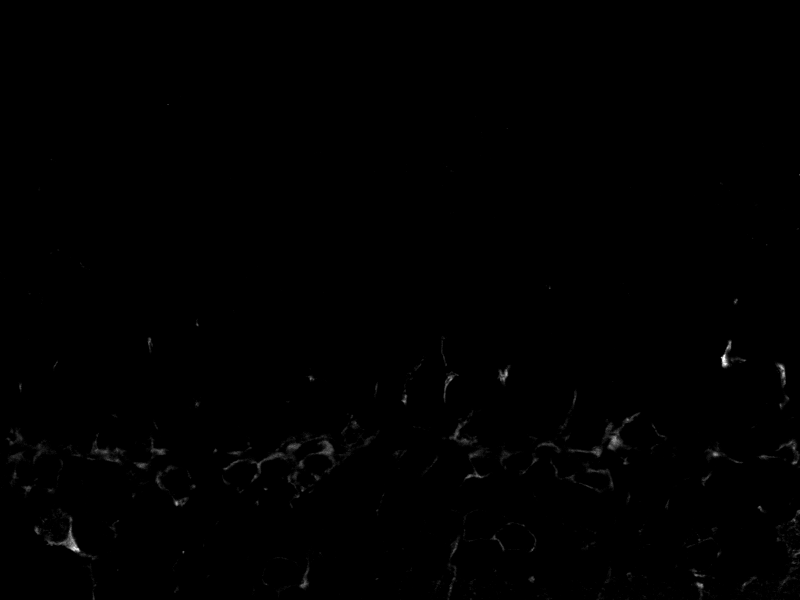

Supplement: Figure 4—source data 1. — The data were exported as 8-bit ‘tif’ files (800 × 600 pixels). Values obtained for individual data points (and outlier analysis) for summary graphs in are contained in ‘.xlsx’ files. [file elife-62184-fig4-data1.zip › Figure 4- Source Data 1/KO1/P42.KO1.PKCa.tif]

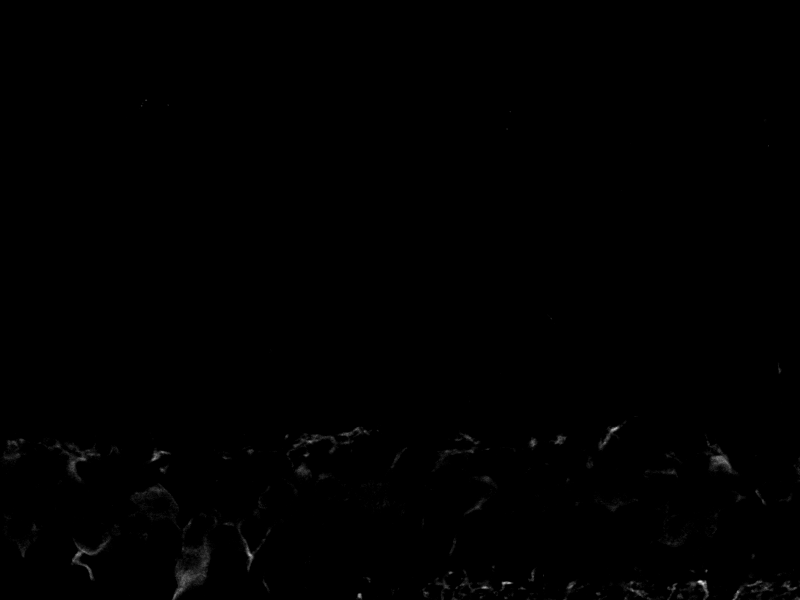

Supplement: Figure 4—source data 1. — The data were exported as 8-bit ‘tif’ files (800 × 600 pixels). Values obtained for individual data points (and outlier analysis) for summary graphs in are contained in ‘.xlsx’ files. [file elife-62184-fig4-data1.zip › Figure 4- Source Data 1/KO1/P42.KO1.SCGN.tif]

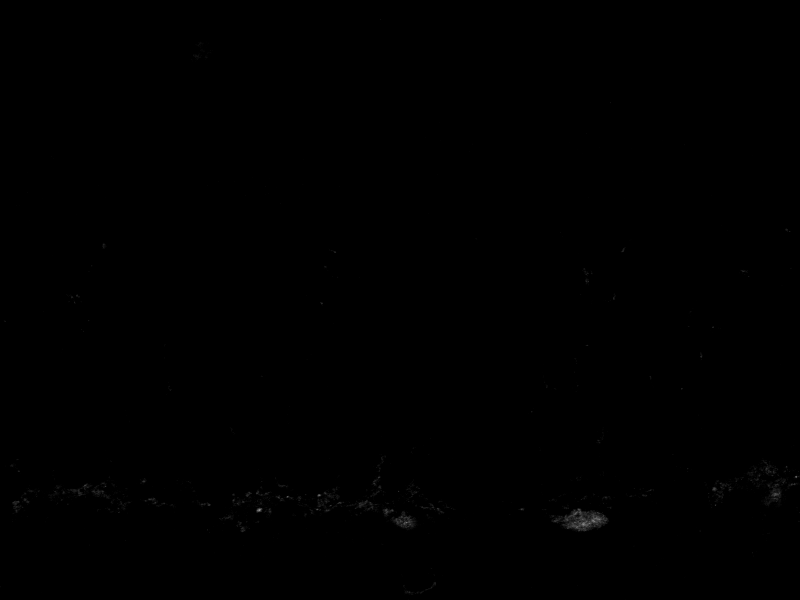

Supplement: Figure 4—source data 1. — The data were exported as 8-bit ‘tif’ files (800 × 600 pixels). Values obtained for individual data points (and outlier analysis) for summary graphs in are contained in ‘.xlsx’ files. [file elife-62184-fig4-data1.zip › Figure 4- Source Data 1/KO2/P42.KO2.calbindin.tif]

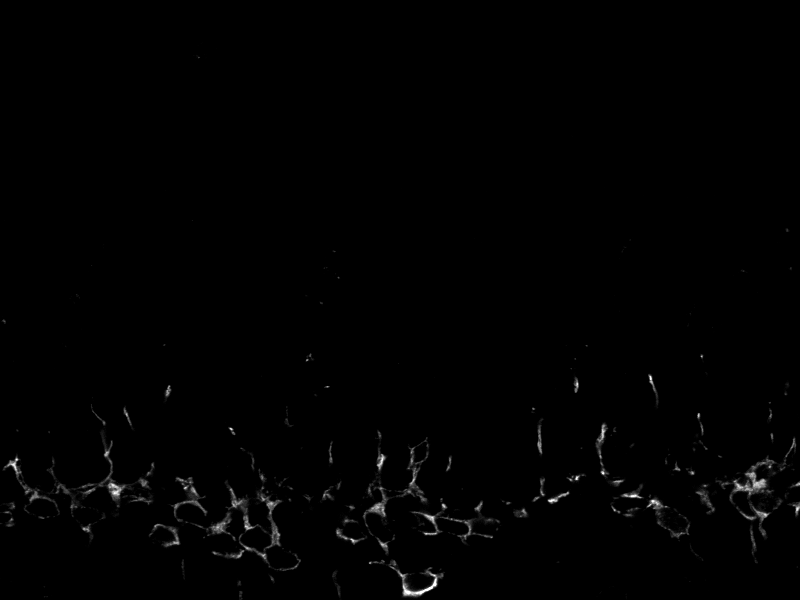

Supplement: Figure 4—source data 1. — The data were exported as 8-bit ‘tif’ files (800 × 600 pixels). Values obtained for individual data points (and outlier analysis) for summary graphs in are contained in ‘.xlsx’ files. [file elife-62184-fig4-data1.zip › Figure 4- Source Data 1/KO2/P42.KO2.PKCa.tif]

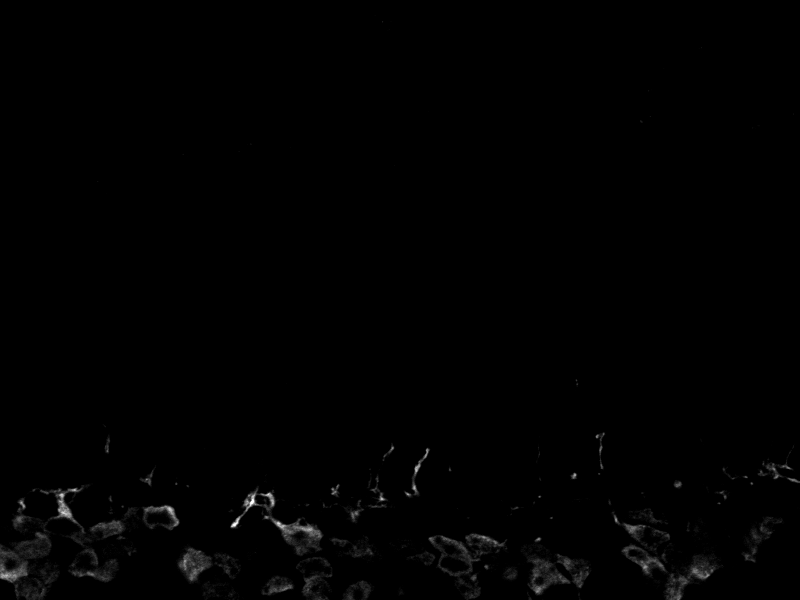

Supplement: Figure 4—source data 1. — The data were exported as 8-bit ‘tif’ files (800 × 600 pixels). Values obtained for individual data points (and outlier analysis) for summary graphs in are contained in ‘.xlsx’ files. [file elife-62184-fig4-data1.zip › Figure 4- Source Data 1/KO2/P42.KO2.SCGN.tif]

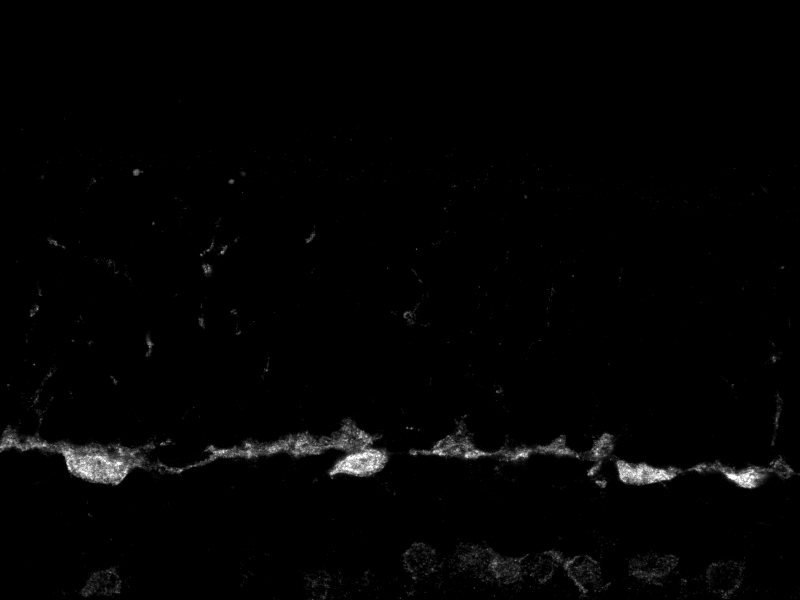

Supplement: Figure 4—source data 1. — The data were exported as 8-bit ‘tif’ files (800 × 600 pixels). Values obtained for individual data points (and outlier analysis) for summary graphs in are contained in ‘.xlsx’ files. [file elife-62184-fig4-data1.zip › Figure 4- Source Data 1/KO3/P42.KO3.calbindin.tif]

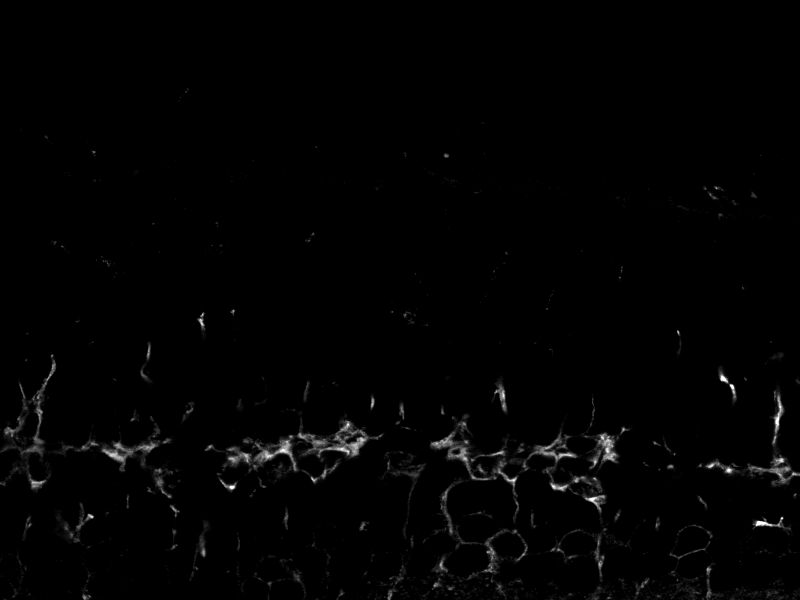

Supplement: Figure 4—source data 1. — The data were exported as 8-bit ‘tif’ files (800 × 600 pixels). Values obtained for individual data points (and outlier analysis) for summary graphs in are contained in ‘.xlsx’ files. [file elife-62184-fig4-data1.zip › Figure 4- Source Data 1/KO3/P42.KO3.PKCa.tif]

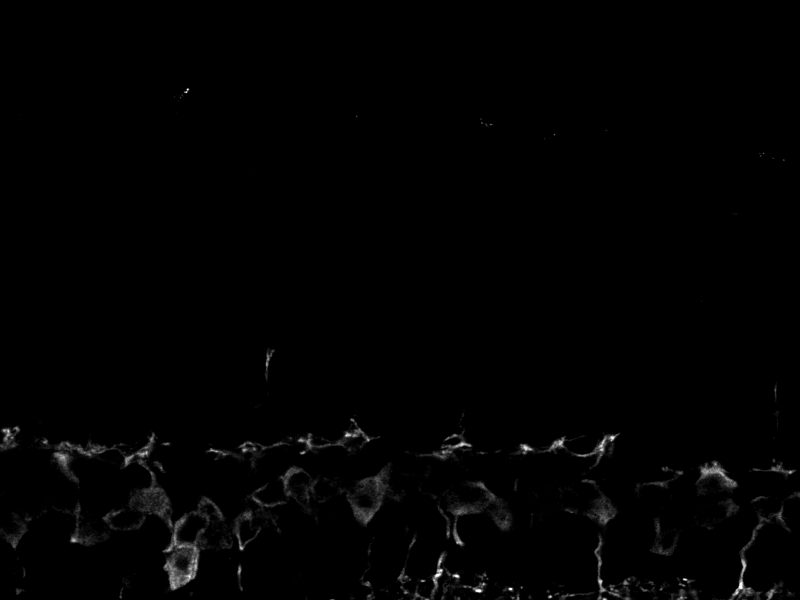

Supplement: Figure 4—source data 1. — The data were exported as 8-bit ‘tif’ files (800 × 600 pixels). Values obtained for individual data points (and outlier analysis) for summary graphs in are contained in ‘.xlsx’ files. [file elife-62184-fig4-data1.zip › Figure 4- Source Data 1/KO3/P42.KO3.SCGN.tif]

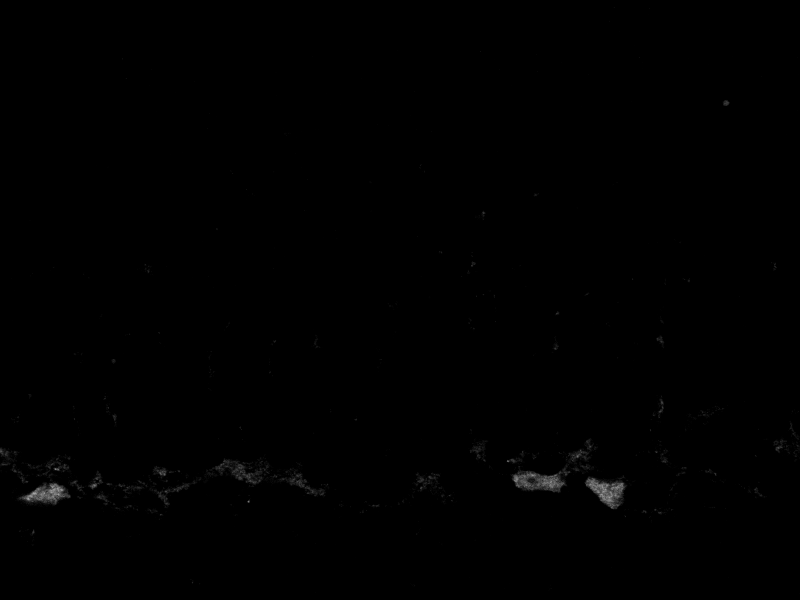

Supplement: Figure 4—source data 1. — The data were exported as 8-bit ‘tif’ files (800 × 600 pixels). Values obtained for individual data points (and outlier analysis) for summary graphs in are contained in ‘.xlsx’ files. [file elife-62184-fig4-data1.zip › Figure 4- Source Data 1/KO4/P42.KO4.calbindin.tif]

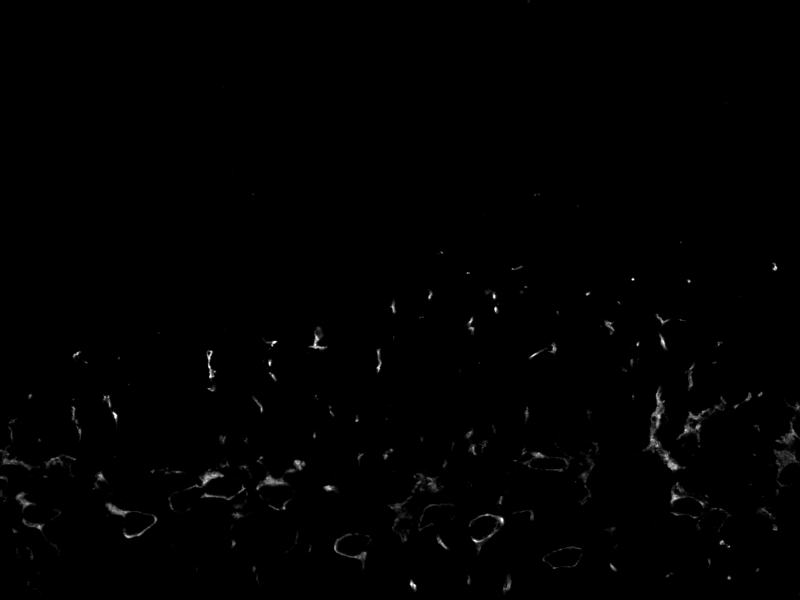

Supplement: Figure 4—source data 1. — The data were exported as 8-bit ‘tif’ files (800 × 600 pixels). Values obtained for individual data points (and outlier analysis) for summary graphs in are contained in ‘.xlsx’ files. [file elife-62184-fig4-data1.zip › Figure 4- Source Data 1/KO4/P42.KO4.PKCa.tif]

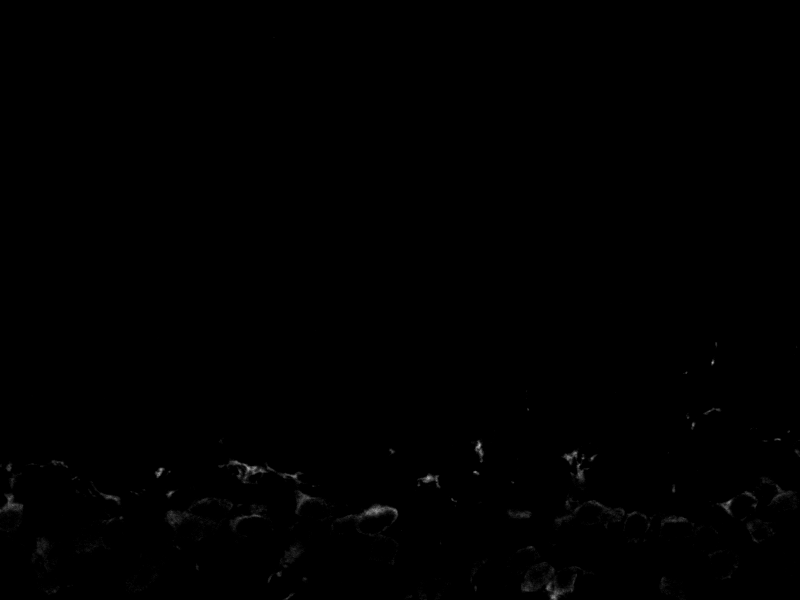

Supplement: Figure 4—source data 1. — The data were exported as 8-bit ‘tif’ files (800 × 600 pixels). Values obtained for individual data points (and outlier analysis) for summary graphs in are contained in ‘.xlsx’ files. [file elife-62184-fig4-data1.zip › Figure 4- Source Data 1/KO4/P42.KO4.SCGN.tif]

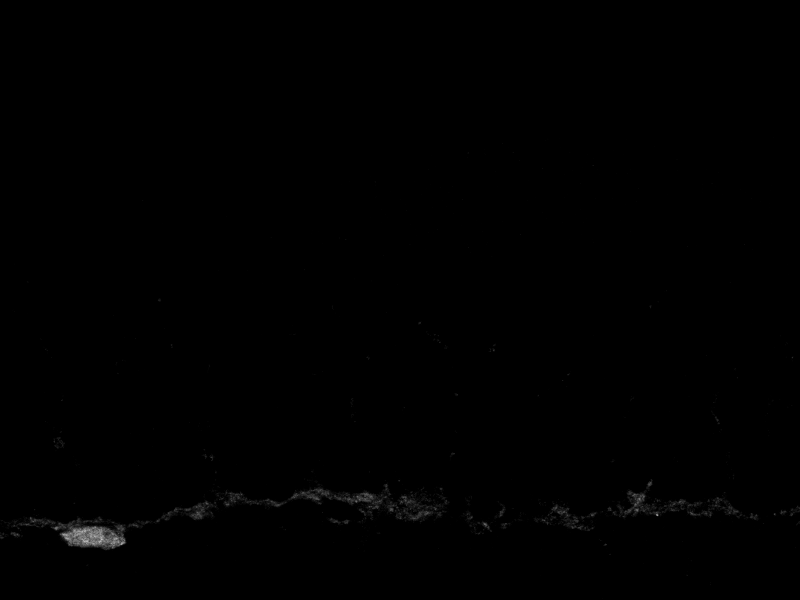

Supplement: Figure 4—source data 1. — The data were exported as 8-bit ‘tif’ files (800 × 600 pixels). Values obtained for individual data points (and outlier analysis) for summary graphs in are contained in ‘.xlsx’ files. [file elife-62184-fig4-data1.zip › Figure 4- Source Data 1/KO5/P42.KO5.calbindin.tif]

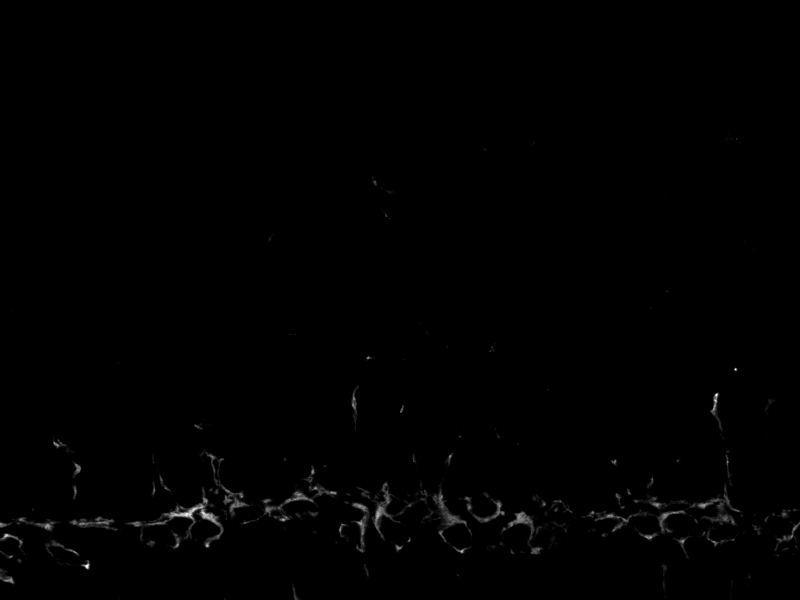

Supplement: Figure 4—source data 1. — The data were exported as 8-bit ‘tif’ files (800 × 600 pixels). Values obtained for individual data points (and outlier analysis) for summary graphs in are contained in ‘.xlsx’ files. [file elife-62184-fig4-data1.zip › Figure 4- Source Data 1/KO5/P42.KO5.PKCa.tif]

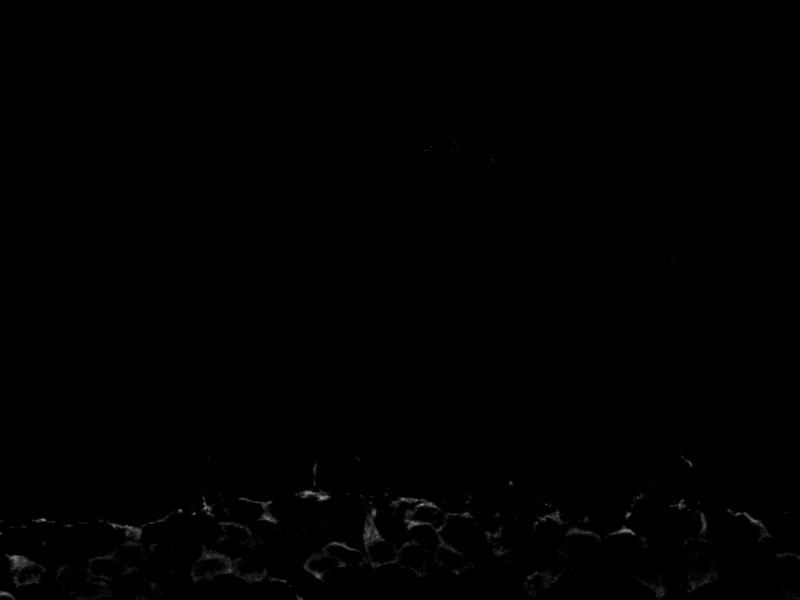

Supplement: Figure 4—source data 1. — The data were exported as 8-bit ‘tif’ files (800 × 600 pixels). Values obtained for individual data points (and outlier analysis) for summary graphs in are contained in ‘.xlsx’ files. [file elife-62184-fig4-data1.zip › Figure 4- Source Data 1/KO5/P42.KO5.SCGN.tif]

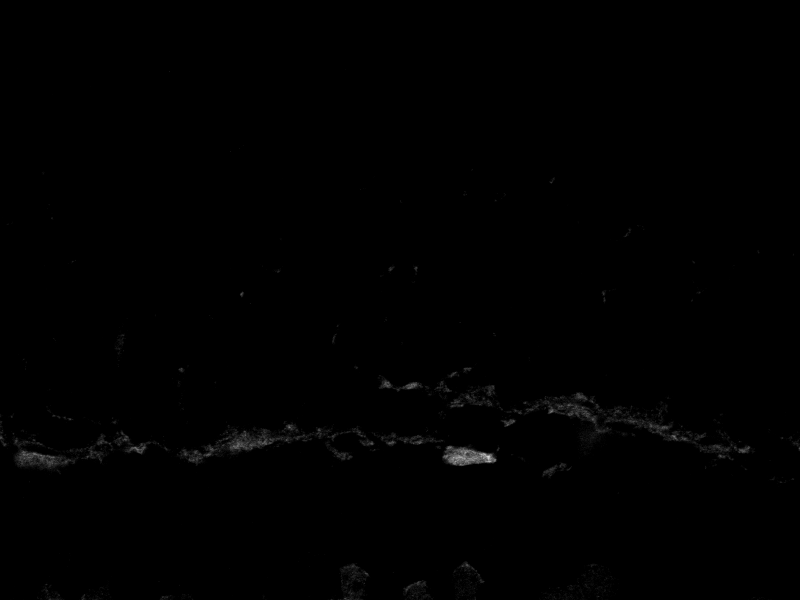

Supplement: Figure 4—source data 1. — The data were exported as 8-bit ‘tif’ files (800 × 600 pixels). Values obtained for individual data points (and outlier analysis) for summary graphs in are contained in ‘.xlsx’ files. [file elife-62184-fig4-data1.zip › Figure 4- Source Data 1/KO6/P42.KO6.calbindin.tif]

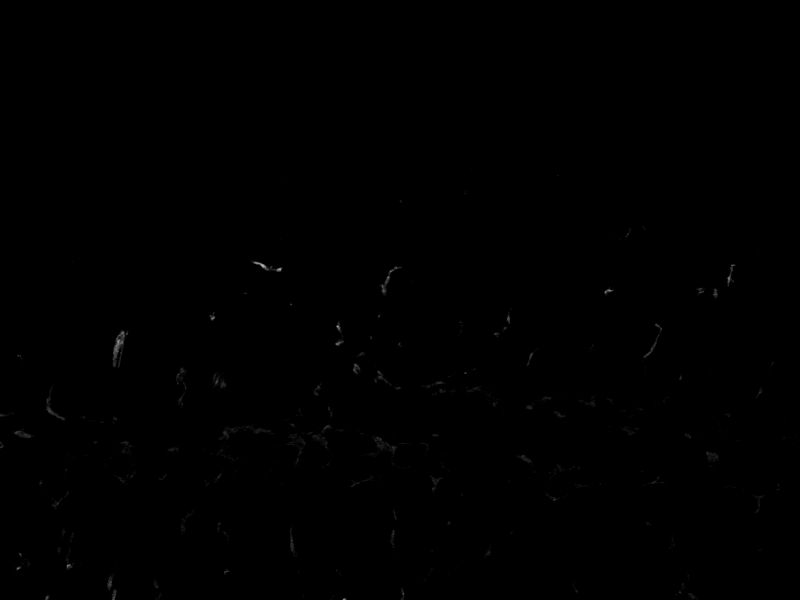

Supplement: Figure 4—source data 1. — The data were exported as 8-bit ‘tif’ files (800 × 600 pixels). Values obtained for individual data points (and outlier analysis) for summary graphs in are contained in ‘.xlsx’ files. [file elife-62184-fig4-data1.zip › Figure 4- Source Data 1/KO6/P42.KO6.PKCa.tif]

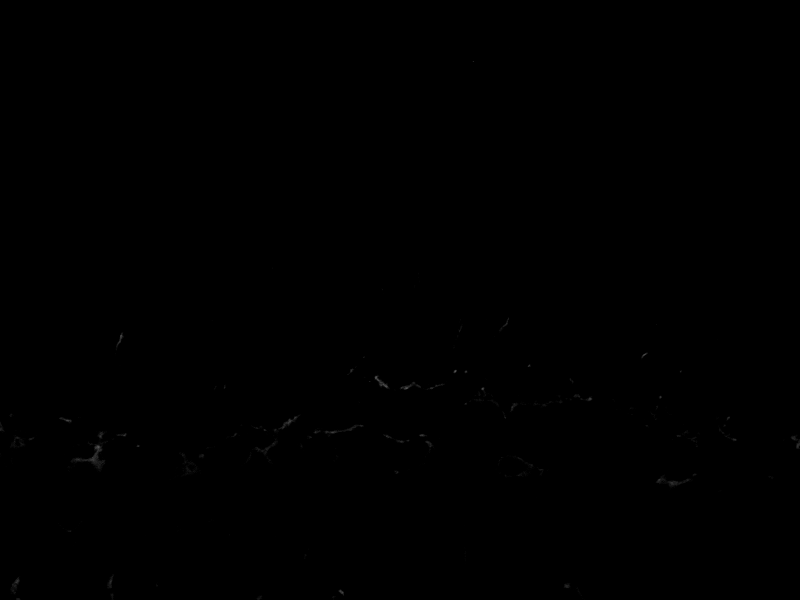

Supplement: Figure 4—source data 1. — The data were exported as 8-bit ‘tif’ files (800 × 600 pixels). Values obtained for individual data points (and outlier analysis) for summary graphs in are contained in ‘.xlsx’ files. [file elife-62184-fig4-data1.zip › Figure 4- Source Data 1/KO6/P42.KO6.SCGN.tif]

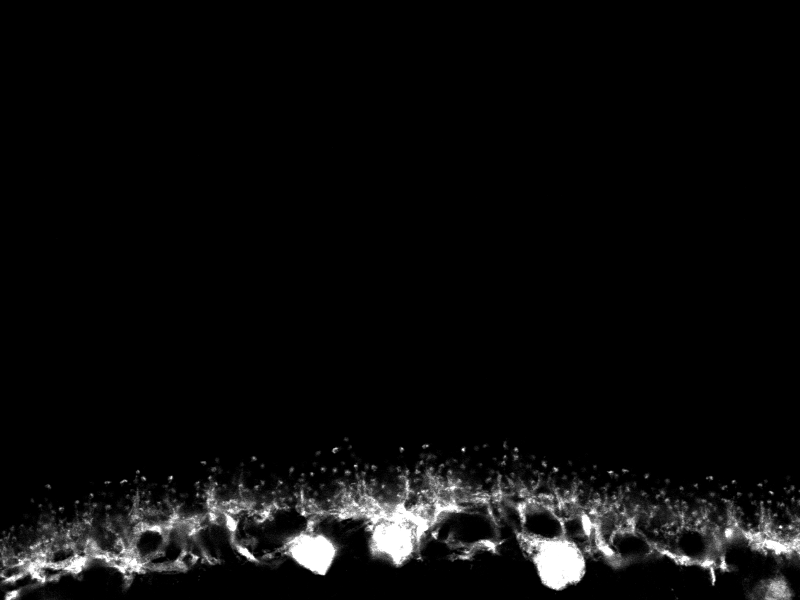

Supplement: Figure 4—source data 1. — The data were exported as 8-bit ‘tif’ files (800 × 600 pixels). Values obtained for individual data points (and outlier analysis) for summary graphs in are contained in ‘.xlsx’ files. [file elife-62184-fig4-data1.zip › Figure 4- Source Data 1/WT1/P42.WT1.calbindin.tif]

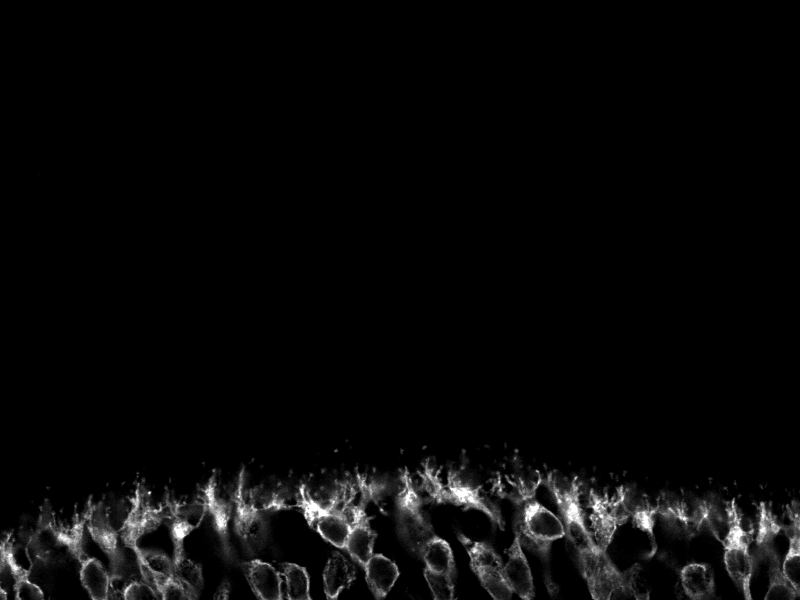

Supplement: Figure 4—source data 1. — The data were exported as 8-bit ‘tif’ files (800 × 600 pixels). Values obtained for individual data points (and outlier analysis) for summary graphs in are contained in ‘.xlsx’ files. [file elife-62184-fig4-data1.zip › Figure 4- Source Data 1/WT1/P42.WT1.PKCa.tif]

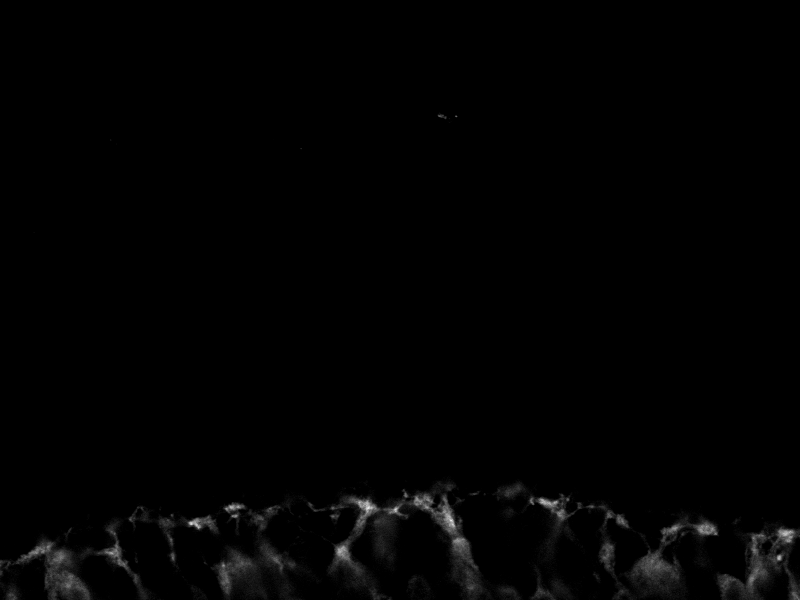

Supplement: Figure 4—source data 1. — The data were exported as 8-bit ‘tif’ files (800 × 600 pixels). Values obtained for individual data points (and outlier analysis) for summary graphs in are contained in ‘.xlsx’ files. [file elife-62184-fig4-data1.zip › Figure 4- Source Data 1/WT1/P42.WT1.SCGN.tif]

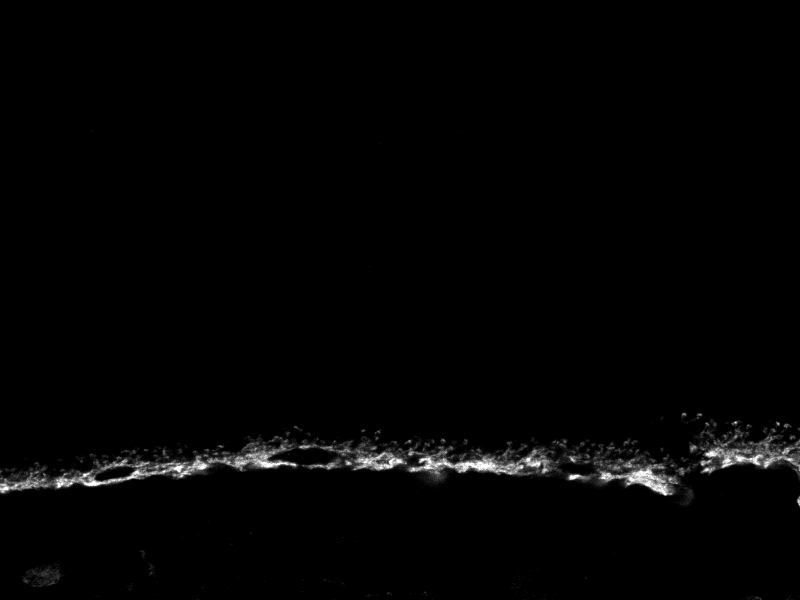

Supplement: Figure 4—source data 1. — The data were exported as 8-bit ‘tif’ files (800 × 600 pixels). Values obtained for individual data points (and outlier analysis) for summary graphs in are contained in ‘.xlsx’ files. [file elife-62184-fig4-data1.zip › Figure 4- Source Data 1/WT2/P42.WT2.calbindin.tif]

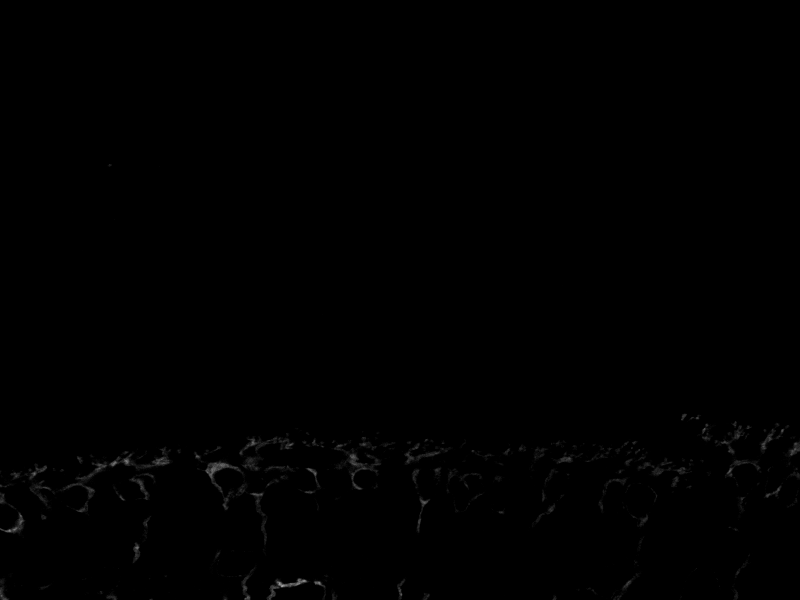

Supplement: Figure 4—source data 1. — The data were exported as 8-bit ‘tif’ files (800 × 600 pixels). Values obtained for individual data points (and outlier analysis) for summary graphs in are contained in ‘.xlsx’ files. [file elife-62184-fig4-data1.zip › Figure 4- Source Data 1/WT2/P42.WT2.PKCa.tif]

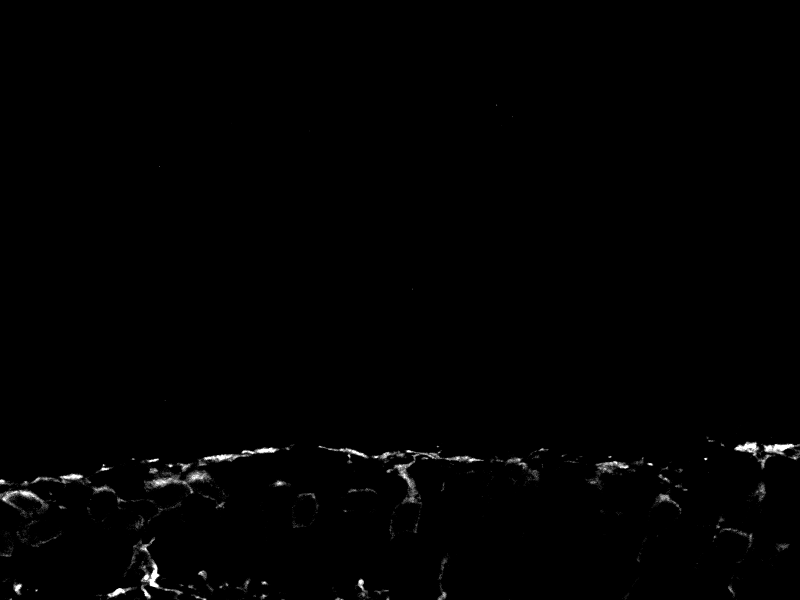

Supplement: Figure 4—source data 1. — The data were exported as 8-bit ‘tif’ files (800 × 600 pixels). Values obtained for individual data points (and outlier analysis) for summary graphs in are contained in ‘.xlsx’ files. [file elife-62184-fig4-data1.zip › Figure 4- Source Data 1/WT2/P42.WT2.SCGN.tif]

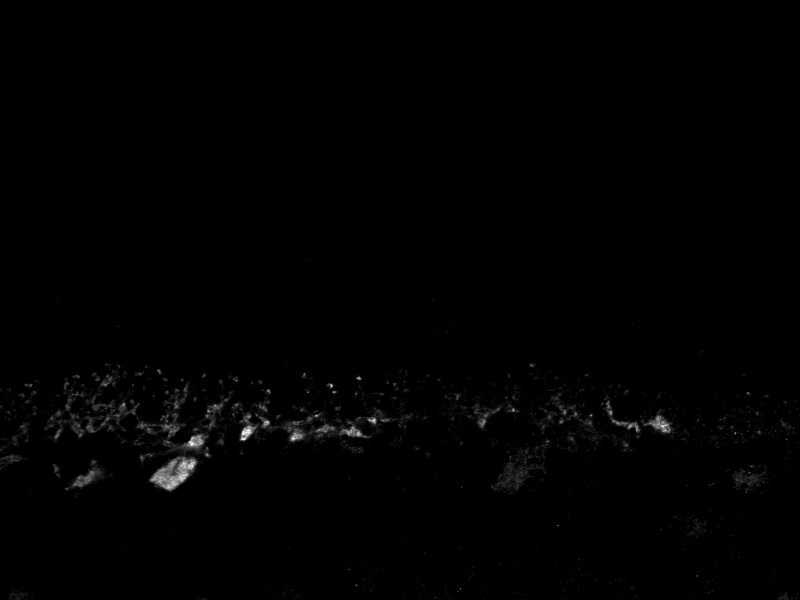

Supplement: Figure 4—source data 1. — The data were exported as 8-bit ‘tif’ files (800 × 600 pixels). Values obtained for individual data points (and outlier analysis) for summary graphs in are contained in ‘.xlsx’ files. [file elife-62184-fig4-data1.zip › Figure 4- Source Data 1/WT3/P42.WT3.calbindin.tif]

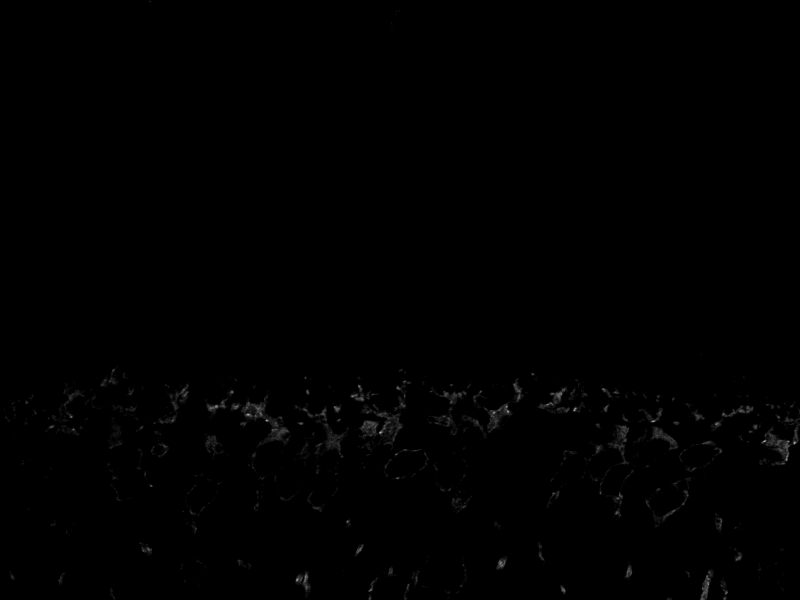

Supplement: Figure 4—source data 1. — The data were exported as 8-bit ‘tif’ files (800 × 600 pixels). Values obtained for individual data points (and outlier analysis) for summary graphs in are contained in ‘.xlsx’ files. [file elife-62184-fig4-data1.zip › Figure 4- Source Data 1/WT3/P42.WT3.PKCa.tif]

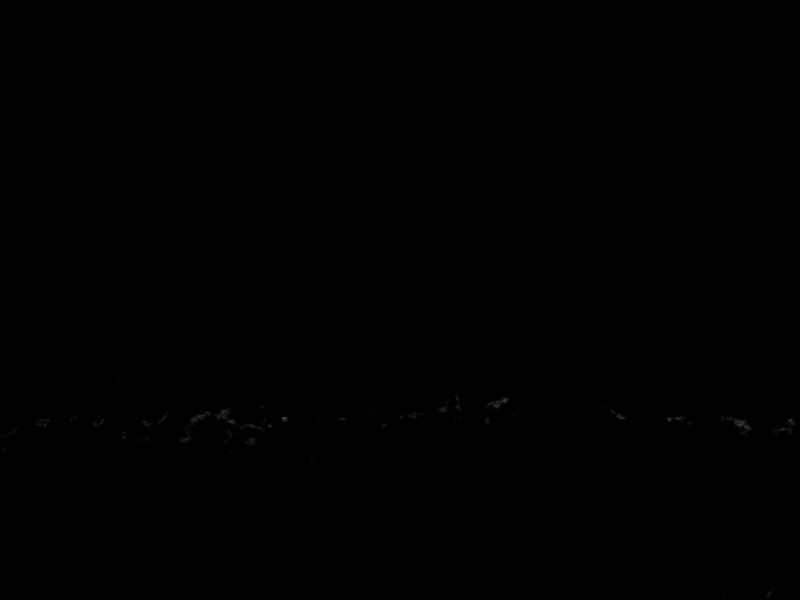

Supplement: Figure 4—source data 1. — The data were exported as 8-bit ‘tif’ files (800 × 600 pixels). Values obtained for individual data points (and outlier analysis) for summary graphs in are contained in ‘.xlsx’ files. [file elife-62184-fig4-data1.zip › Figure 4- Source Data 1/WT3/P42.WT3.SCGN.tif]

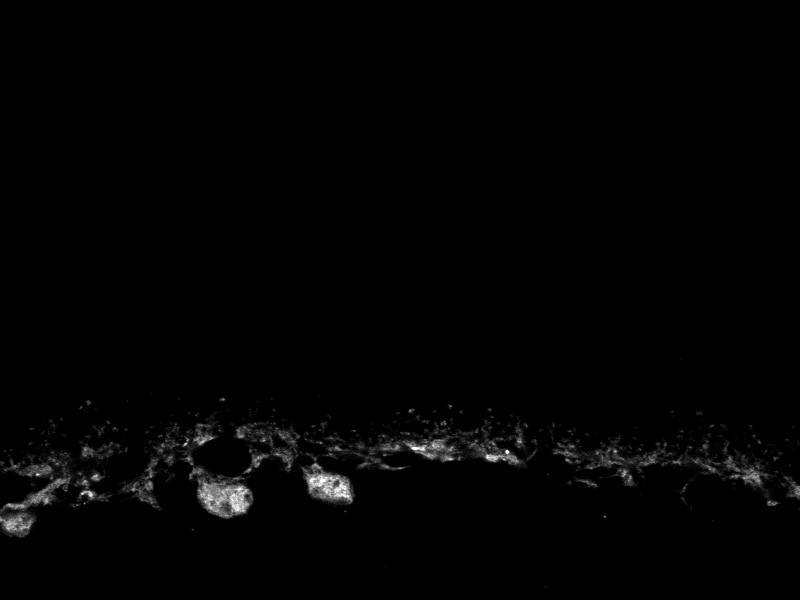

Supplement: Figure 4—source data 1. — The data were exported as 8-bit ‘tif’ files (800 × 600 pixels). Values obtained for individual data points (and outlier analysis) for summary graphs in are contained in ‘.xlsx’ files. [file elife-62184-fig4-data1.zip › Figure 4- Source Data 1/WT4/P42.WT4.calbindin.tif]

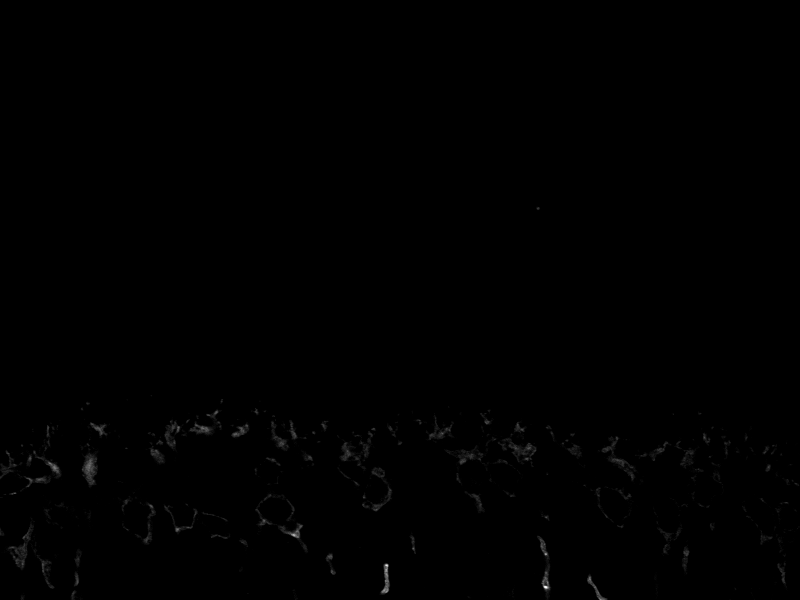

Supplement: Figure 4—source data 1. — The data were exported as 8-bit ‘tif’ files (800 × 600 pixels). Values obtained for individual data points (and outlier analysis) for summary graphs in are contained in ‘.xlsx’ files. [file elife-62184-fig4-data1.zip › Figure 4- Source Data 1/WT4/P42.WT4.PKCa.tif]

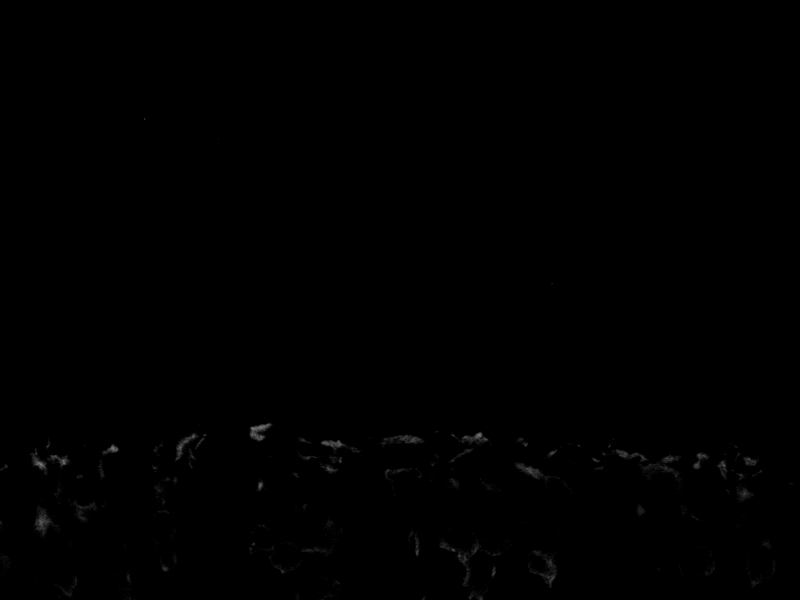

Supplement: Figure 4—source data 1. — The data were exported as 8-bit ‘tif’ files (800 × 600 pixels). Values obtained for individual data points (and outlier analysis) for summary graphs in are contained in ‘.xlsx’ files. [file elife-62184-fig4-data1.zip › Figure 4- Source Data 1/WT4/P42.WT4.SCGN.tif]

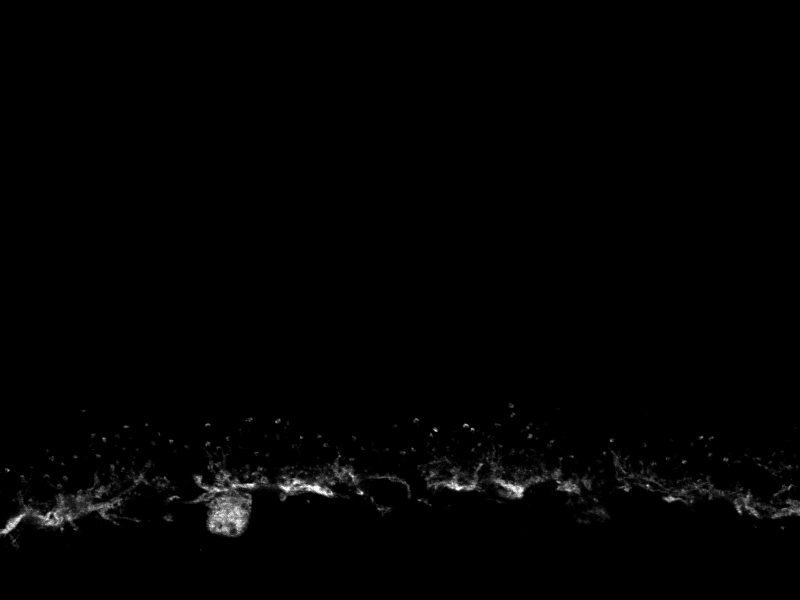

Supplement: Figure 4—source data 1. — The data were exported as 8-bit ‘tif’ files (800 × 600 pixels). Values obtained for individual data points (and outlier analysis) for summary graphs in are contained in ‘.xlsx’ files. [file elife-62184-fig4-data1.zip › Figure 4- Source Data 1/WT5/P42.WT5.calbindin.tif]

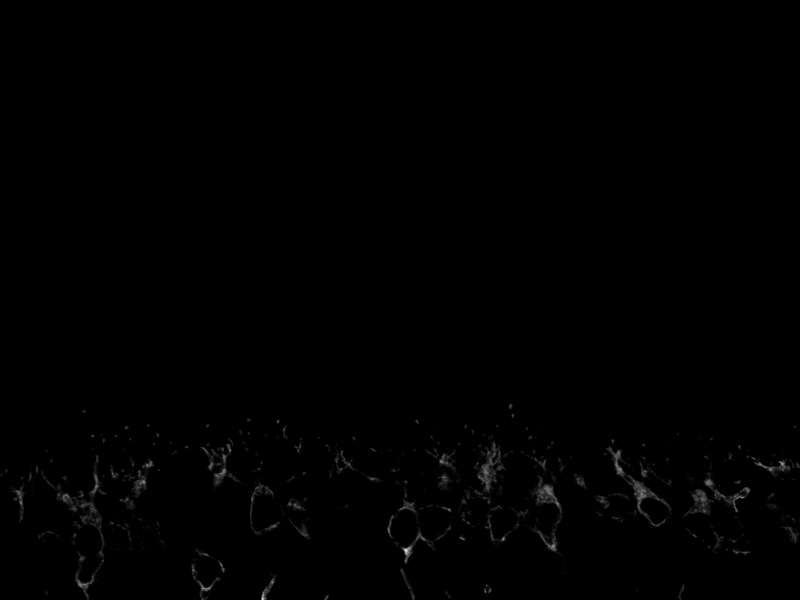

Supplement: Figure 4—source data 1. — The data were exported as 8-bit ‘tif’ files (800 × 600 pixels). Values obtained for individual data points (and outlier analysis) for summary graphs in are contained in ‘.xlsx’ files. [file elife-62184-fig4-data1.zip › Figure 4- Source Data 1/WT5/P42.WT5.PKCa.tif]

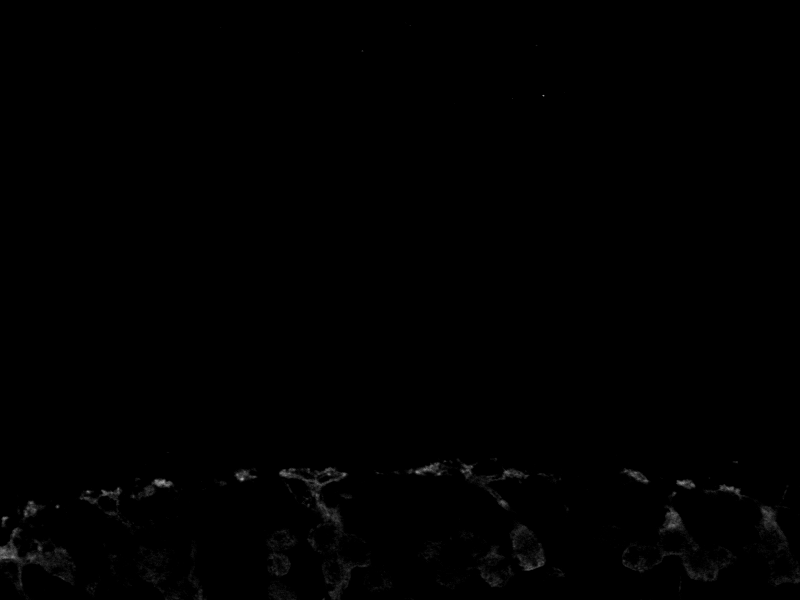

Supplement: Figure 4—source data 1. — The data were exported as 8-bit ‘tif’ files (800 × 600 pixels). Values obtained for individual data points (and outlier analysis) for summary graphs in are contained in ‘.xlsx’ files. [file elife-62184-fig4-data1.zip › Figure 4- Source Data 1/WT5/P42.WT5.SCGN.tif]

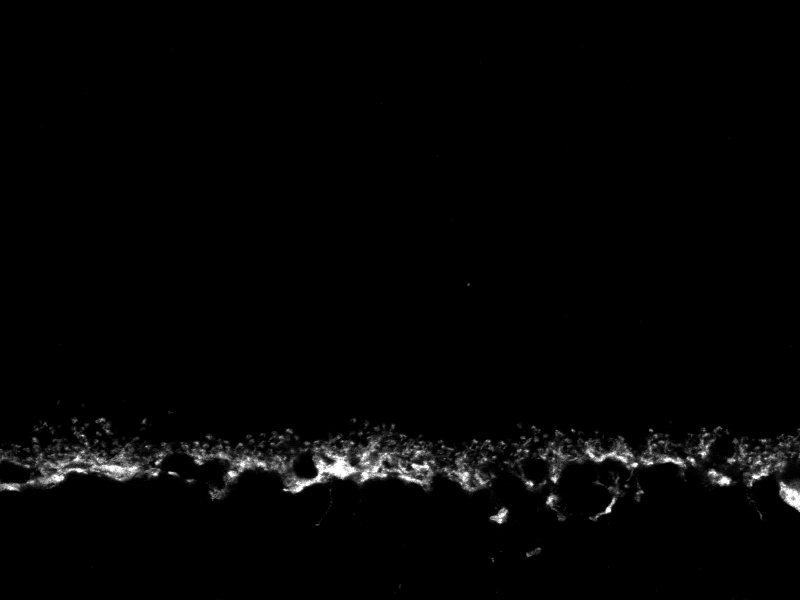

Supplement: Figure 4—source data 1. — The data were exported as 8-bit ‘tif’ files (800 × 600 pixels). Values obtained for individual data points (and outlier analysis) for summary graphs in are contained in ‘.xlsx’ files. [file elife-62184-fig4-data1.zip › Figure 4- Source Data 1/WT6/P42.WT6.calbindin.tif]

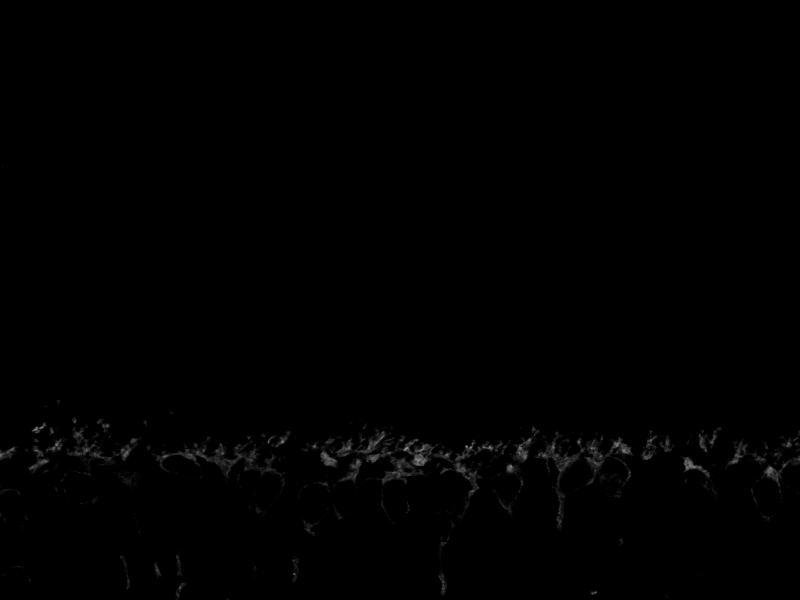

Supplement: Figure 4—source data 1. — The data were exported as 8-bit ‘tif’ files (800 × 600 pixels). Values obtained for individual data points (and outlier analysis) for summary graphs in are contained in ‘.xlsx’ files. [file elife-62184-fig4-data1.zip › Figure 4- Source Data 1/WT6/P42.WT6.PKCa.tif]

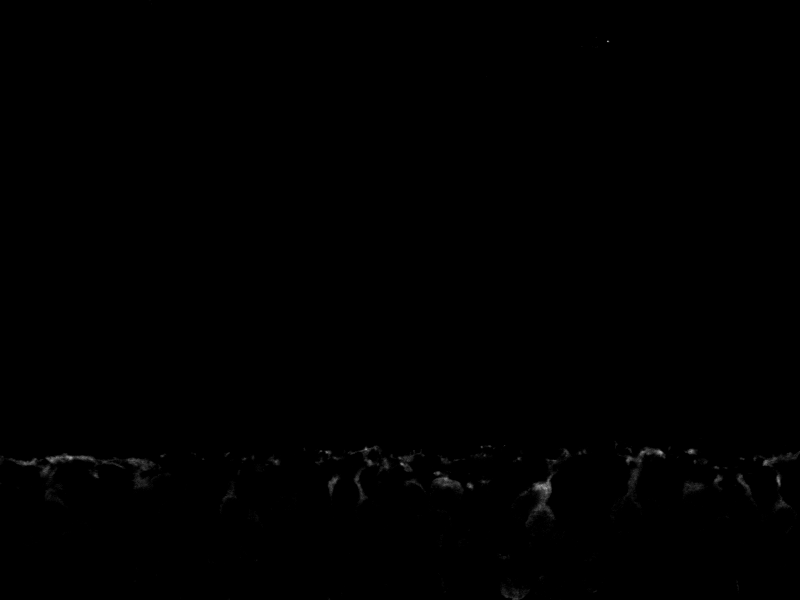

Supplement: Figure 4—source data 1. — The data were exported as 8-bit ‘tif’ files (800 × 600 pixels). Values obtained for individual data points (and outlier analysis) for summary graphs in are contained in ‘.xlsx’ files. [file elife-62184-fig4-data1.zip › Figure 4- Source Data 1/WT6/P42.WT6.SCGN.tif]

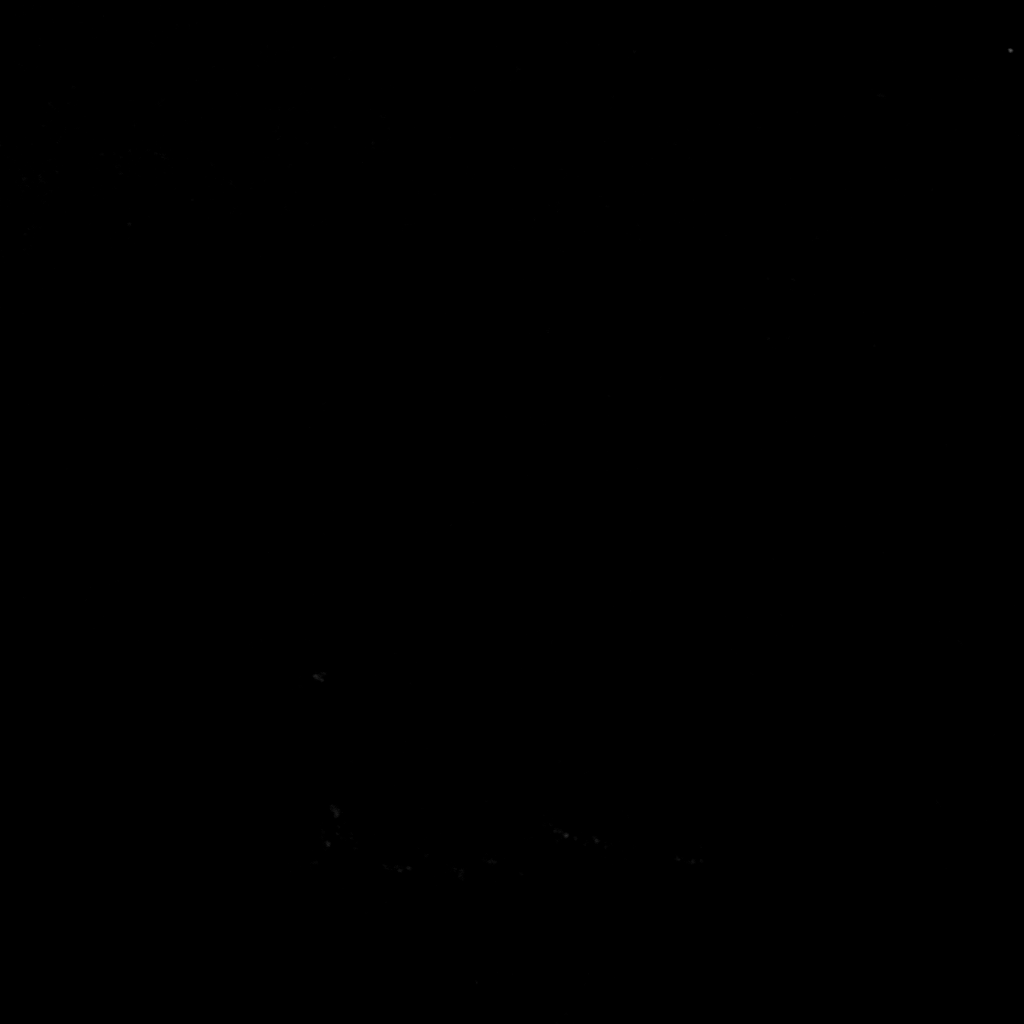

Supplement: Figure 4—figure supplement 2—source data 1. — The data were exported as 8-bit ‘tif’ files (1024 × 1024 pixels). Values for individual data points (and outlier analysis) for summary graphs in Figure 4—figure supplement 2B are Figure 4—figure supplement 2C contained in excel files. [file elife-62184-fig4-figsupp2-data1.zip › Figure 4- Figure supplement 2- Source Data 1/KI1/P42KI1.mglur6.tif]

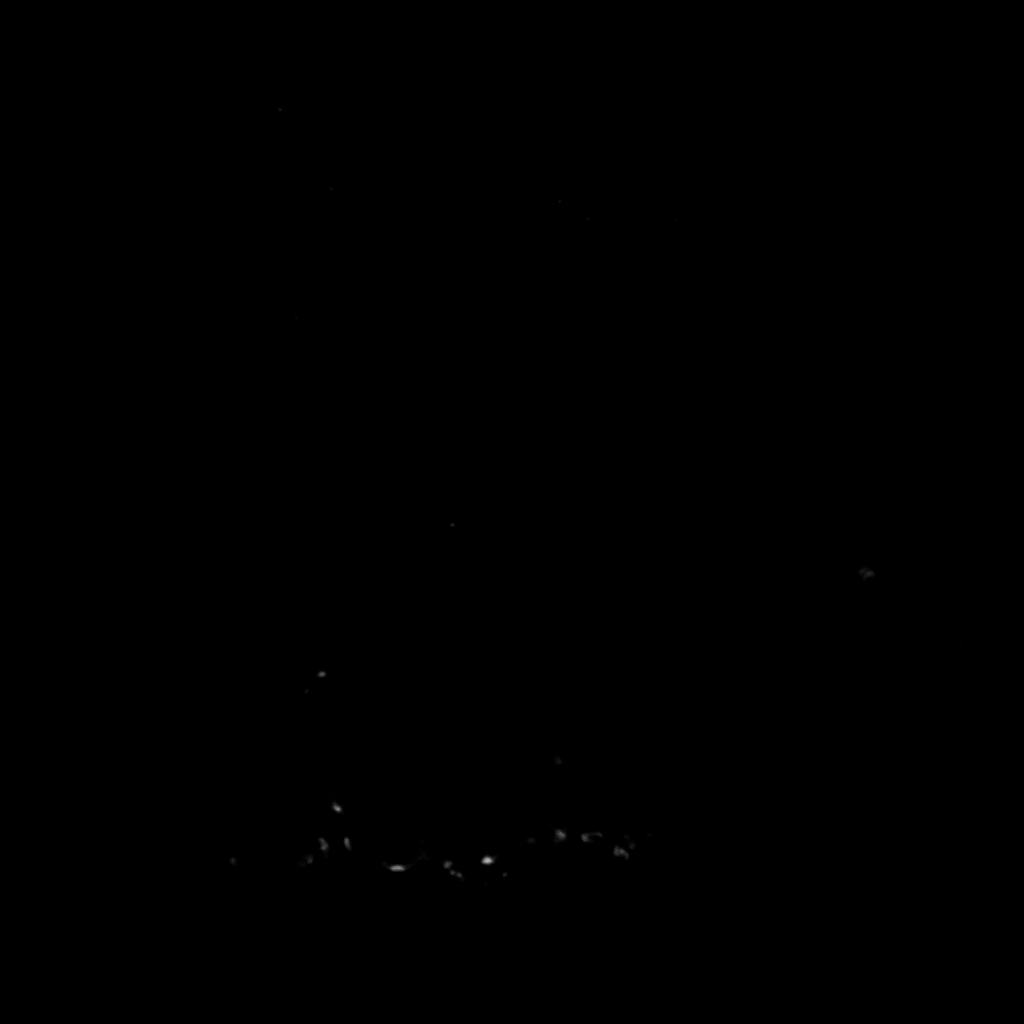

Supplement: Figure 4—figure supplement 2—source data 1. — The data were exported as 8-bit ‘tif’ files (1024 × 1024 pixels). Values for individual data points (and outlier analysis) for summary graphs in Figure 4—figure supplement 2B are Figure 4—figure supplement 2C contained in excel files. [file elife-62184-fig4-figsupp2-data1.zip › Figure 4- Figure supplement 2- Source Data 1/KI1/P42KI1.ribeye.tif]

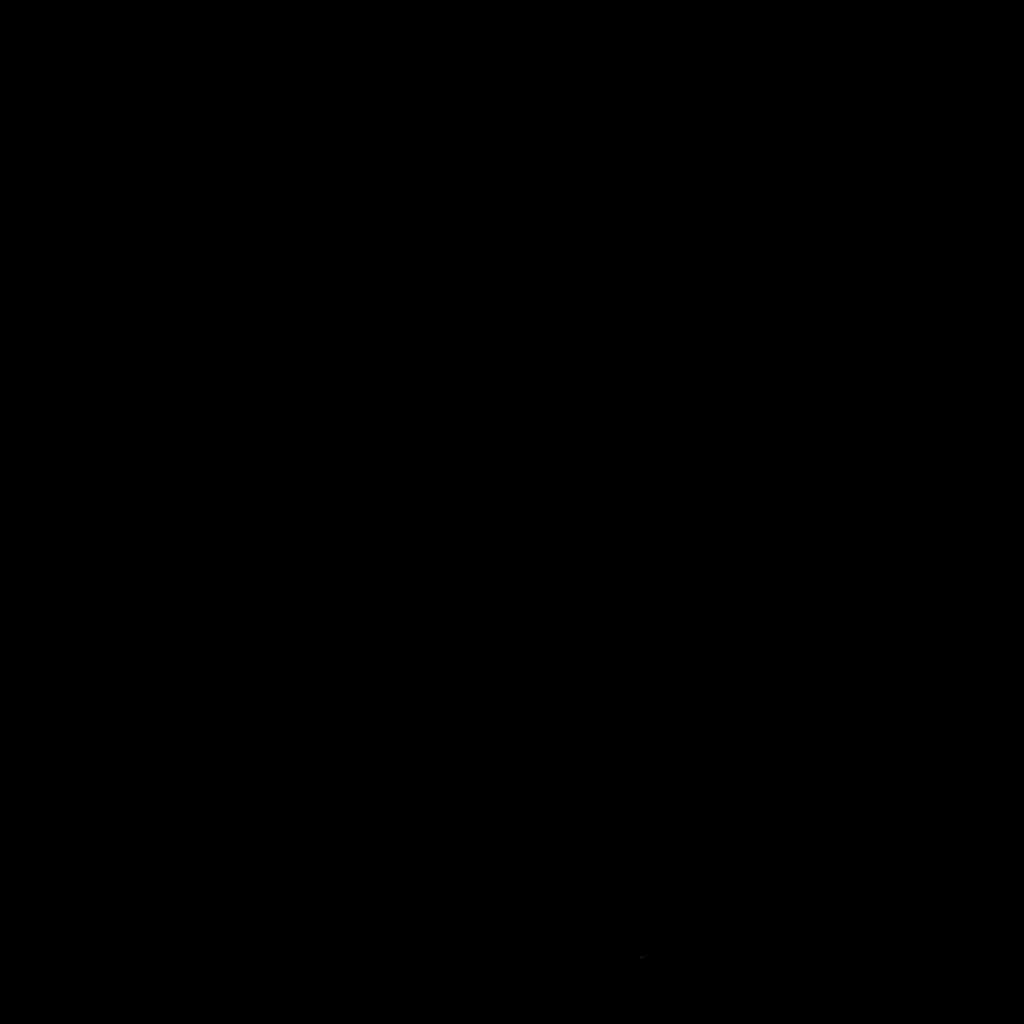

Supplement: Figure 4—figure supplement 2—source data 1. — The data were exported as 8-bit ‘tif’ files (1024 × 1024 pixels). Values for individual data points (and outlier analysis) for summary graphs in Figure 4—figure supplement 2B are Figure 4—figure supplement 2C contained in excel files. [file elife-62184-fig4-figsupp2-data1.zip › Figure 4- Figure supplement 2- Source Data 1/KI2/P42KI2.mglur6.tif]

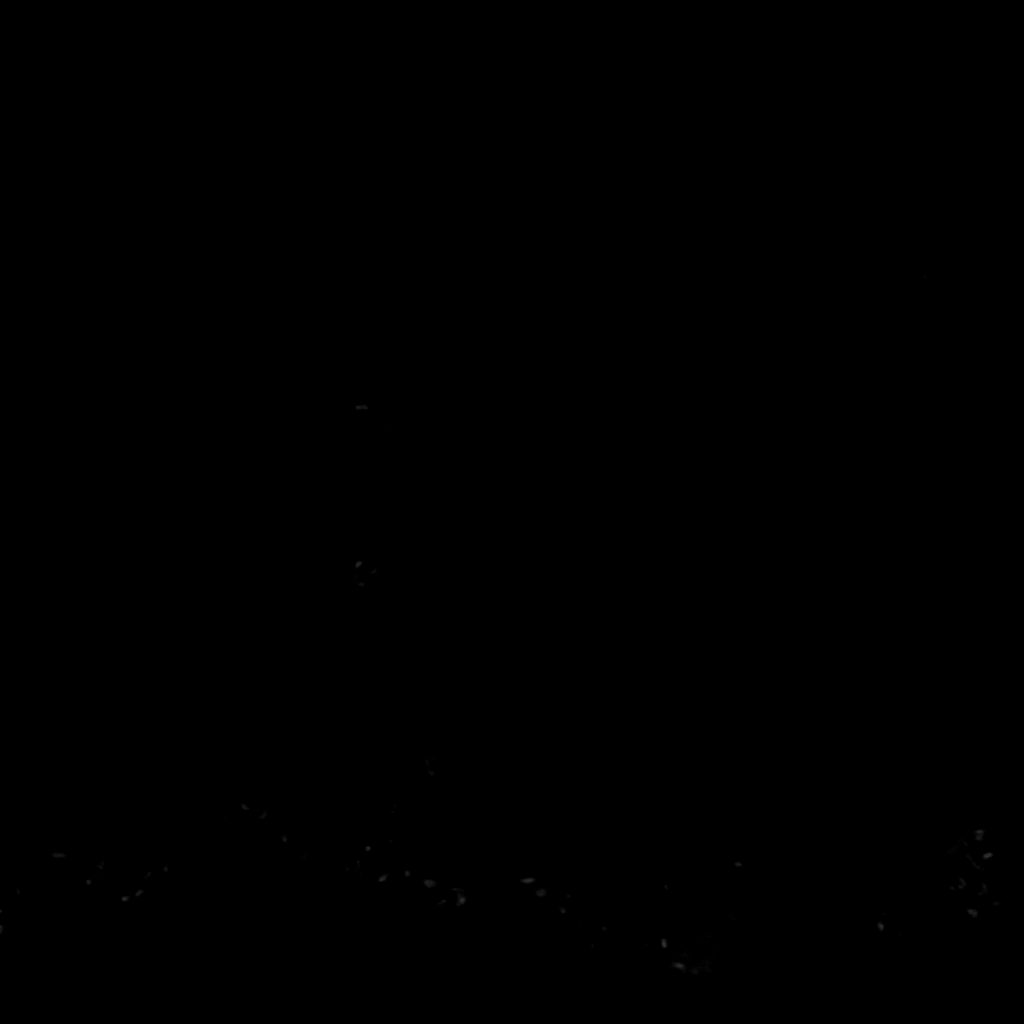

Supplement: Figure 4—figure supplement 2—source data 1. — The data were exported as 8-bit ‘tif’ files (1024 × 1024 pixels). Values for individual data points (and outlier analysis) for summary graphs in Figure 4—figure supplement 2B are Figure 4—figure supplement 2C contained in excel files. [file elife-62184-fig4-figsupp2-data1.zip › Figure 4- Figure supplement 2- Source Data 1/KI2/P42KI2.ribeye.tif]

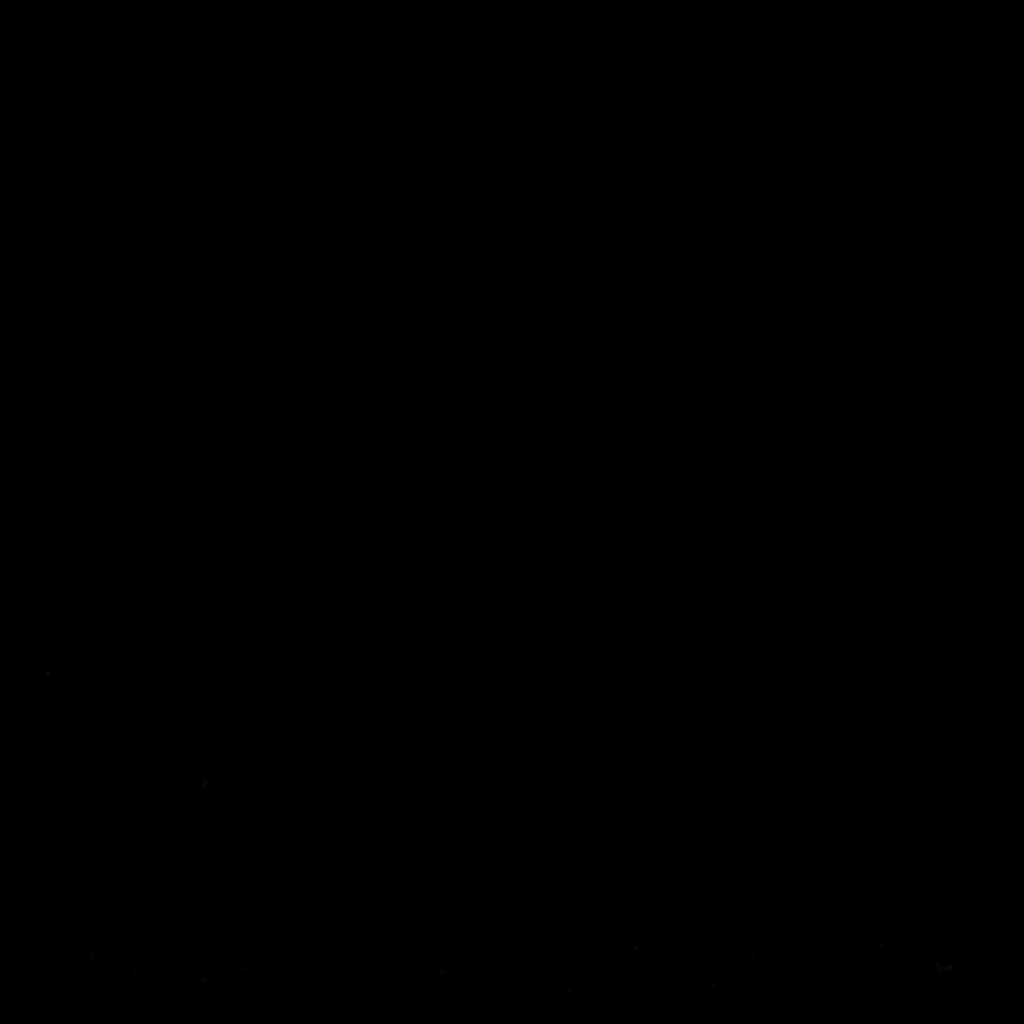

Supplement: Figure 4—figure supplement 2—source data 1. — The data were exported as 8-bit ‘tif’ files (1024 × 1024 pixels). Values for individual data points (and outlier analysis) for summary graphs in Figure 4—figure supplement 2B are Figure 4—figure supplement 2C contained in excel files. [file elife-62184-fig4-figsupp2-data1.zip › Figure 4- Figure supplement 2- Source Data 1/KI3/P42KI3.mglur6.tif]

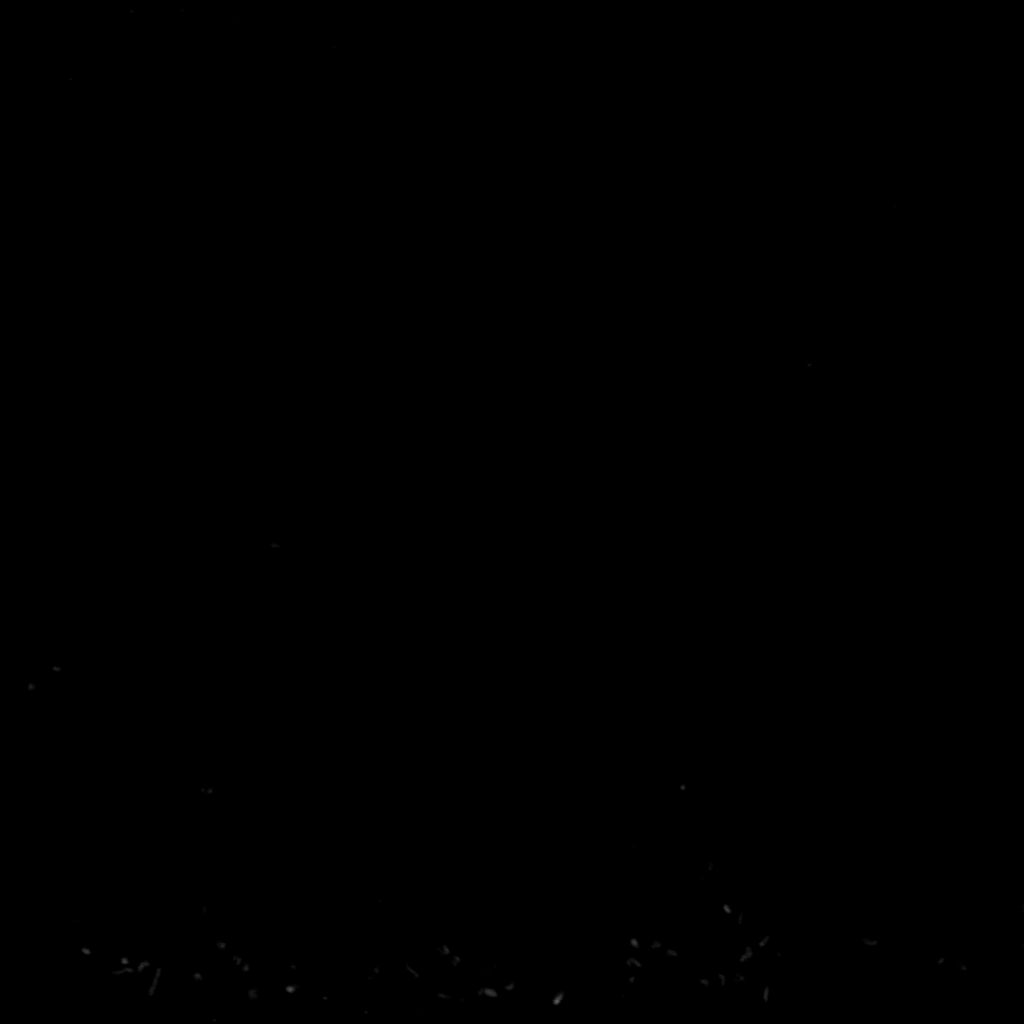

Supplement: Figure 4—figure supplement 2—source data 1. — The data were exported as 8-bit ‘tif’ files (1024 × 1024 pixels). Values for individual data points (and outlier analysis) for summary graphs in Figure 4—figure supplement 2B are Figure 4—figure supplement 2C contained in excel files. [file elife-62184-fig4-figsupp2-data1.zip › Figure 4- Figure supplement 2- Source Data 1/KI3/P42KI3.ribeye.tif]

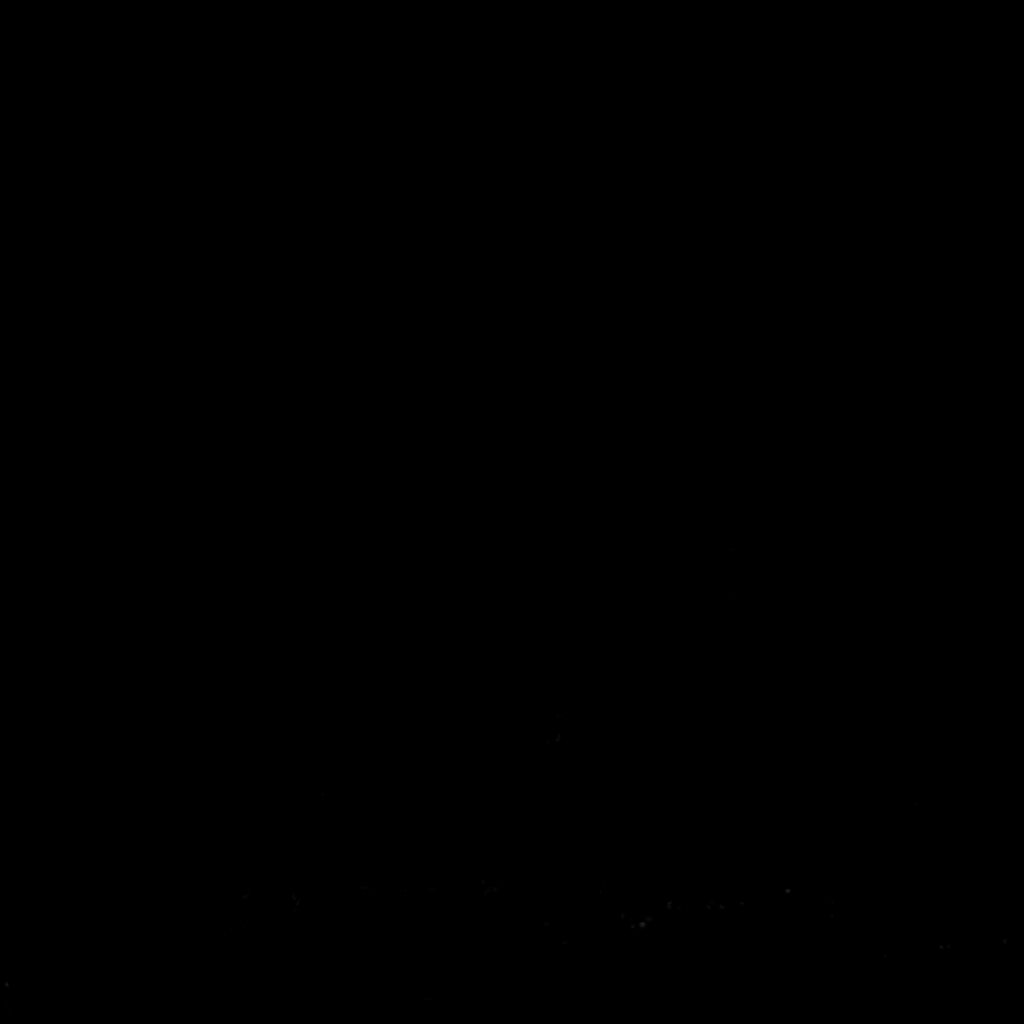

Supplement: Figure 4—figure supplement 2—source data 1. — The data were exported as 8-bit ‘tif’ files (1024 × 1024 pixels). Values for individual data points (and outlier analysis) for summary graphs in Figure 4—figure supplement 2B are Figure 4—figure supplement 2C contained in excel files. [file elife-62184-fig4-figsupp2-data1.zip › Figure 4- Figure supplement 2- Source Data 1/KI4/P42KI4.mglur6.tif]

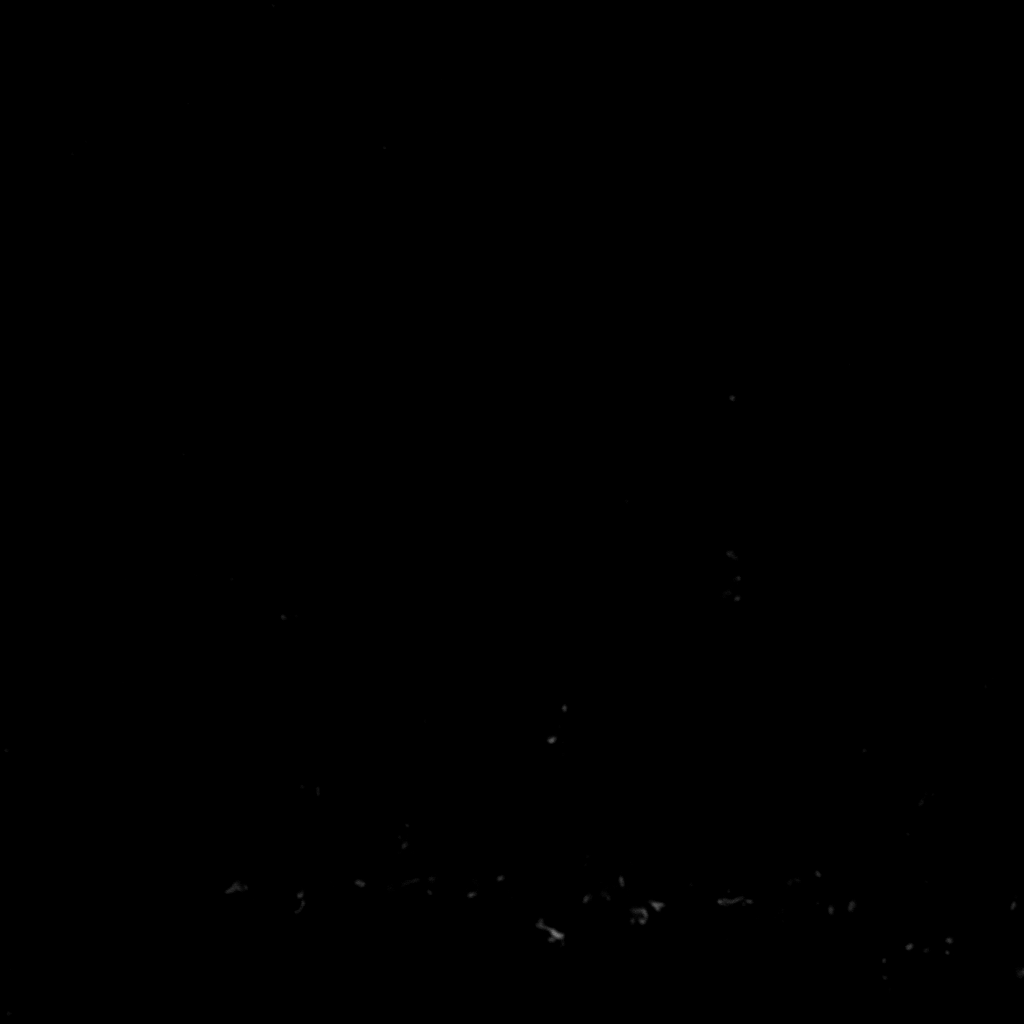

Supplement: Figure 4—figure supplement 2—source data 1. — The data were exported as 8-bit ‘tif’ files (1024 × 1024 pixels). Values for individual data points (and outlier analysis) for summary graphs in Figure 4—figure supplement 2B are Figure 4—figure supplement 2C contained in excel files. [file elife-62184-fig4-figsupp2-data1.zip › Figure 4- Figure supplement 2- Source Data 1/KI4/P42KI4.ribeye.tif]

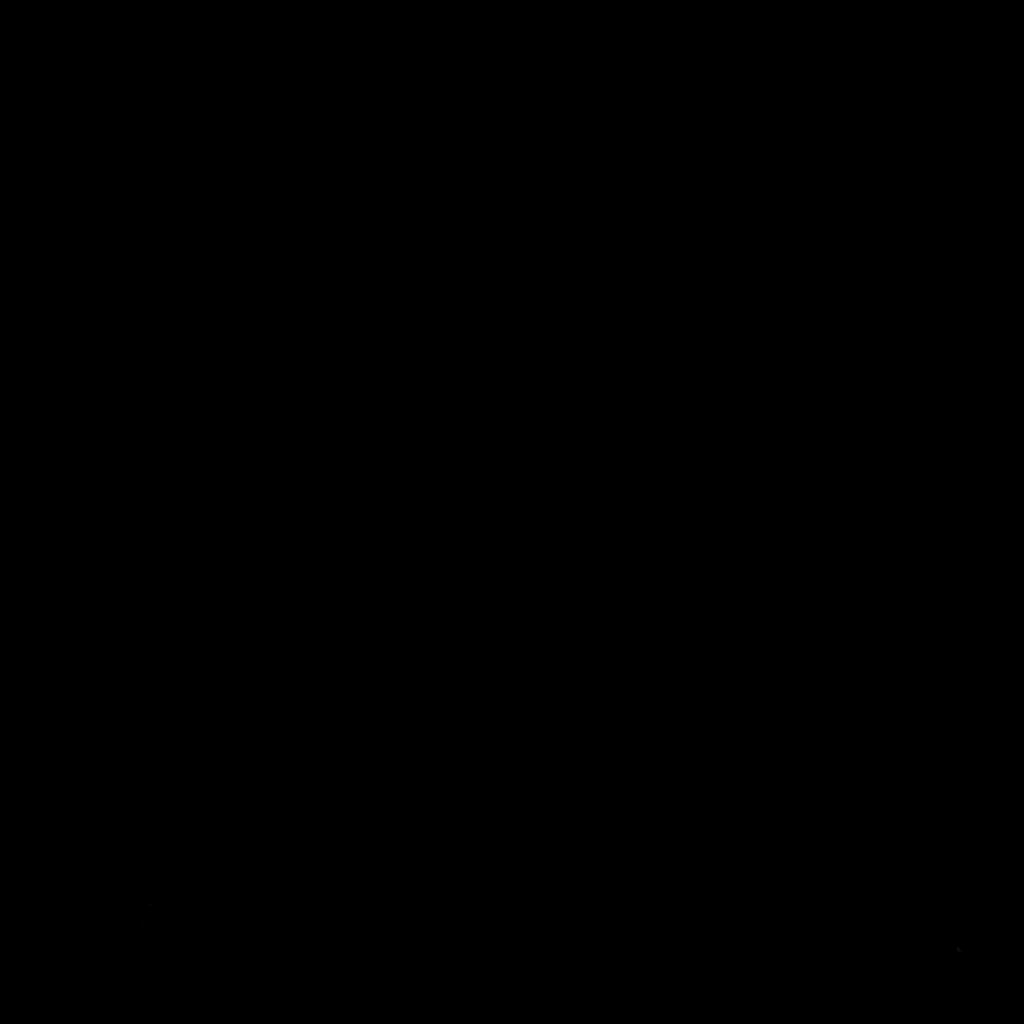

Supplement: Figure 4—figure supplement 2—source data 1. — The data were exported as 8-bit ‘tif’ files (1024 × 1024 pixels). Values for individual data points (and outlier analysis) for summary graphs in Figure 4—figure supplement 2B are Figure 4—figure supplement 2C contained in excel files. [file elife-62184-fig4-figsupp2-data1.zip › Figure 4- Figure supplement 2- Source Data 1/KO1/P42KO2.mglur6.tif]

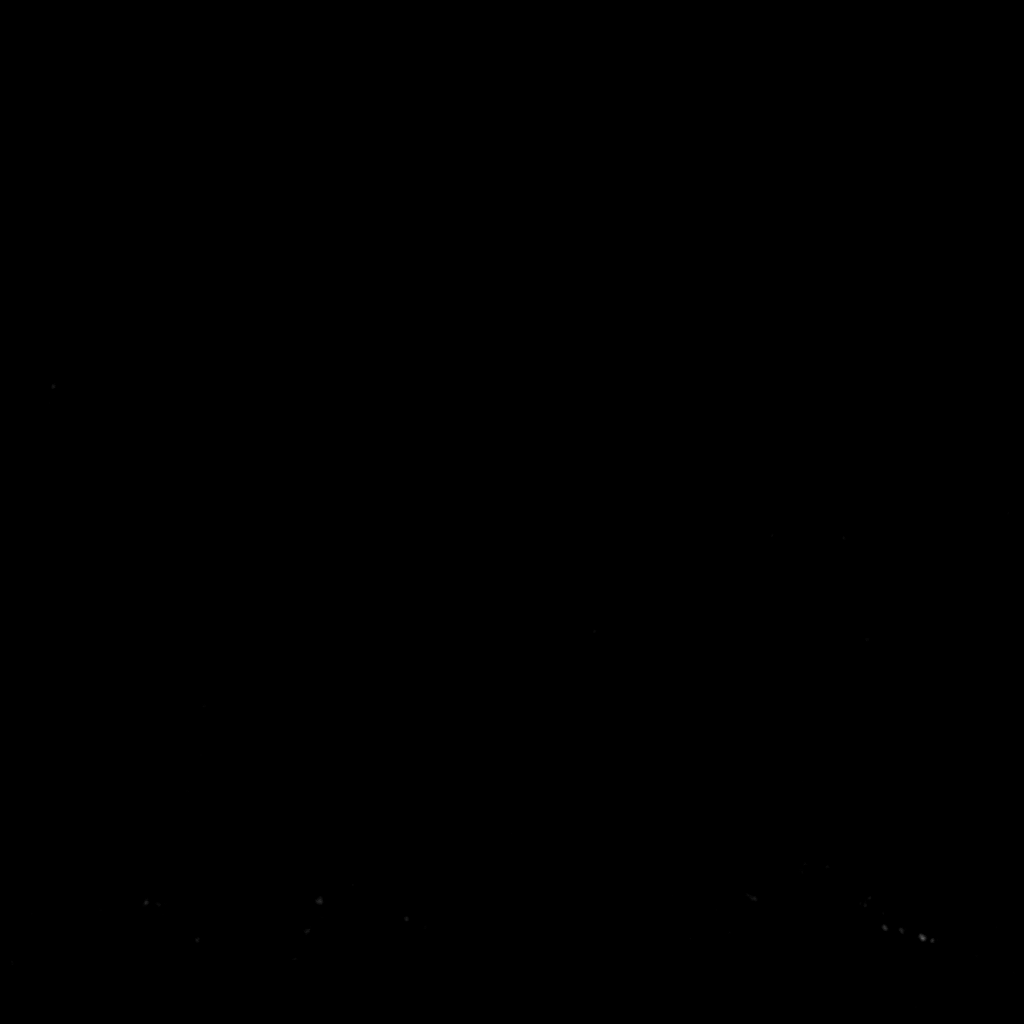

Supplement: Figure 4—figure supplement 2—source data 1. — The data were exported as 8-bit ‘tif’ files (1024 × 1024 pixels). Values for individual data points (and outlier analysis) for summary graphs in Figure 4—figure supplement 2B are Figure 4—figure supplement 2C contained in excel files. [file elife-62184-fig4-figsupp2-data1.zip › Figure 4- Figure supplement 2- Source Data 1/KO1/P42KO2.ribeye.tif]

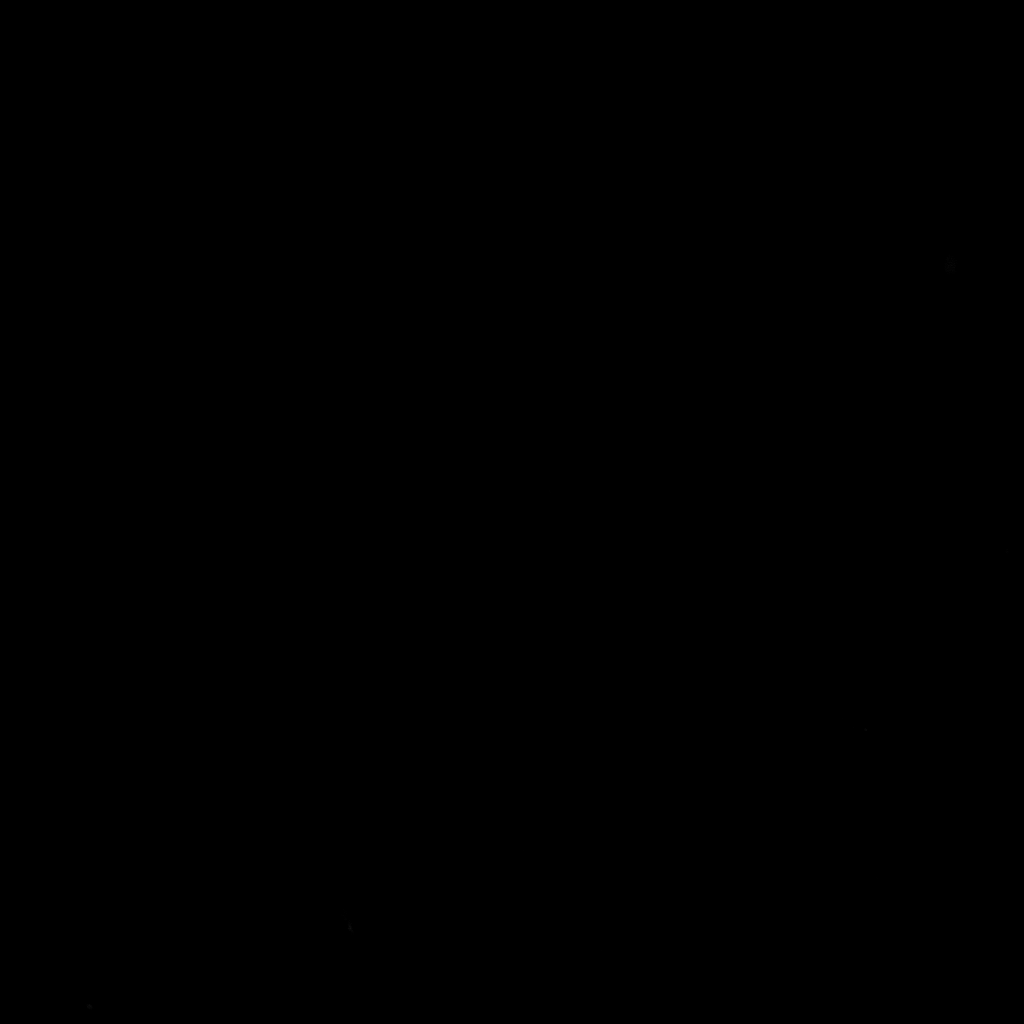

Supplement: Figure 4—figure supplement 2—source data 1. — The data were exported as 8-bit ‘tif’ files (1024 × 1024 pixels). Values for individual data points (and outlier analysis) for summary graphs in Figure 4—figure supplement 2B are Figure 4—figure supplement 2C contained in excel files. [file elife-62184-fig4-figsupp2-data1.zip › Figure 4- Figure supplement 2- Source Data 1/KO2/P42KO3.mglur6.tif]

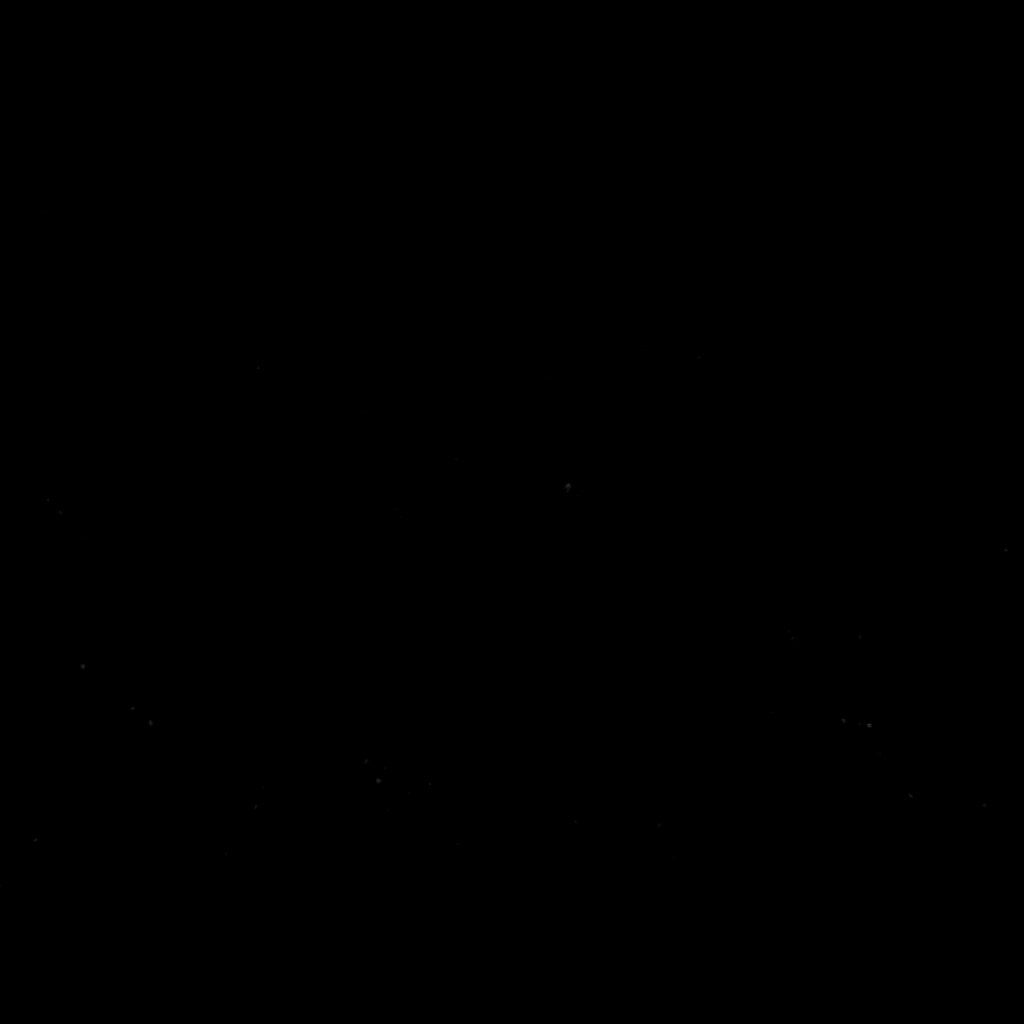

Supplement: Figure 4—figure supplement 2—source data 1. — The data were exported as 8-bit ‘tif’ files (1024 × 1024 pixels). Values for individual data points (and outlier analysis) for summary graphs in Figure 4—figure supplement 2B are Figure 4—figure supplement 2C contained in excel files. [file elife-62184-fig4-figsupp2-data1.zip › Figure 4- Figure supplement 2- Source Data 1/KO2/P42KO3.ribeye.tif]

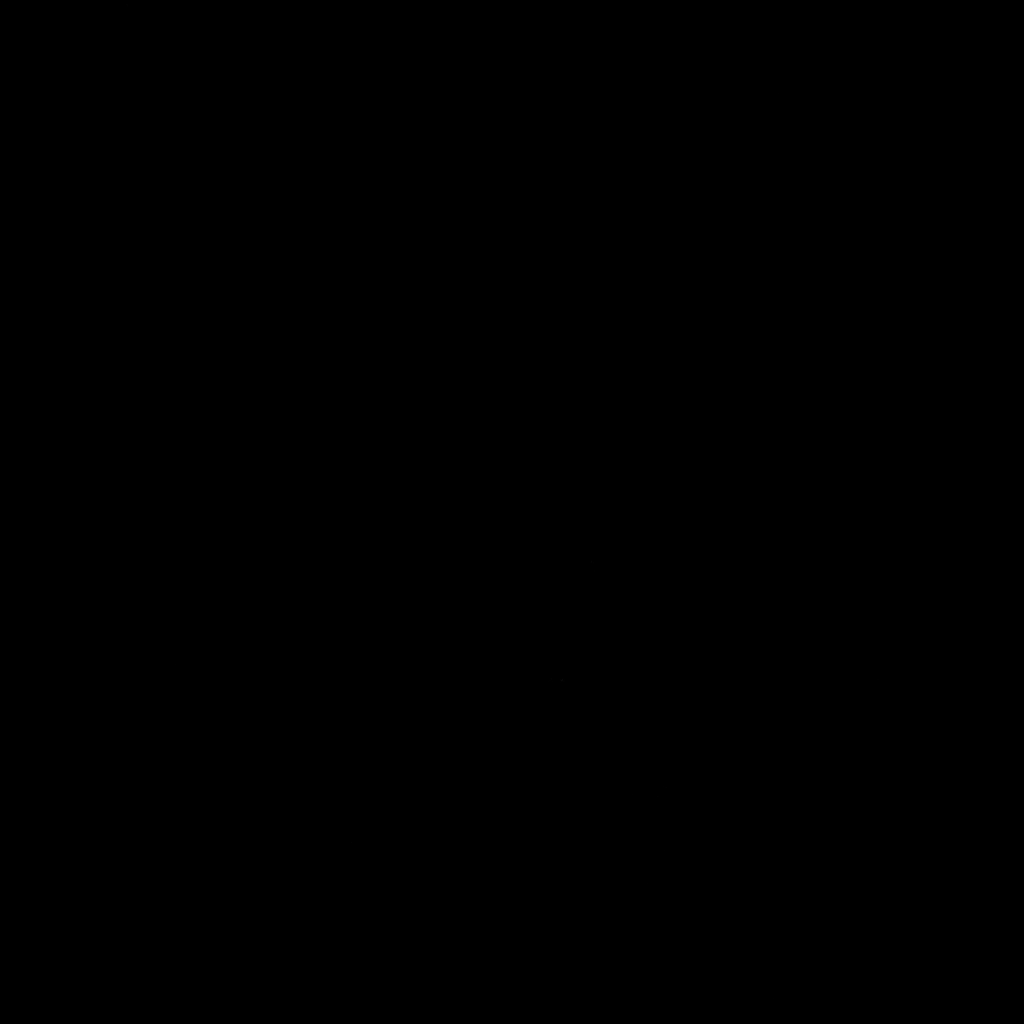

Supplement: Figure 4—figure supplement 2—source data 1. — The data were exported as 8-bit ‘tif’ files (1024 × 1024 pixels). Values for individual data points (and outlier analysis) for summary graphs in Figure 4—figure supplement 2B are Figure 4—figure supplement 2C contained in excel files. [file elife-62184-fig4-figsupp2-data1.zip › Figure 4- Figure supplement 2- Source Data 1/KO3/P42KO4.mglur6.tif]

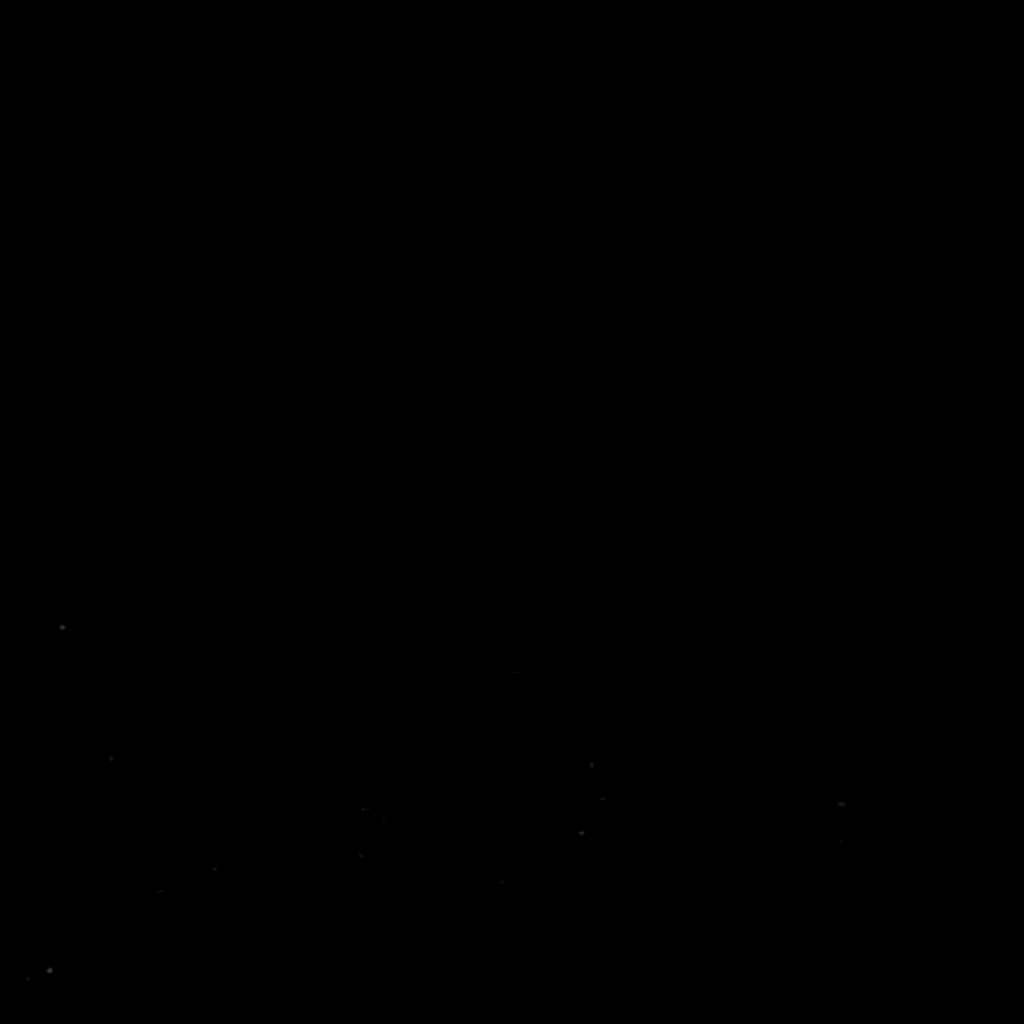

Supplement: Figure 4—figure supplement 2—source data 1. — The data were exported as 8-bit ‘tif’ files (1024 × 1024 pixels). Values for individual data points (and outlier analysis) for summary graphs in Figure 4—figure supplement 2B are Figure 4—figure supplement 2C contained in excel files. [file elife-62184-fig4-figsupp2-data1.zip › Figure 4- Figure supplement 2- Source Data 1/KO3/P42KO4.ribeye.tif]

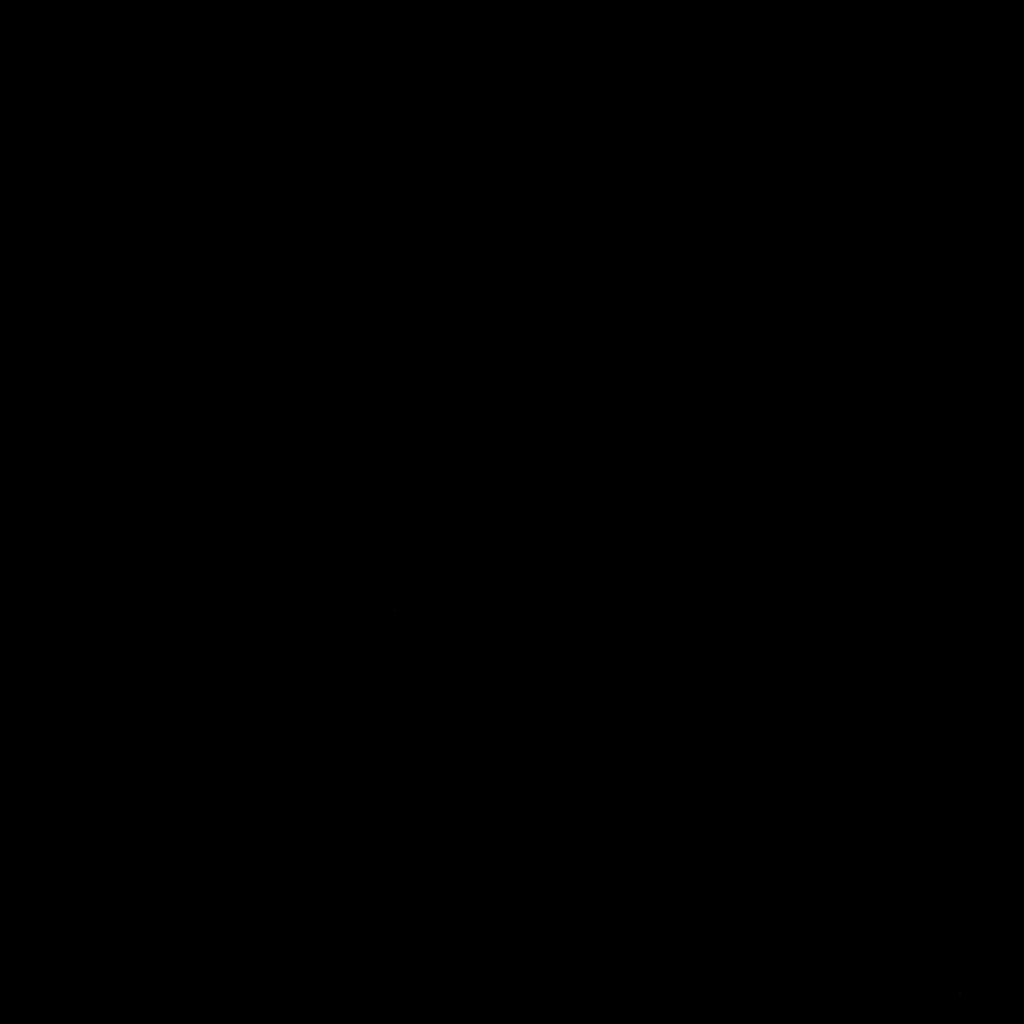

Supplement: Figure 4—figure supplement 2—source data 1. — The data were exported as 8-bit ‘tif’ files (1024 × 1024 pixels). Values for individual data points (and outlier analysis) for summary graphs in Figure 4—figure supplement 2B are Figure 4—figure supplement 2C contained in excel files. [file elife-62184-fig4-figsupp2-data1.zip › Figure 4- Figure supplement 2- Source Data 1/KO4/P42KO6.mglur6.tif]

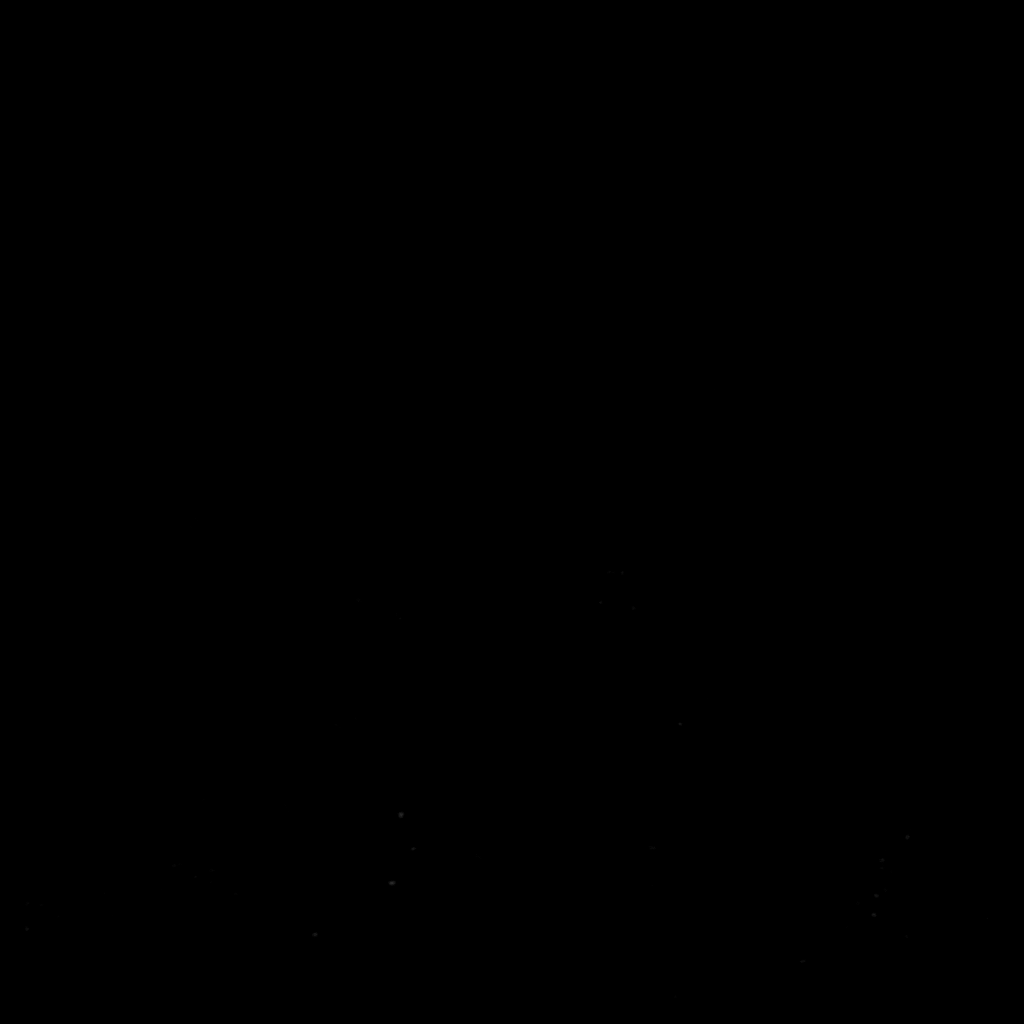

Supplement: Figure 4—figure supplement 2—source data 1. — The data were exported as 8-bit ‘tif’ files (1024 × 1024 pixels). Values for individual data points (and outlier analysis) for summary graphs in Figure 4—figure supplement 2B are Figure 4—figure supplement 2C contained in excel files. [file elife-62184-fig4-figsupp2-data1.zip › Figure 4- Figure supplement 2- Source Data 1/KO4/P42KO6.ribeye.tif]

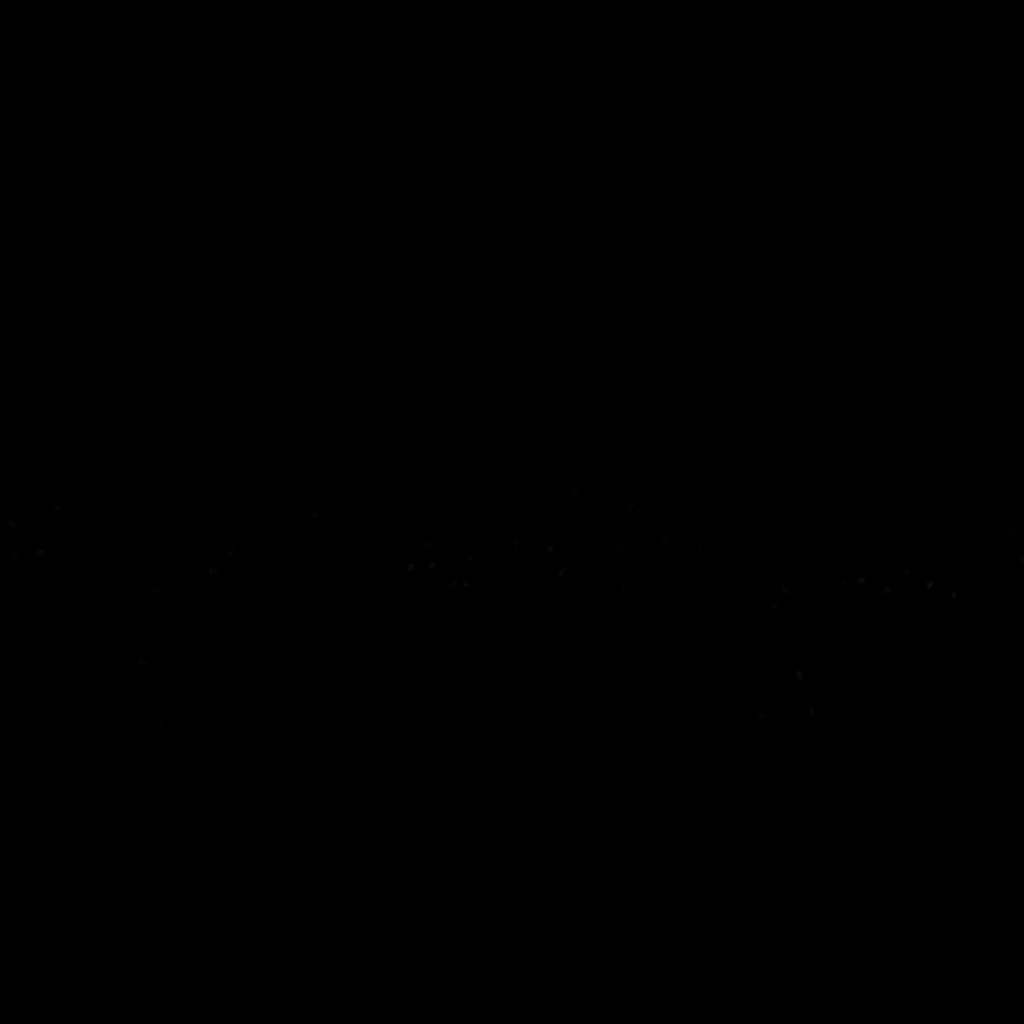

Supplement: Figure 4—figure supplement 2—source data 1. — The data were exported as 8-bit ‘tif’ files (1024 × 1024 pixels). Values for individual data points (and outlier analysis) for summary graphs in Figure 4—figure supplement 2B are Figure 4—figure supplement 2C contained in excel files. [file elife-62184-fig4-figsupp2-data1.zip › Figure 4- Figure supplement 2- Source Data 1/WT1/P42WT1.mglur6.tif]

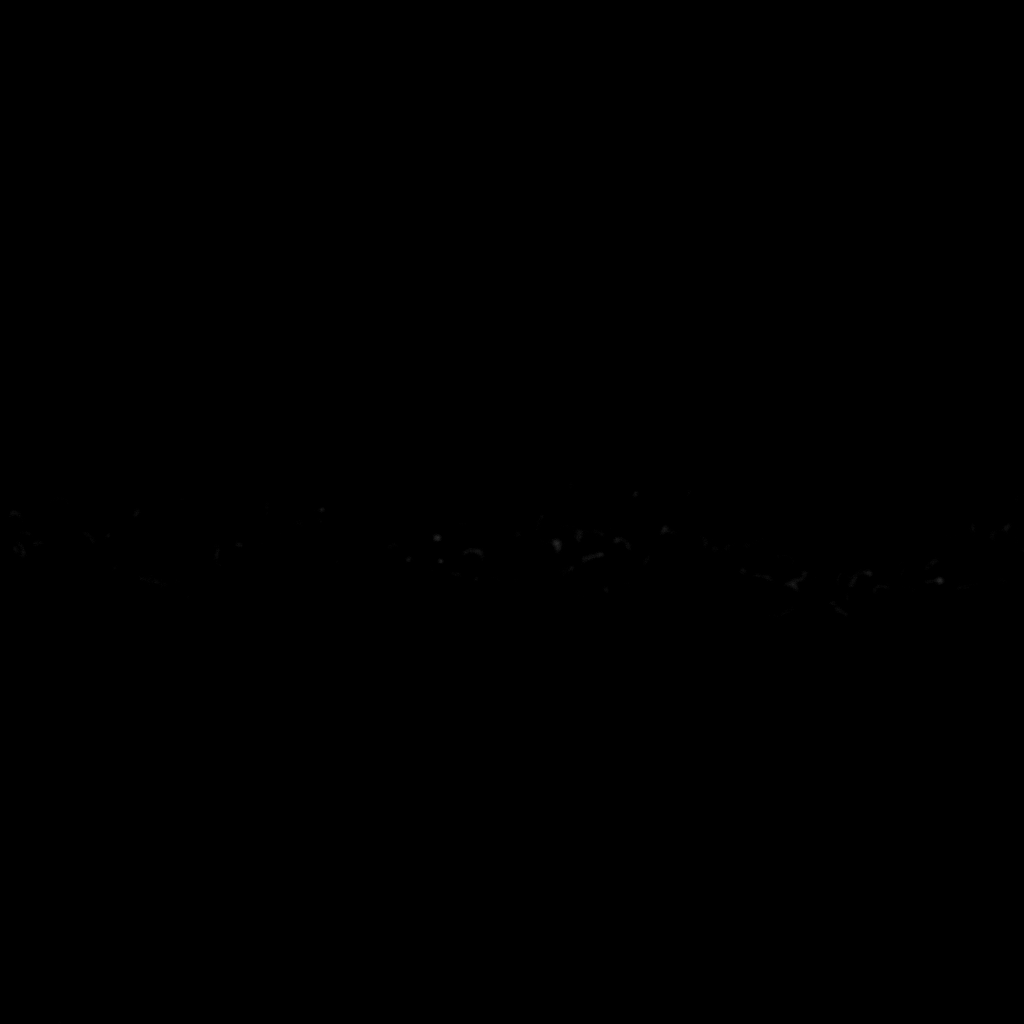

Supplement: Figure 4—figure supplement 2—source data 1. — The data were exported as 8-bit ‘tif’ files (1024 × 1024 pixels). Values for individual data points (and outlier analysis) for summary graphs in Figure 4—figure supplement 2B are Figure 4—figure supplement 2C contained in excel files. [file elife-62184-fig4-figsupp2-data1.zip › Figure 4- Figure supplement 2- Source Data 1/WT1/P42WT1.ribeye.tif]

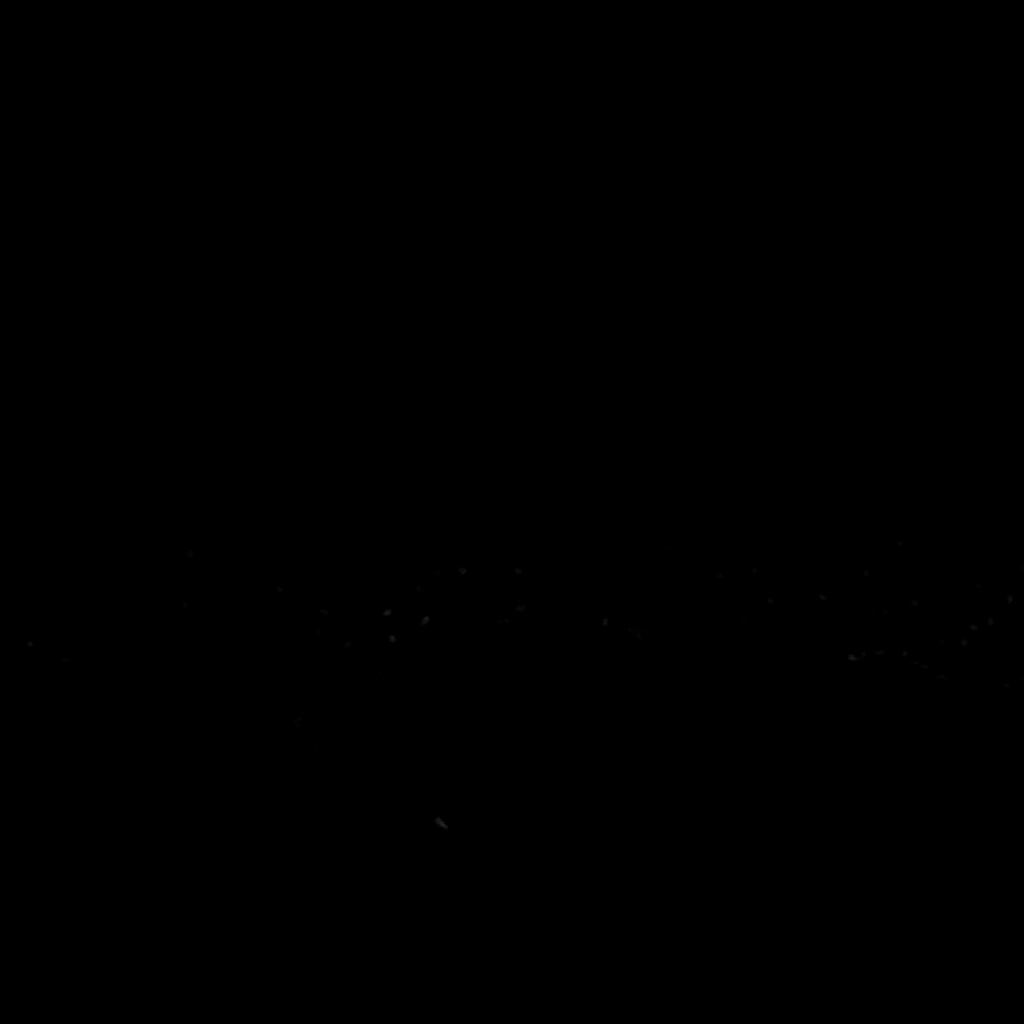

Supplement: Figure 4—figure supplement 2—source data 1. — The data were exported as 8-bit ‘tif’ files (1024 × 1024 pixels). Values for individual data points (and outlier analysis) for summary graphs in Figure 4—figure supplement 2B are Figure 4—figure supplement 2C contained in excel files. [file elife-62184-fig4-figsupp2-data1.zip › Figure 4- Figure supplement 2- Source Data 1/WT2/P42WT2.mglur6.tif]

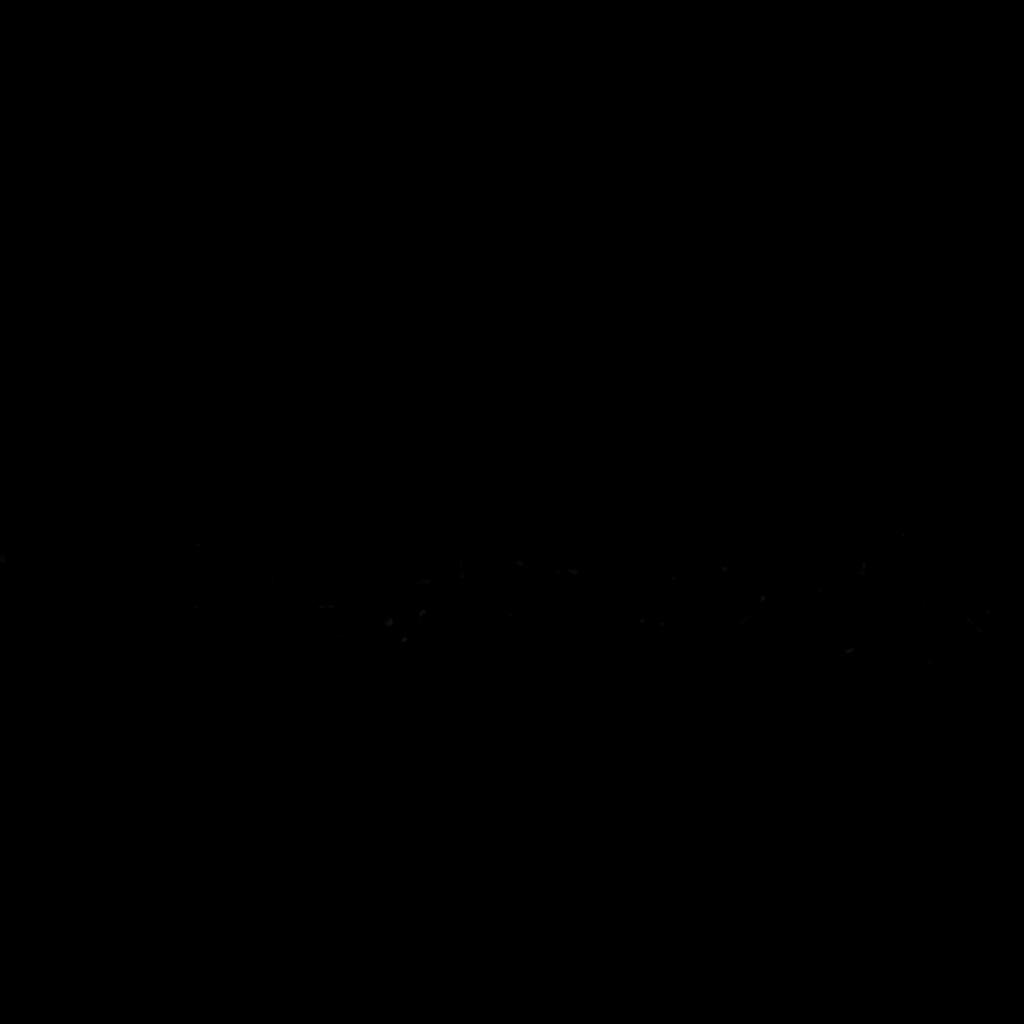

Supplement: Figure 4—figure supplement 2—source data 1. — The data were exported as 8-bit ‘tif’ files (1024 × 1024 pixels). Values for individual data points (and outlier analysis) for summary graphs in Figure 4—figure supplement 2B are Figure 4—figure supplement 2C contained in excel files. [file elife-62184-fig4-figsupp2-data1.zip › Figure 4- Figure supplement 2- Source Data 1/WT2/P42WT2.ribeye.tif]

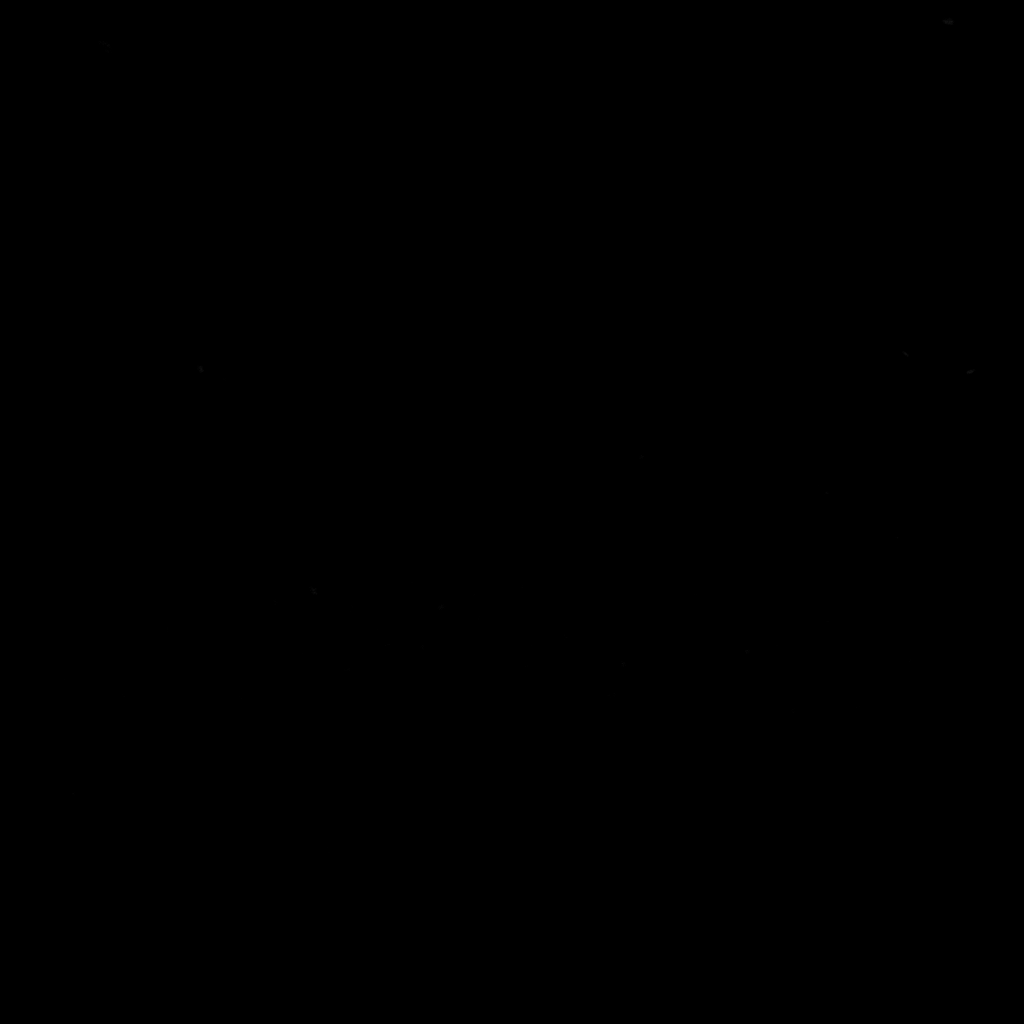

Supplement: Figure 4—figure supplement 2—source data 1. — The data were exported as 8-bit ‘tif’ files (1024 × 1024 pixels). Values for individual data points (and outlier analysis) for summary graphs in Figure 4—figure supplement 2B are Figure 4—figure supplement 2C contained in excel files. [file elife-62184-fig4-figsupp2-data1.zip › Figure 4- Figure supplement 2- Source Data 1/WT3/P42WT3.mglur6.tif]

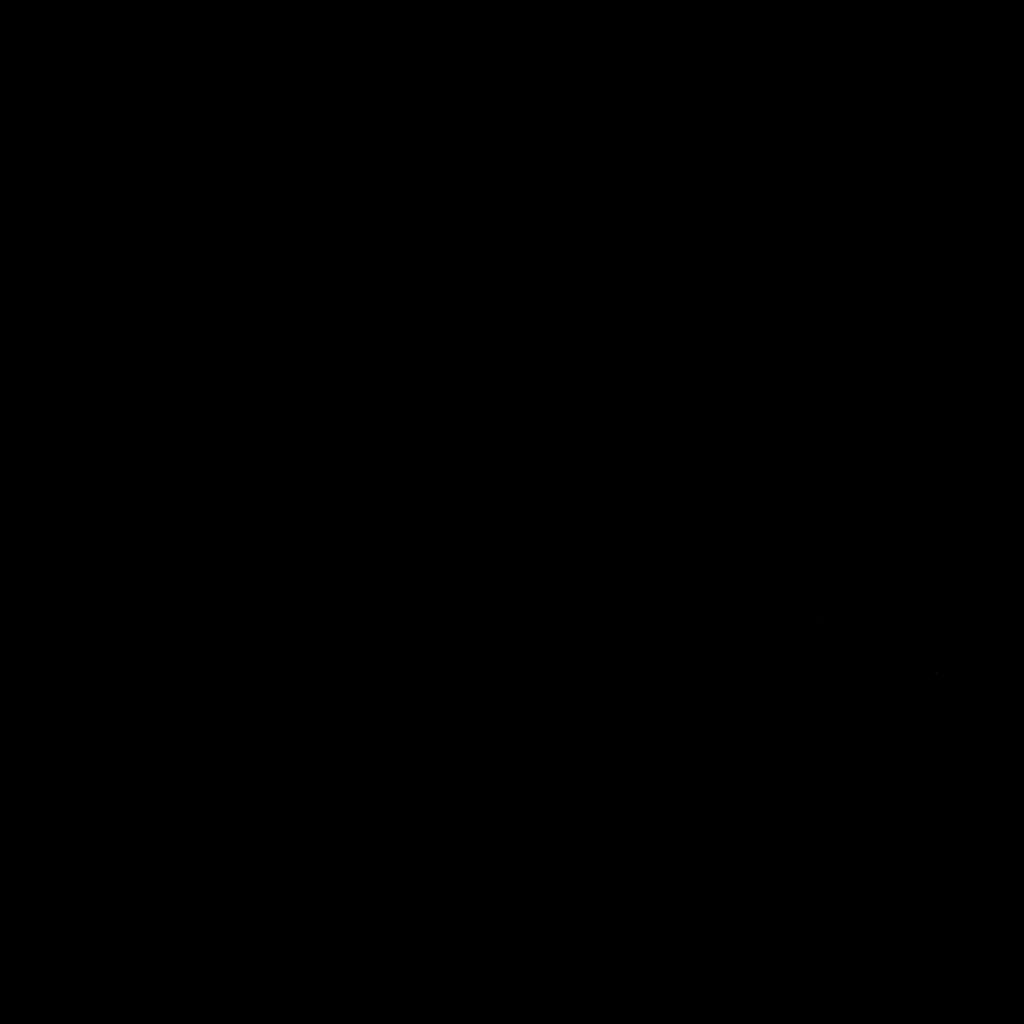

Supplement: Figure 4—figure supplement 2—source data 1. — The data were exported as 8-bit ‘tif’ files (1024 × 1024 pixels). Values for individual data points (and outlier analysis) for summary graphs in Figure 4—figure supplement 2B are Figure 4—figure supplement 2C contained in excel files. [file elife-62184-fig4-figsupp2-data1.zip › Figure 4- Figure supplement 2- Source Data 1/WT3/P42WT3.ribeye.tif]

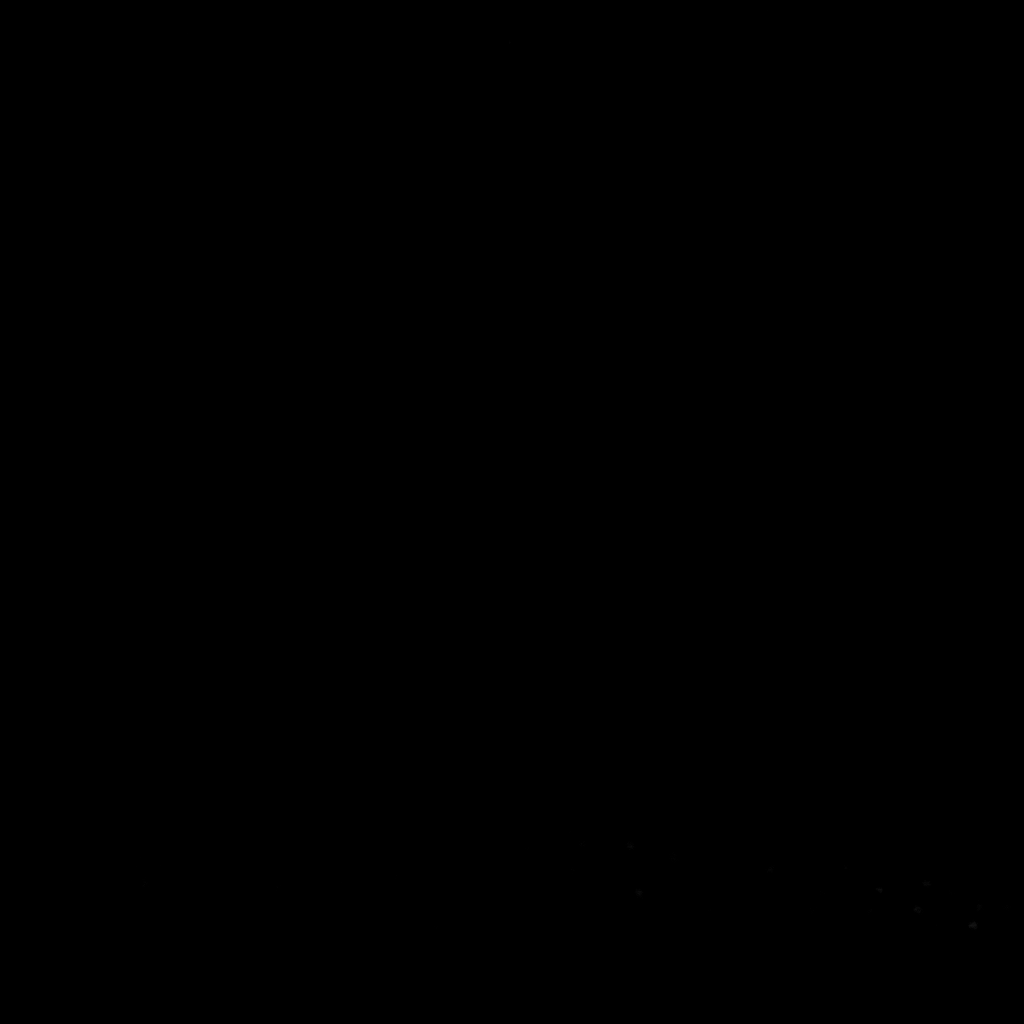

Supplement: Figure 4—figure supplement 2—source data 1. — The data were exported as 8-bit ‘tif’ files (1024 × 1024 pixels). Values for individual data points (and outlier analysis) for summary graphs in Figure 4—figure supplement 2B are Figure 4—figure supplement 2C contained in excel files. [file elife-62184-fig4-figsupp2-data1.zip › Figure 4- Figure supplement 2- Source Data 1/WT4/P42WT6.mglur6.tif]

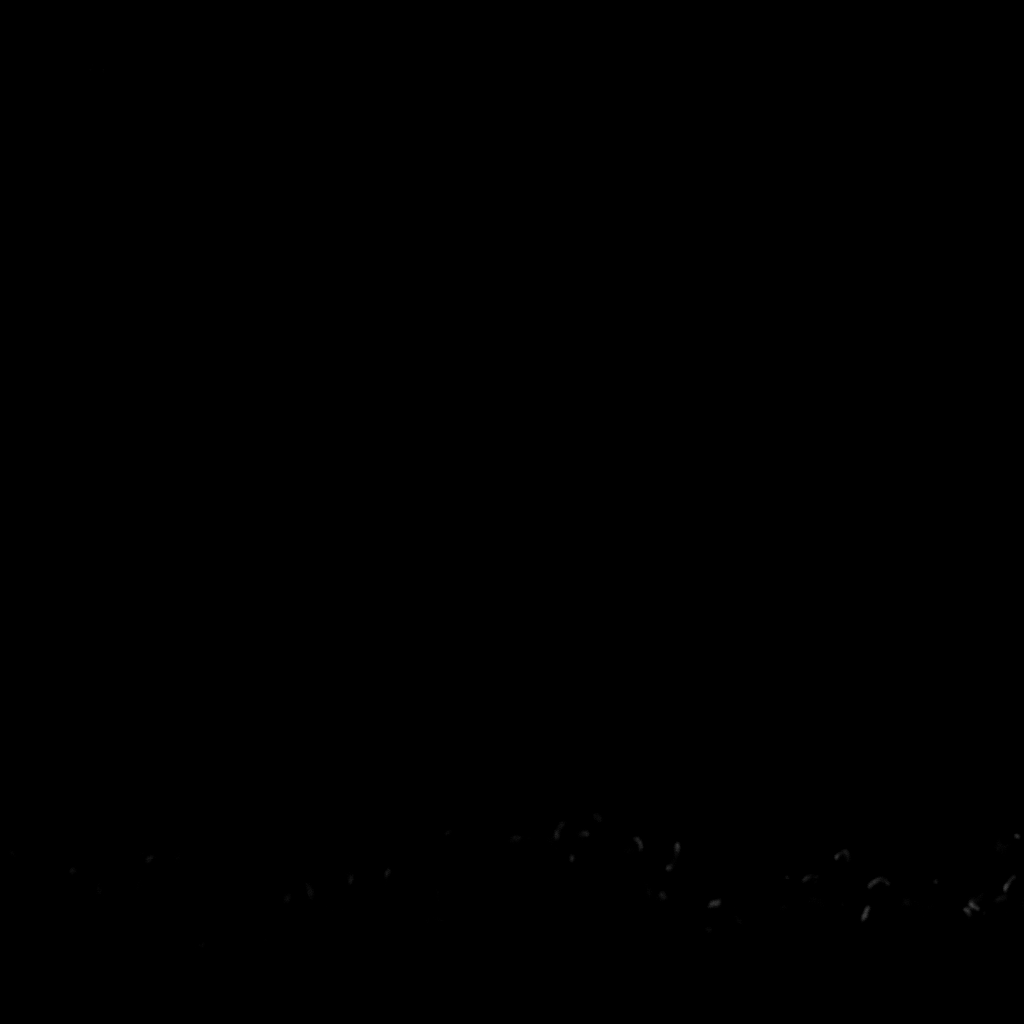

Supplement: Figure 4—figure supplement 2—source data 1. — The data were exported as 8-bit ‘tif’ files (1024 × 1024 pixels). Values for individual data points (and outlier analysis) for summary graphs in Figure 4—figure supplement 2B are Figure 4—figure supplement 2C contained in excel files. [file elife-62184-fig4-figsupp2-data1.zip › Figure 4- Figure supplement 2- Source Data 1/WT4/P42WT6.ribeye.tif]
